# UPLC-PDA-QTOFMS-guided isolation of prenylated xanthones and benzoylphloroglucinols from the leaves of *Garcinia oblongifolia* and their migration-inhibitory activity

**DOI:** 10.1038/srep35789

**Published:** 2016-10-21

**Authors:** Hong Zhang, Zheng Dan, Zhi-Jie Ding, Yuan-Zhi Lao, Hong-Sheng Tan, Hong-Xi Xu

**Affiliations:** 1 School of Pharmacy, Shanghai University of Traditional Chinese Medicine, Shanghai, 201203, P.R. China; 2 Engineering Research Centre of Shanghai Colleges for TCM New Drug Discovery, Shanghai, 201203, P.R. China

## Abstract

A UPLC-PDA-QTOFMS-guided isolation strategy was employed to screen and track potentially new compounds from *Garcinia oblongifolia*. As a result, two new prenylated xanthones, oblongixanthones D and E (**1**–**2**), six new prenylated benzoylphloroglucinol derivatives, oblongifolins V–Z (**3**–**7**) and oblongifolin AA (**8**), as well as a known compound oblongifolin L (**9**), were isolated from the EtOAc-soluble fraction of an acetone extract of the leaves of *Garcinia oblongifolia* guided by UPLC-PDA-QTOFMS analysis. The structures of the new compounds were elucidated by 1D- and 2D-NMR spectroscopic analysis and mass spectrometry. Experimental and calculated ECD spectra were used to determine the absolute configurations. The results of wound healing and transwell migration assay showed that oblongixanthones D (**1**), E (**2**), and oblongifolin L (**9**) have the ability to inhibit cancer cell migration in lower cytotoxic concentrations. Western blotting results showed that these compounds exhibited an anti-metastasis effect mainly through downregulating RAF protein levels. In addition, **2** and **9** could inhibit phospho-MEK and phospho-ERK at downstream. Moreover, **1**, **2**, and **9** could inhibit snail protein level, suggesting that they could regulate the EMT pathway.

*Garcinia oblongifolia* Champ. ex Benth. (Clusiaceae), a tree or medium-sized shrub with edible fruits, is widely distributed in tropical regions of southern mainland China and northern Vietnam. It has been used for many years as a folk medicine in China to treat stomatitis, eczema, and burns^1^,^2^. Previous phytochemical studies of this species led to the discovery of many new compounds, including prenylated benzoylphloroglucinols^3^,^4^,^5^,^6^, xanthones^4^,^5^, biphenyls^2^, and a new bixanthone derivative, garciobioxanthone^7^, which have been reported to possess a wide range of biological activities, including antitumor^1^,^8^,^9^,^10^, anti-inflammatory^11^, and antienteroviral effects^3^,^12^.

In our previous study, a series of new prenylated benzoylphloroglucinols, oblongifolins J–U were isolated from the bioacitve petroleum ether-soluble part of the acetone extract of *G. oblongifolia* leaves by bioassay-guided fractionation, in which two compounds exhibited significant anti-EV71 activity^3^. In our efforts to identify more new bioactive compounds from *Garcinia* species, the EtOAc-soluble fraction of the acetone extract of the leaves of *G. oblongifolia* was found to contain many potentially new compounds by UPLC-PDA-QTOFMS analysis. In follow-up work, UPLC-PDA-QTOFMS-guided fractionation led to the isolation of two new prenylated xanthones, oblongixanthones D and E (**1**–**2**), six new prenylated benzoylphloroglucinol derivatives, oblongifolins V–Z (**3**–**7**) and oblongifolin AA (**8**), as well as a known compound, oblongifolin L (**9**) from this fraction (Fig. 1). In this report, the isolation and structural elucidation of these compounds, as well as their migration-inhibitory activity in esophageal cancer are described.

## Results and Discussion

The EtOAc-soluble fraction of the acetone extract of the leaves of *Garcinia oblongifolia* and its subfractions were analyzed by UPLC-PDA-QTOFMS in positive mode to screen and characterize potentially new compounds. As a result, 13 unknowns of interest were detected from this extract and its subfractions by analysis of their UV, MS, and MS/MS spectra (Figures S1–S13, Supplementary information). Compounds **1** and **2** both showed the molecular ion [M + H]^+^ at *m*/*z* 447, corresponding to a molecular formula of C_24_H_30_O_8_. Their UV spectra showed similar absorption bands at approximately 257 and 327 nm, indicating that they might be xanthone derivatives (Figures S1 and S2)^13^. Compounds **3**–**8** and **P10**–**P14** produce the characteristic fragment ion at *m*/*z* 165.0182 or 177.0182 in their MS/MS spectra (Figures S3–S13), indicating that they might be polycyclic polyprenylated acylphloroglucinols (PPAPs)^14^,^15^,^16^. Their molecular formulas were obtained by accurate mass measurements for precursor ions. These 13 compounds were searched using the molecular formula and further refined by their skeletons in the SciFinder database. The results indicated that they were potentially new xanthones or PPAPs. Thus, they were selected as the targets of interest to be subjected to further phytochemical isolation. Finally, 8 new compounds (**1**–**8**) were isolated by column chromatography over MCI gel, silica gel, reversed-phase C_18_ silica gel, Sephadex LH-20, and preparative HPLC guided by UPLC-PDA-QTOFMS analysis. Other five potentially new PPAPs (**P10**–**P14**) could not been obtained, which were decomposed or oxidized during the isolation procedure or the NMR experiments. In addition, a known compound was obtained, which could be unambiguously identified as oblongifloin L (**9**) by comparison of the retention time (RT) and MS/MS data with those of the reference compound. Structural elucidation of the new compounds was achieved by HRMS, ^1^H NMR, ^13^C NMR, DEPT, HSQC, HMBC, and NOSEY analyses.

### Structural elucidation

**Figure 2:**
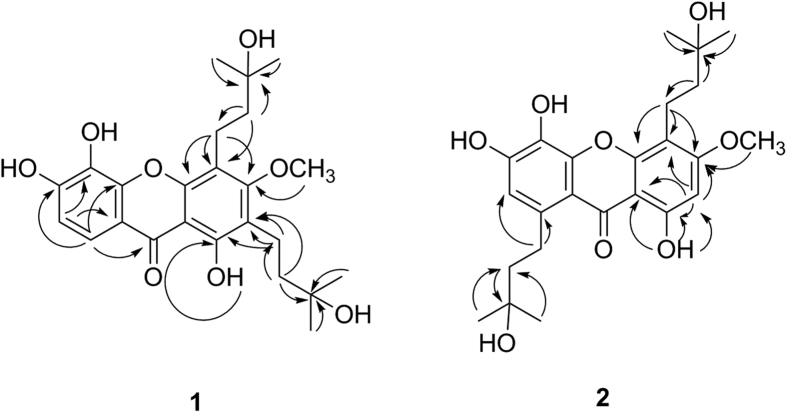
Key correlations observed in the HMBC NMR spectra of 1 and 2.

Oblongixanthone D (**1**) was isolated as yellow amorphous powder. In the MS/MS spectrum of **1**, the most abundant fragment peak (*m*/*z* 373) was due to the successive loss of an H_2_O and a prenyl unit C_4_H_8_ (56 Da). This fragment further dissociated by the loss of an H_2_O and a prenyl unit C_4_H_8_ to produce additional fragment ions at *m*/*z* 355 and 287. These fragmentaion characteristics indicated that **1** might contain two hydroxylated prenyl groups. The ^13^C NMR and DEPT spectra showed 12 aromatic carbons (including five oxygenated), one carbonyl carbon, two oxygenated quaternary carbons, four sp^3^ methylene carbons, four sp^3^ methyl groups, and one methoxyl group. The ^1^H NMR spectrum of **1** showed a chelated hydroxyl proton signal at *δ*_H_ 13.28 (1H, s), a methoxy signal at *δ*_H_ 3.82 (3H, s), two *ortho*-coupled aromatic proton signals at *δ*_H_ 7.54 (1H, d, *J* = 8.6 Hz) and 6.93 (1H, d, *J* = 8.6 Hz), and two 3-hydroxy-3-methylbutyl moiety singals, one showing a pair of gemdimethyl signals at *δ*_H_ 1.18 (6H, s) and two methylene signals at *δ*_H_ 2.64 (2H, m) and 1.59 (2H, m). The other 3-hydroxy-3-methylbutyl group exhibited a pair of gem-dimethyl signals at *δ*_H_ 1.24 (6H, s) and two methylene signals at *δ*_H_ 2.86 (2H, m) and 1.70 (2H, m). Based on the abovementioned information, compound **1** was proposed as a xanthone bearing a methoxy group and two 3-hydroxy-3-methylbutyl groups. The locations of two 3-hydroxy-3-methylbutyl moieties and a methoxy group were assigned to C-2 (*δ*_C_ 117.3), C-4 (*δ*_C_ 114.1), and C-3 (*δ*_C_ 162.7), respectively, based on the correlations from the methoxy protons at *δ*_H_ 3.82 to C-3, from H-11 (*δ*_H_ 2.64) to C-3, C-4, and C-4a (*δ*_C_ 152.3), as well as from H-16 (*δ*_H_ 2.86) to C-1 (*δ*_C_ 157.8) and C-2 in the HMBC spectrum (Fig. 2). Furthermore, the HMBC correlations from aromatic proton at *δ*_H_ 7.54 to C-6 (*δ*_C_ 146.4), C-10a (*δ*_C_ 152.9), and C-9 (*δ*_C_ 180.8), and from aromatic proton at *δ*_H_ 6.93 to C-5 (*δ*_C_ 132.6) and C-8a (*δ*_C_ 112.5) indicated that the locations of those two *ortho*-coupled aromatic protons were C-7 (*δ*_C_ 113.4) and C-8 (*δ*_C_ 116.2). Hence, the structure of **1** was assigned as 1,5,6-trihydroxy-3-methoxy-2,4-bis(3-hydroxy-3-methylbutyl)xanth-6-one.

The UV and NMR spectra of oblongixanthone E (**2**) were similar to those of **1**, suggesting that **2** was also a xanthone with one methoxy group and two 3-hydroxy-3-methylbutyl groups (Figures S1, S2, and Table 1). However, the MS/MS spectrum of **2** is different from that of **1** (Figures S1 and S2). Furthermore, comparison of the NMR data of **2** with those of **1** suggested that **2** is a 1,3,4,5,6,8-hexsubstituted xanthone [*δ*_H_ 6.69 (1H, s) and 6.43 (1H, s)], compared to 1,2,3,4,5,6-hexsubstituted ring system of **1** [*δ*_H_ 7.54 (1H, d, *J* = 8.6 Hz) and 6.93 (1H, d, *J* = 8.6 Hz)]. HMBC correlations between 1-OH/C-1, 1-OH/C-2, 1-OH/C-3, H-2/C-1, H-2/C-3, OCH_3_/C-3, H-11/C-3, H-11/C-4, H-11/C-4a, H-16/C-7, and H-16/C-8 indicated that the locations of two 3-hydroxy-3-methylbutyl moieties, a methoxy group, and two aromatic protons were at C-4 (*δ*_C_ 107.7), C-8 (*δ*_C_ 136.8), C-3 (*δ*_C_ 162.9), C-2 (*δ*_C_ 93.9), and C-7 (*δ*_C_ 114.5), respectively (Fig. 2). Therefore, **2** was determined to be 1,5,6-trihydroxy-3-methoxy-4,8-bis(3-hydroxy-3-methylbutyl)xanth-6-one.

**Figure 3:**
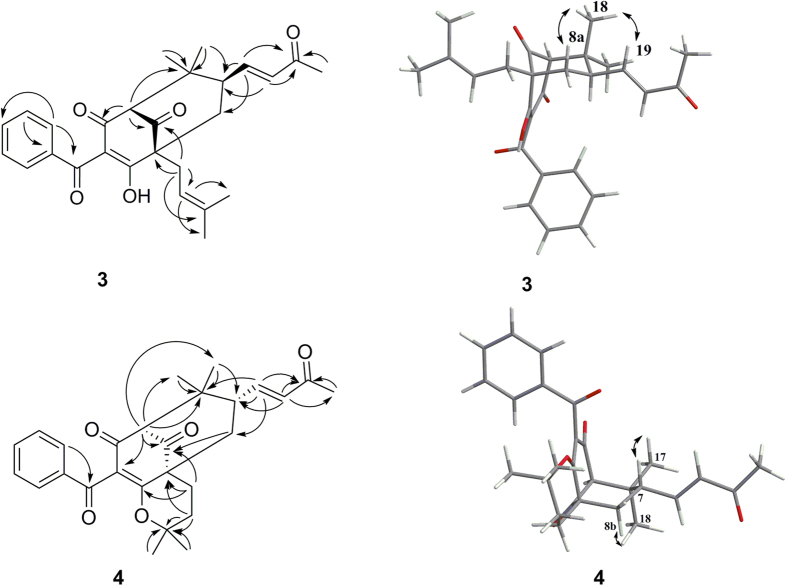
Key correlations observed in the HMBC and NOESY NMR spectra of 3 and 4.

Oblongifolin V (**3**) was obtained as a pale yellow gum. The molecular formula C_27_H_30_O_5_ was established by HRESIMS analysis and NMR spectroscopic data. The MS/MS spectrum of **3** showed a diagnostic fragment ion at *m*/*z* 165.0182 in the positive mode, suggesting that it might be a type B PPAP with no prenyl substituent at C-5^14^ (Figure S3). The ^1^H NMR spectrum indicated the presence of a monosubstituted benzene ring [*δ*_H_ 7.56 (1H, m), 7.42 (2H, m), and 7.59 (2H, m)], an *E*-configured carbon-carbon double bond [*δ*_H_ 6.71 (1H, dd, *J* = 15.7, 7.7 Hz) and 6.16 (1H, d, *J* = 15.7 Hz)], a vinyl proton [*δ*_H_ 5.18 (1H, brs)], two methylenes [*δ*_H_ 1.94, 1.89, 2.50, and 2.44], two methines [*δ*_H_ 3.13 (1H, s), 2.67 (1H, m)], and five methyl groups [*δ*_H_ 2.65 (3H, s), 1.72 (3H, s), 1.63 (3H, s), 1.17 (3H, s), and 1.02 (3H, s)]. The ^13^C NMR and DEPT spectra showed signals pertaining to a nonconjugated ketone (*δ*_C_ 206.8), an enolized 1,3-diketone (*δ*_C_ 196.5, 119.5, and 184.8), two quaternary carbons (*δ*_C_ 64.4 and 44.9), a methylene (*δ*_C_ 41.9), and two methines (*δ*_C_ 69.6 and 45.2), which allowed us to identify the bicyclo[3.3.1] nonane ring system typical of the prenylated benzoylphloroglucinols^17^. Resonances for six aromatic carbons and a conjugated carbonyl group (*δ*_C_ 198.1) were also observed, revealing the occurrence of a benzoyl group. In the HMBC spectrum, the long range correlations observed from the olefinic H-19 (*δ*_H_ 6.71, dd, *J* = 15.7, 7.7 Hz) signal to C-7 (*δ*_C_ 45.2) and C-8 (*δ*_C_ 41.9), along with correlations from olefinic H-20 (*δ*_H_ 6.16, d, *J* = 15.7 Hz) to the ketone resonance at *δ*_C_ 200.8 (C-21), which was in turn correlated with CH_3_-22, established a 3-buten-2-one side chain located at C-7. Other key HMBC correlations are shown in the Fig. 3. Based on these data, compound **3** was elucidated as a prenylated benzoylphloroglucinol with a prenyl unit at C-1 (*δ*_C_ 64.4), a 3-buten-2-one side chain at C-7, and no prenyl unit at C-5 (*δ*_C_ 69.6).

**Figure 4:**
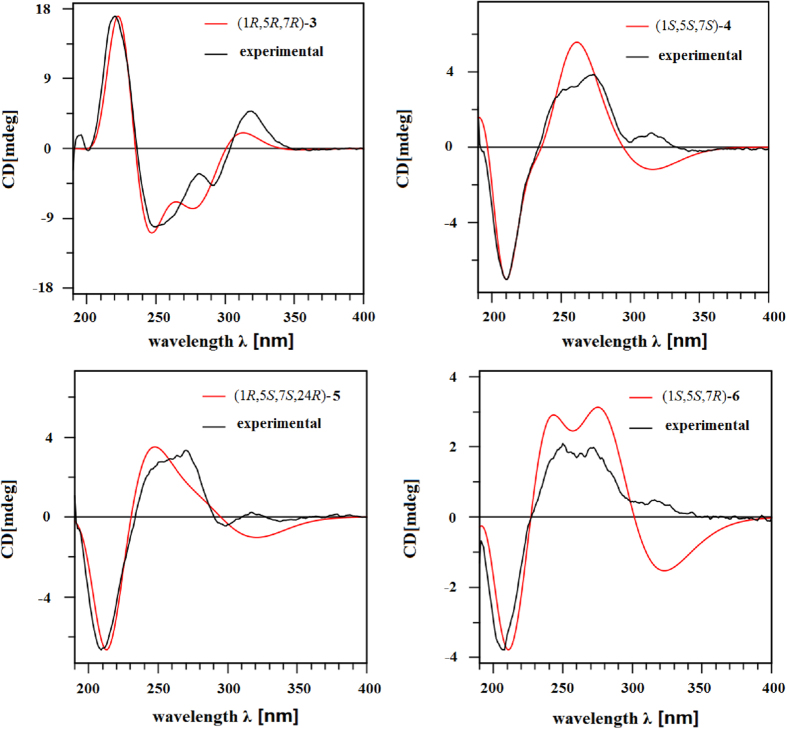
Calculated ECD spectra of 3–6 and their experimental curves.

The relative configuration of **3** was defined via a NOESY experiment. The correlations between H-19 (*δ*_H_ 6.71)/H-18 (*δ*_H_ 1.02) and H-8a (*δ*_H_ 1.94)/H-18 showed that the orientation of the 3-buten-2-one group at C-7 was *β* (Fig. 3). Thus, there were two possible isomers: (1*R*,5*R*,7*R*)-**3** and (1*S*,5*S*,7*S*)-**3**. The absolute configuration of **3** was determined by comparison of its experimentally measured electronic circular dichroism (ECD) curve with curves calculated using the TDDFT method. Consequently, the overall pattern of the calculated ECD curve of (1*R*,5*R*,7*R*)-**3** was consistent with the experimental ECD spectrum of **3** (Fig. 4). Therefore, the structure of oblongifolin V (**3**) was established as shown.

Oblongifolin W (**4**) gave the same molecular formula (C_27_H_30_O_5_) as **3** (*m*/*z* 435.2173 [M + H]^+^ (calcd 435.2166)) by HRESIMS. Comparison of its NMR data (Table 2) with those of **3** revealed these compounds to be closely related, except for differences between C-23 (*δ*_C_ 23.4), C-24 (*δ*_C_ 30.9), C-25 (*δ*_C_ 84.8), C-26 (*δ*_C_ 29.8), and C-27 (*δ*_C_ 26.7) of **4** and C-23 (*δ*_C_ 31.2), C-24 (*δ*_C_ 120.8), C-25 (*δ*_C_ 135.8), C-26 (*δ*_C_ 26.4), and C-27 (*δ*_C_ 18.4) of **3**. This finding indicated the presence of a 2,2-dimethyl-pyran moiety in **4** instead of a 3-methylbut-2-enyl side chain in **3**. HMBC correlations between H-23/C-1, H-23/C-2, H-23/C-9, H-23/C-25, H-24/C-1, H-24/C-25, H-26/C-25, H-27/C-25, and H-27/C-24 confirmed the presence of the 2,2-dimethyl-pyran moiety fused at C-1 (*δ*_C_ 50.2) and C-2 (*δ*_C_ 174.5). Key HMBC correlations are shown in Fig. 3. The relative configuration of **4** was assigned using NOE correlations, as shown in Fig. 3. Thus, two possible isomers, (1*R*,5*R*,7*R*)-**4** and (1*S*,5*S*,7*S*)-**4**, were considered, and the ECD spectra were calculated. Visual inspection suggested that the (1*S*,5*S*,7*S*)-**4** curve was similar to the experimental curve (Fig. 4). Thus, the structure of **4** was designated as shown.

The molecular formula of oblongifolin X (**5**) was determined to be C_27_H_28_O_5_ by HRESIMS. The ^1^H and ^13^C NMR data showed close similarities to those of **4**, with the exception of differences at C-23 (**5**: *δ*_C_ 34.3, CH_2_; **4**: *δ*_C_ 23.4, CH_2_), C-24 (**5**: *δ*_C_ 91.1, CH; **4**: *δ*_C_ 30.9, CH_2_), C-25 (**5**: *δ*_C_ 142.5, C; **4**: *δ*_C_ 84.8, C), C-26 (**5**: *δ*_C_ 114.9, CH_2_; **4**: *δ*_C_ 29.8, CH_3_), and C-27 (**5**: *δ*_C_ 17.3, CH_3_; **4**: *δ*_C_ 26.7, CH_3_). HMBC correlations between H-23/C-1, H-23/C-2, H-23/C-8, H-23/C-9, H-23/C-24, H-24/C-23, H-24/C-25, H-24/C-26, H-24/C-27, H-26/C-24, H-26/C-25, H-27/C-24, H-27/C-25, and H-27/C-26 confirmed the presence of a (2-isopropenyl)dihydrofuran ring moiety fused with the phloroglucinol moiety at C-1 (*δ*_C_ 61.7) and C-2 (*δ*_C_ 178.9) (Figure S14, Supplementary information). These data confirmed that a (2-isopropenyl)dihydrofuran ring moiety at C-1 and C-2 of **5** replaced the 2,2-dimethyl-pyran moiety in **4**. The planar structure of **5** was further elucidated by careful interpretation of the 2D NMR experiments. The relative configuration of **5** was determined by a NOESY experiment. Key NOE correlations are shown in Figure S14. Furthermore, the calculated ECD curve (Fig. 4) for **5** led to the absolute structure being determined as shown.

Oblongifolin Y (**6**) was obtained as a pale yellow gum, and its molecular formula of C_25_H_28_O_6_ was established by the positive HRESIMS ion at *m*/*z* 425.1969 [M + H]^+^ (calcd for C_25_H_29_O_6_, 425.1959). Comparison of the spectroscopic data of **6** with those of **4** showed that **6** is also a benzoylphloroglucinol with a 2,2-dimethyl-pyran moiety fused to the phloroglucinol moiety at C-1 and C-2. The only structural difference between the two compounds was the presence of an acetoxy group (COOH–CH_2_–) in **6** instead of the 3-buten-2-one group in **4**. The location of the acetoxy group was assigned to C-7 by the HMBC correlations from H-7 (*δ*_H_ 2.74) to C-19 (*δ*_C_ 34.3), as well as H-19 (*δ*_H_ 2.65) to C-20 (*δ*_C_ 173.9). The structure of **6** was confirmed by DEPT, HSQC, HMBC, and NOESY experiments. The key HMBC and NOE correlations are shown in Figure S15 (Supplementary information). Furthermore, an ECD experiment and ECD calculation of 6 were conducted. The experimental ECD spectrum of **6** was consistent with the calculated ECD spectra for (1*S*,5*S*,7*R*)-**6** (Fig. 4), thus establishing the assignment of the absolute configuration of **6** as depicted.

Oblongifolin Z (**7**) was obtained as a pale yellow gum. The molecular formula C_33_H_44_O_5_ for **7** was calculated from the HREIMS of the ion peak [M + Na]^+^ *m*/*z* 543.3083 (calc. 543.3081). The ^1^H NMR data of this compound were similar to values obtained for the known compound oblongifolin Q^3^, except for differences between H-30 [*δ*_H_ 1.44 (1H, m) and 1.34 (1H, m)] and H-32 [*δ*_H_ 1.12 (3H, s)] in **7** and H-30 [*δ*_H_ 3.95 (1H, t, *J* = 6.1 Hz)] and H-32 [*δ*_H_ 4.89 (1H, s) and 4.80 (1H, s)] in oblongifolin Q. This information indicated the occurrence of a 3-hydroxy-3-methylbutyl group at C-23 in **7** instead of the 2-hydroxy-3-methylbutenyl group of oblongifolin Q. This conclusion was further confirmed by the ^13^C NMR data of C-29 (*δ*_C_ 23.5, CH_2_), C-30 (*δ*_C_ 44.3, CH_2_), C-31 (*δ*_C_ 71.6, C), and C-33 (*δ*_C_ 29.29, CH_3_) and HMBC correlations between H-30/C-33, H-32/C-30, H-32/C-31, H-32/C-33, H-33/C-30, H-33/C-31, and H-33/C-32 (Figure S16, Supplementary information). According to the observed NOE correlations (Figure S16), the relative configuration of **7** was deduced to be the same as those of oblongifolin Q. In addition, the experimental ECD spectrum of **7** was consistent with that of **7** (Figure S17), suggesting that the absolute configuration of **7** is the same as that of oblongifolin Q. Thus, the structure of oblongifolin Z (**7**) was established as shown.

Oblongifolin AA (**8**) had the same molecular formula as the known compound **9** (C_33_H_44_O_4_), which was deduced from HRESIMS. The MS/MS spectrum of **8** in positive was also in near agreement with that of **9** (Figure S18, Supplementary information). The ^1^H and ^13^C NMR data (Table 3) showed close similarities to those of **9**, with the exception of significant differences in the chemical shifts at C-7, C-8, C-17, and C-18 due to the opposite configuration of C-7 [*δ*_C_ 45.5, 41.0, 25.4, and 31.5 for **8**, respectively, versus *δ*_C_ 42.4, 43.2, 27.6, and 21.2 for oblongifolin L, respectively]. These data indicated that **8** was a C-7 epimer of oblongifolin L. The structure of **8** was confirmed by DEPT, HSQC, HMBC, and NOESY experiments. The absolute configuration of **8** was also assigned by experimental and theoretically calculated ECD methods. As shown in Figure S19, the absolute configuration of **8** was determined as shown.

### Compounds 1, 2, and 9 inhibit migration in TE1 cells

**Figure 5:**
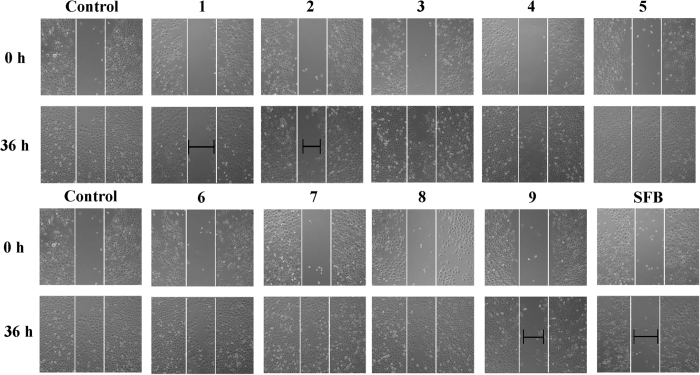
Anti-migration effect of compounds on human esophageal carcinoma cells (TE1) by wound healing assay. TE1 cells monolayer was scratched and treated with isolates at concentration of 20 μM. Sorafenib (SFB) was used as the positive control. Images were accessed by a microscope.

Our previous studies showed that compounds isolated from *Garcinia* species had potent inhibitory effect on esophageal cancer cell metastasis^10^,^18^. In addition, the EtOAc-soluble fraction of the actone extract of *G. oblongifolia* leaves could inhibit TE1 cell migration at concentrations of 12.5, 25 and 50 μg/ml (Figure S20). Therefore, all isolates were screened by wound healing assay to identify new compounds with anti-metastasis activity in esophageal cancer cells. As shown in Fig. 5, after treatment with various compounds at a concentration of 20 μM, compared to the positive control sorafenib (SFB), compounds **1**, **2**, and **9** exhibited the ability to inhibit migration in TE1 cells. The cell cytotoxicity tested by MTT assay showed that all compounds had lower cytotoxicity in TE1, KYSE150, HepG2, A549, and HL7702 cell lines (Table S1, Supplementary information). Moreover, the results indicated that TE1 cells were more sensitive to **1**, **2** and **9** than other cells.

**Figure 6:**
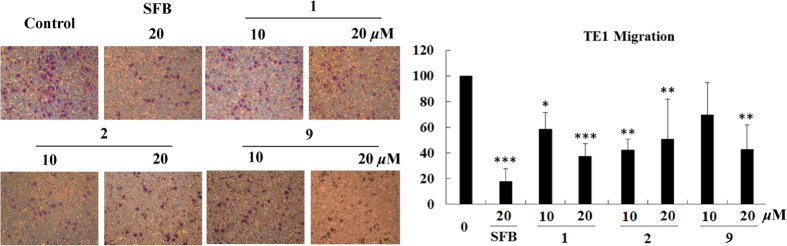
Compounds **1**, **2**, and **9** inhibit migration in TE1 cells measured by transwell assay. Cells were incubated with **1**, **2**, or **9** for 36 h, and migrated cells were fixed and stained with 0.1% crystal violet. Sorafenib (SFB) was used as the positive control. The summary data for transwell migration assay were presented as the means ± S.D. *P < 0.05, **P < 0.01, ***P < 0.001.

Transwell assay was performed to confirm the effect of all isolates on cell migration. After being incubated with these compounds at 10 and 20 μM for 24 h, TE1 cells were fixed and imaged, and the number of migrated cells was counted. As shown in Fig. 6, the migrated cells were significantly reduced after compounds **1**, **2** and **9** treatments. Statistical data also suggested that **1**, **2** and **9** could significantly inhibit cell migration. In addition, compounds **3**–**8** had no significant effect on cell migration, which was consistent with the results of wound healing assay (Figure S21). A transwell invasion assay was preformed to investigate whether the invasion ability was blocked by **1**, **2** and **9**. The results suggested that **9** significantly attenuated TE1 cells invasion. Moreover, compounds **1** and **2** did not inhibit cell invasion in the matrigel invasion assay (Figure S22, Supplementary information). All these results suggest that **1**, **2** and **9** showed a potential effect on cell metastasis.

### Compounds 1, 2, and 9 downregulate RAF-MAPK signaling pathway and snail protein level

**Figure 7:**
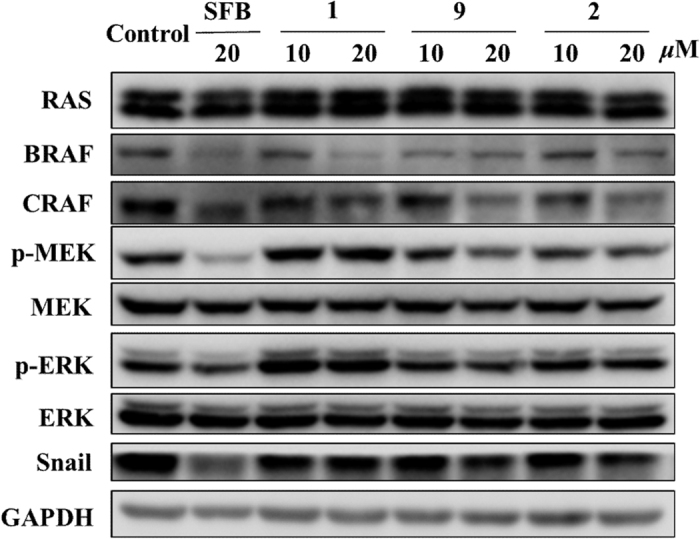
Compounds **1**, **2**, and **9** downregulate RAF-MAPK signaling pathway and snail protein level. RAS-RAF-MEK-ERK cascades and snail protein were analyzed by western blot. TE1 cells were treated with **1**, **2**, or **9** with various concentrations (0, 10, 20 μM) for 48 h. Sorafenib (SFB) was used as the positive control.

The RAF–MEK–ERK cascade plays a central role in the regulation of cell metastasis, proliferation and survival. The high frequency of this pathway deregulated in human cancer makes it an attractive target for drug development^19^. Therefore, we examined whether these compounds with anti-metastasis activity could interfere RAF-MAPK cascades. Western blot showed that the 24 h treatment of **1**, **2**, and **9** attenuated BRAF and CRAF protein levels at both 10 and 20 μM (Fig. 7). In addition, compounds **2** and **9** inhibited phospho-MEK and phospho-ERK, suggesting that these two compounds could also interfere downstream signals. No significant changes were detected on RAS, MEK and ERK proteins (Fig. 7), as well as on BRAF and CRAF mRNA levels (Figure S22, Supplementary information).

The epithelial-mesenchymal transition (EMT) is a fundamental process that acts on endowing cells with migratory and invasive properties. Snail is a protein that promotes EMT, acting as an EMT marker^20^. To illustrate whether these compounds could affect snail protein, we performed a western blot analysis. The results showed that **1**, **2**, and **9** reduced snail protein (Fig. 7), suggesting that they could regulate the EMT pathway. In conclusion, compounds **1**, **2**, and **9** could downregulate the RAF–MEK–ERK cascades and regulate EMT pathway by decreasing snail protein, indicating that these compounds could inhibit cell migration through multiple signaling pathways.

## Experimental Section

### General Experimental Procedures

Optical rotations were measured using an Autopol VI polarimeter. Ultraviolet absorption spectra were recorded on a UV-2401 PC spectrophotometer. ECD spectra were recorded on a Chirascan-plus spectrometer (Applied photophysics Ltd., Surrey, United Kingdom). IR spectra were obtained from a Perkin-Elmer 577 spectrometer. NMR spectra were measured on a Bruker AV-600 or AV-400 spectrometer and calibrated by the solvent peak used. Mass spectrometry was performed on a SYNAPT G2-Si HDMS (Waters Corp., Manchester, UK) with an electrospray ion source (Waters, Milford, MA) connected to a lock-mass apparatus, which performed real-time calibration correction. Column chromatography was performed with CHP20P MCI gel (75–150 μm, Mitsubishi Chemical Corporation, Japan), silica gel (100–200, or 200–300 mesh, Qingdao Haiyang Chemical Co., Ltd.), Sephadex LH-20 (GE Healthcare Bio-Sciences AB, Sweden), and reversed-phase C_18_ silica gel (50 μm, YMC, Kyoto, Japan). Precoated TLC sheets of silica gel 60 GF254 (Qingdao Haiyang Chemical Co., Ltd.) were used. A Waters 2535 Series machine equipped with a Xbridge C_18_ column (4.6 × 250 mm, 5 μm) was used for HPLC analysis, and a preparative Xbridge Prep C_18_ OBD column (19 × 250 mm, 5 μm) was used for the sample preparation.

### Plant Material

The leaves of *G. oblongifolia* were collected at Bobai, Guangxi Zhuang Autonomous Region, People’s Republic of China in December, 2005. The sample was identified by Dr. Chun-Feng Qiao. A voucher specimen (Herbarium No. 20120843) was deposited at the Engineering Research Centre of Shanghai Colleges for TCM New Drug Discovery, Shanghai University of Traditional Chinese Medicine.

### Extraction and Isolation

According to the previous separation process^3^, the EtOAc-soluble fraction (220 g) of an acetone extract of the leaves of *G. oblongifolia* has been obtained. A UPLC-PDA-QTOFMS-guided isolation approach was employed to track the potentially new compounds during the entire isolation process. The EtOAc-soluble fraction was subjected to column chromatography (CC) on MCI, and successively eluted with H_2_O, 30%, 95% EtOH, and EtOAc. The 95% EtOH-eluting fraction was separated by chromatography on a silica gel column using a gradient of petroleum ether–acetone (100:0 to 0:100, v/v) to yield 8 fractions A–K.

Fraction F (2.8 g) was subjected to reversed-phase C_18_ silica gel CC and eluted in a step gradient manner with MeOH-H_2_O (60:40 to 100:0) to obtain fifteen subfractions Fa–Fp. Subfraction Fb was purified by preparative HPLC (MeCN-H_2_O, 45:55, with 0.1% formic acid in H_2_O, 20 ml/min) to yield compound **3** (12.5 mg). Subfraction Fd was further separated by preparative HPLC (MeCN-H_2_O, 39:61, with 0.1% formic acid in H_2_O, 20 mL/min) to give compounds **4** (7.1 mg) and **5** (2.7 mg). Compounds **8** (27.3 mg) and **9** (50 mg) were purified by preparative HPLC (MeCN-H_2_O, 77:23, with 0.1% formic acid in H_2_O, 20 ml/min) from subfraction Fo. Subfraction H (1.2 g) was subjected to CC on a reversed-phase C_18_ silica gel eluted with MeOH-H_2_O in a gradient (40:60 to 100:0) to obtain 10 subfractions (Ha–Hj). Subfraction Hd was purified by preparative HPLC (MeCN-H_2_O, 40:60, with 0.1% formic acid in H_2_O, 20 ml/min) to yield compound **7** (8.1 mg). Fraction I (1.2 g) was chromatographed on a Sephadex LH-20 column (MeOH) to obtain 9 subfractions Ia-Ii. Fraction Ib (750 mg) was subjected to reversed-phase C_18_ silica gel CC and eluted in a step gradient manner with MeOH-H_2_O (50:50 to 100:0) to obtain subfractions Iba–Ibj. Subfractions Ibb, Ibc, and Ibd were purified by preparative HPLC to yield compounds **6** (5.8 mg), **1** (5 mg), and **2** (8.9 mg), respectively.

Oblongixanthone D (**1**): light yellow powder; UV (MeOH) *λ*_max_ (log*ε*) 256 (3.68), 328 (3.19) nm; IR (KBr) *ν*_max_ 3423, 2965, 2927, 1633, 1590, 1515, 1459, 1432,1301, 1213, 1110, 1037, 755 cm^−1^; ^1^H NMR (DMSO-*d*_6_, 600 MHz) data, see Table 1; ^13^C NMR (DMSO-*d*_6_, 150 MHz) data, see Table 1; HRESIMS 447.2013 [M + H]^+^ (calcd for C_24_H_31_O_8_, 447.2013).

Oblongixanthone E (**2**): light yellow powder; UV (MeOH) *λ*_max_ (log*ε*) 255 (3.77), 330 (3.39) nm; IR (KBr) *ν*_max_ 3428, 2965, 1646, 1523, 1444, 1384, 1321, 1115, 1105 cm^−1^; ^1^H NMR (DMSO-*d*_6_, 400 MHz) data, see Table 1; ^13^C NMR (DMSO-*d*_6_, 100 MHz) data, see Table 1; HRESIMS 447.2015 [M + H]^+^ (calcd for C_24_H_31_O_8_, 447.2013).

Oblongifolin V (**3**): light brown gum; [α]^25^_D_ 4.3 (*c* 0.02, MeOH); UV (MeOH) *λ*_max_ (log*ε*) 202 (4.10) nm; ECD (*c* 4.37 × 10^−4^ M, MeOH) *λ*_max_ nm (Δ*ε*) 220 (+22.98), 250 (−10.10), 291 (−4.75), 319 (+4.83); IR (KBr) *ν*_max_ 3359, 2921, 2850, 1722, 1633, 1590, 1573, 1467, 1382, 1280, 1025, 701 cm^−1^; ^1^H NMR (CD_3_OD/0.1% TFA, 600 MHz) data, see Table 2; ^13^C NMR (CD_3_OD/0.1% TFA, 150 MHz) data, see Table 2; HRESIMS 435.2169 [M + H]^+^ (calcd for C_27_H_31_O_5_, 435.2166).

Oblongifolin W (**4**): light brown gum; [α]^25^_D_ 20.0 (*c* 0.03, MeOH); UV (MeOH) *λ*_max_ (log*ε*) 253 (3.51) nm; ECD (*c* 2.42 × 10^−4^ M, MeOH) *λ*_max_ nm (Δ*ε*) 211 (−7.03), 273 (+3.87), 315 (+0.76); IR (KBr) *ν*_max_ 3359, 3197, 2956, 2923, 2852, 1733, 1677, 1633, 1596, 1467, 1365, 1233, 1118, 755 cm^−1^; ^1^H NMR (CD_3_OD/0.1% TFA, 600 MHz) data, see Table 2; ^13^C NMR (CD_3_OD/0.1% TFA, 150 MHz) data, see Table 2; HRESIMS 435.2173 [M + H]^+^ (calcd for C_27_H_31_O_5_, 435.2166).

Oblongifolin X (**5**): light brown gum; [α]^25^_D_ – 11.8 (*c* 0.03, MeOH); UV (MeOH) *λ*_max_ (log*ε*) 253 (3.80) nm; ECD (*c* 11.76 × 10^−4^ M, MeOH) *λ*_max_ nm (Δ*ε*) 209 (−6.64), 270 (3.34); IR (KBr) *ν*_max_ 3444, 2929, 1733, 1677, 1623, 1448, 1365, 1288, 1207, 1139, 1027 cm^−1^; ^1^H NMR (CD_3_OD/0.1% TFA, 600 MHz) data, see Table 2; ^13^C NMR (CD_3_OD/0.1% TFA, 150 MHz) data, see Table 2; HRESIMS 433.2010 [M + H]^+^ (calcd for C_27_H_29_O_5_, 433.2009).

Oblongifolin Y (**6**): light brown gum; [α]^25^_D_ 24.1 (*c* 0.06, MeOH); UV (MeOH) *λ*_max_ (log*ε*) 253 (3.40) nm; ECD (*c* 3.54 × 10^−4^ M, MeOH) *λ*_max_ nm (Δ*ε*) 207 (−3.78), 272 (3.39); IR (KBr) *ν*_max_ 3444, 2967, 1733, 1644, 1594, 1452, 1375, 1336, 1118 cm^−1^; ^1^H NMR (acetone-*d*_6_, 600 MHz) data, see Table 2; ^13^C NMR (acetone-*d*_6_, 150 MHz) data, see Table 2; HRESIMS 425.1969 [M + H]^+^ (calcd for C_25_H_29_O_6_, 425.1959).

Oblongifolin Z (**7**): light brown gum; [α]^25^_D_ – 23.5 (*c* 0.03, MeOH); UV (MeOH) *λ*_max_ (log*ε*) 244 (3.35), 282 (3.37) nm; ECD (*c* 2.60 × 10^−4^ M, MeOH) *λ*_max_ nm (Δ*ε*) 215 (4.97), 253 (−3.74), 319 (1.63); IR (KBr) *ν*_max_ 3444, 2929, 1718, 1670, 1635, 1590, 1573, 1382, 1078 cm^−1^; ^1^H NMR (CD_3_OD/0.1% TFA, 400 MHz) data, see Table 3; ^13^C NMR (CD_3_OD/0.1% TFA, 100 MHz) data, see Table 3; HRESIMS 543.3083 [M + Na]^+^ (calcd for C_33_H_44_O_5_Na, 543.3081).

Oblongifolin AA (**8**): light brown gum; [α]^25^_D_ – 36.2 (*c* 0.05, MeOH); UV (MeOH) *λ*_max_ (log*ε*) 253 (3.54), 286 (3.56) nm; ECD (*c* 2.60 × 10^−4^ M, MeOH) *λ*_max_ nm (Δ*ε*) 217 (21.85), 254 (−12.88), 316 (5.52); IR (KBr) *ν*_max_ 3444, 2962, 2923, 1729, 1673, 1637, 1590, 1573, 1448, 1382, 1286, 1025 cm^−1^; ^1^H NMR (CD_3_OD/0.1% TFA, 400 MHz) data, see Table 3; ^13^C NMR (CD_3_OD/0.1% TFA, 100 MHz) data, see Table 3; HRESIMS 503.3164 [M + H]^+^ (calcd for C_33_H_45_O_4_, 503.3167).

#### UPLC-PDA-QTOFMS analysis

UPLC was performed using a Waters Acquity UPLC Iclass system (Waters, Milford, MA, USA), equipped with a binary solvent delivery system, an autosampler, and a photodiode-array detection (PDA) system. Chromatography was performed on a Waters ACQUITY BEH C_18_ column (2.1 mm × 100 mm I.D., 1.7 μm, Waters, Milford, MA, USA). The mobile phase consisted of (A) 0.1% formic acid in water and (B) ACN containing 0.1% formic acid. The 0.1% formic acid was added to both the aqueous solution and ACN. The UPLC eluting conditions were as follows: 32–42% B (0–5 min), 42–46% B (5–10 min), 46–70% B (10–23 min), 70–95% B (23–29 min) and 95–100% B (29–32 min). The flow rate was maintained at 0.4 ml/min. The column and autosampler were maintained at 40 °C and 10 °C, respectively.

Mass spectra were obtained using a SYNAPT G2-Si HDMS (Waters Corp., Manchester, UK) equipped with an electrospray ionization source. ESI mass spectra were acquired over the *m*/*z* 50–1200 range. The desolvation gas was set to 800 L h^−1^ at temperature of 400 °C, the cone gas was set to 50 L h^−1^, and the source temperature was set to 120 °C. The capillary voltage was set to 2500 V. Spectra were acquired in continuum and positive mode. Argon was employed as the collision gas. The SYNAPT G2-Si HDMS system was calibrated using sodium formate clusters and operated in resolution mode. Leucine enkephalin was used as a lock mass. All fractions were analyzed by UPLC-QTOFMS using the data-dependent acquisition (DDA) mode. The top five ions were selected for MS/MS from a single MS survey scan. The scan time for MS/MS was 0.1 s. The collision energy in the trap cell was ramped from 15 to 40 V. UNIFI 1.8 was used for visualization, processing, and interpretation of MS data.

#### Cell culture

The human esophageal cancer cell line TE1 and the KYSE150 cells were provided from Fudan University Shanghai Cancer Center. The human cell line HepG2, A549 and HL7702 cells were purchased from the Shanghai Institute of Biochemistry and Cell Biology (Shanghai, China). Cells were grown in RPMI 1640 or DMEM medium (Hyclone, Logan, UT, USA) with 10% fetal bovine serum (FBS), 100 U/ml penicillin and 100 mg/ml streptomycin, cultivated at 37 °C containing 5% CO_2_.

#### Cell viability assay

The cell viability was assessed by an MTT assay. Briefly, 5 × 10^3^ cells were seeded into a 96-well culture plate. After 24 h, cells were incubated with various compounds for 48 h. Approximately 10 μl of MTT (5 mg/ml) was added and dimethyl sulfoxide was used to dissolve the formazan crystals. The absorbance was measured at 570 nm by a microplate reader.

#### Wound healing assay

Cell motility was evaluated by wound healing. Briefly, 1 × 10^5^ cells were seeded into a 24-well culture plate. When the cells grew to 80–90% confluence, a scratch was created through the cell monolayer by sterile 100 μl pipette tips, and media containing various dilutions of compounds were added. Cell migration was observed and imaged under an IX83 microscope (Olympus, Tokyo, Japan) after 36 h.

#### Transwell migration and invasion assay

Cell migration and invasion were estimated using transwell chambers (Corning, NY, USA) with a pore size of 8 μm. For the migration assay, 5 × 10^4^ cells were added into the upper chamber in serum-free medium, and in the bottom chamber, 600 μL of 10% FBS medium was added. After incubating with various concentrations of compounds for 24 h, the cells on the upper surface of the chamber were removed using cotton swabs. The migrated cells on the bottom surface were fixed in 4% paraformaldehyde, stained with 0.1% crystal violet and scored under a light microscope in five random fields. For the transwell invasion assay, the upper chamber membranes were pre-incubated with matrigel (BD Biosciences, Bedford, MA, USA) for 2 hours at 37 °C.

#### Quantitative Real-time PCR

After treatment, total RNA was extracted from TE1 cells using Trizol (Takara, Shiga, Japan). Then, RNA was reverse transcribed by a PrimeScript RT reagent kit. Quantitative PCR was conducted with forward and reverse primers containing SYBER Green. Then, real-time PCR was performed under a StepOnePlus Real-Time PCR System. The primers for human genes were as follows: for GAPDH, forward primer: 5′-TGTTGCCATCAATGACCCCTT-3′, reverse primer: 5′-CTCCACGACGTACTCAGCG-3′, for B-RAF, forward primer: 5′-CATTGGTTTTGATGAGTATATGAAC-3′, reverse primer: 5′-GGAGACACTTTGTAGCAGAG-3′ and for C-RAF, forward primer: 5′-TGAGCACTGTAGCACCAAAGTACCT-3′, reverse primer: 5′-CAGACTCTCGCATACGACGCAT-3′.

#### Western blot analysis

TE1 cells were seeded on a 3.5-cm dish, treated with various compounds, and lysed in RIPA buffer. Proteins were separated on SDS polyacrylamide gels and transferred to PVDF membranes (Millipore, Billerica, MA, USA). The membranes were blocked and immunoblotted with primary antibodies at 4 °C overnight, followed by appropriate secondary antibodies. GAPDH was used as the loading control. Membranes were visualized under an Image Quant LAS 4000 mini and processed by Image Quant TL 1D software (General Electric Company).

The primary antibodies C-RAF (Cat.9422), MEK1/2 (Cat.9122), p-MEK (Ser217/221, Cat.9154), ERK (Cat.4695), p-ERK (Tyr202/Tyr204, Cat.4370), GAPDH (Cat.5174) and snail (Cat.3879) were purchased from Cell Signaling Technologies (Danvers, MA, USA). RAS (Cat.ab137739) and B-RAF (Cat.ab33899) were purchased from Abcam (Cambridge, UK).

## Additional Information

**How to cite this article**: Zhang, H. *et al.* UPLC-PDA-QTOFMS-guided isolation of prenylated xanthones and benzoylphloroglucinols from the leaves of *Garcinia oblongifolia* and their migration-inhibitory activity. *Sci. Rep.* **6**, 35789; doi: 10.1038/srep35789 (2016).

## Supplementary Material

Supplementary Information

## Figures and Tables

**Figure 1:**
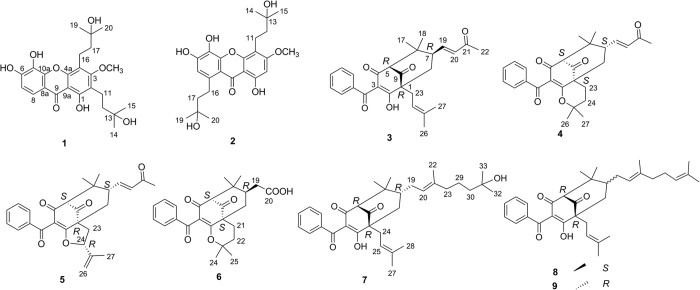
Compounds isolated from the leaves of *G. oblongifolia*.

**Table 1:** ^1^H and ^13^C NMR Data (DMSO-*d*_6_) for Compounds 1 and 2 a.

No.	1^b^		2^c^	
	*δ*_H_ (*J* in Hz)	*δ*_C_	*δ*_H_ (*J* in Hz)	*δ*_C_
1		157.8		161.1
2		117.3	6.43, s	93.9
3		162.7		162.9
4		114.1		107.7
4a		152.3		152.7
5		132.6		130.6
6		146.4		151.3
7	6.93, d (8.6)	113.4	6.69, s	114.5
8	7.54, d (8.6)	116.2		136.8
8a		112.5		110.1
9		180.8		182.2
9a		113.4		102.3
10a		152.9		147.7
11	2.64, m	18.2	2.81, m	16.9
12	1.59, m	43.0	1.55, m	42.7
13		68.8		69.2
14	1.18, s	29.1	1.19, s	29.2
15	1.18, s	29.1	1.19, s	29.2
16	2.86, m	18.3	3.11, m	29.7
17	1.70, m	43.2	1.59, m	45.6
18		69.3		68.9
19	1.24, s	29.1	1.17, s	29.0
20	1.24, s	29.1	1.17, s	29.0
5-OH	13.28, s		13.62, s	
OCH_3_	3.82, s	48.6	3.88, s	56.2

^a^Assignments based on DEPT, HSQC, and HMBC experiments; chemical shifts in ppm.

^b^Data were measured at 600 and 150 MHz.

^c^Data were measured at 400 and 100 MHz.

**Table 2:** ^1^H and ^13^C NMR Data (600 and 150 MHz) for Compounds **3**–**6** a.

Position	3^b^		4^b^		5^b^		6^c^	
	*δ*_H_ (*J* in Hz)	*δ*_C_	*δ*_H_ (*J* in Hz)	*δ*_C_	*δ*_H_ (*J* in Hz)	*δ*_C_	*δ*_H_ (*J* in Hz)	*δ*_C_
1		64.4		50.2		61.7		50.1
2		196.5		174.5		178.9		172.7
3		119.5		126.6		118.3		126.6
4		184.8		193.1		193.1		191.6
5	3.13, s	69.6	3.00, s	74.9	2.98, s	74.1	2.87, s	75.2
6		44.9		43.6		44.2		42.8
7	2.67, m	45.2	2.93, m	45.0	2.94, m	45.2	2.74, m	37.7
8	1.94, m	41.9	2.43, dd (14.0, 4.6)	38.6	2.40, dd (14.1, 4.8)	37.2	2.62, m;	39.4
	1.89, m		1.91, m		2.21, m		1.61, t (13.1)	
9		206.8		205.7		202.8		205.7
10		198.1		196.1		194.2		193.9
11		139.2		138.6		138.3		138.6
12	7.59, m	130.0	7.84, m	130.2	7.79, m	130.4	7.95, d (7.4)	129.9
13	7.42, m	129.3	7.52, m	130.0	7.50, m	130.0	7.50, t (7.6)	129.6
14	7.56, m	134.0	7.65, m	135.2	7.63, m	135.3	7.60, t (7.3)	134.1
15	7.42, m	129.3	7.52, m	130.0	7.50, m	130.0	7.50, t (7.6)	129.6
16	7.59, m	130.0	7.84, m	130.2	7.79, m	130.4	7.95, d (7.4)	129.9
17	1.17, s	27.9	1.09, s	27.5	1.13, s	27.6	1.09, s	26.8
18	1.02, s	21.6	0.98, s	21.3	1.02, s	21.5	0.91, s	20.7
19	6.71, dd (15.7, 7.7)	146.9	6.83, dd (15.9, 8.4)	146.3	6.83, dd (15.9, 8.3)	146.1	2.65, m	34.3
							2.18, m	
20	6.16, d (15.7)	134.5	6.30, d (15.9)	134.7	6.28, d (15.9)	134.7		173.9
21		200.8		200.7		200.8	2.45, m	23.1
							1.71, m	
22	2.65, s	26.4	2.31, s	27.5	2.29, s	27.5	2.03, m	30.8
							1.83, m	
23	2.50, m;	31.2	2.53, m;	23.4	2.59, dd (13.2, 11.2)	34.3		83.2
	2.44, m		1.79, m		2.21, m			
24	5.18, brs	120.8	2.07, m	30.9	5.39, dd (11.2, 5.3)	91.1	1.23, s	29.9
			1.80, m					
25		135.8		84.8		142.5	1.09, s	26.3
26	1.72, s	26.4	1.27, s	29.8	4.93, m	114.9		
27	1.63, s	18.4	0.99, s	26.7	1.60, s	17.3		

^a^Assignments based on DEPT, HSQC, HMBC, and NOESY experiments; chemical shifts in ppm.

^b^Measured in CD_3_OD/0.1% TFA.

^c^Measured in acetone-*d*_6_.

**Table 3:** ^1^H and ^13^C NMR Data (400 and 100 MHz, CD_3_OD/0.1% TFA) for Compounds 7 and 8.

Postion	7		8	
	*δ*_H_ (*J* in Hz)	*δ*_C_	*δ*_H_ (*J* in Hz)	*δ*_C_
1		65.3		63.2
2		197.2		196.9
3		119.0		117.1
4		185.3		188.5
5	3.08, s	70.1	3.03, s	68.3
6		45.4		46.3
7	1.74 m	42.5	1.55, m	45.5
8	2.02, m; 1.48, m	43.1	2.21, m; 2.18, m	41.0
9		207.6		208.8
10		198.1		197.9
11		139.1		139.0
12	7.59, m	129.9	7.55, m	129.9
13	7.41, m	129.2	7.41, m	129.2
14	7.55, m	133.9	7.55, m	133.9
15	7.41, m	129.2	7.41, m	129.2
16	7.59, m	129.9	7.55, m	129.9
17	1.24, s	27.5	1.28, s	25.4
18	0.99, s	21.2	1.17, s	31.5
19	2.17, m; 1.74, m	28.9	2.21, m; 2.08, m	30.4
20	5.06, m	124.0	4.90, m	125.1
21		138.3		137.8
22	1.58, s	16.3	1.57, s	16.4
23	1.98, m	41.3	2.04, m; 1.96, m	41.0
24	2.44, m	31.3	2.47, dd (13.9, 8.6)	31.7
	2.38, m		2.35, dd (13.7, 5.5)	
25	5.17, m	121.1	5.21, t (6.3)	120.9
26		135.5		135.7
27	1.72, s	26.4	1.72, s	26.4
28	1.61, s	18.4	1.62, s	18.4
29	1.45, m; 1.35, m	23.5	2.07, m; 1.97, m	27.7
30	1.44, m; 1.34, m	44.3	5.06, t (6.8)	125.5
31		71.6		132.3
32	1.12, s	29.27	1.65, s	25.1
33	1.12, s	29.29	1.57, s	18.4

^a^Assignments based on DEPT, HSQC, HMBC, and NOESY experiments; chemical shifts in ppm.
