# Corrigendum: UPLC-PDA-QTOFMS-guided isolation of prenylated xanthones and benzoylphloroglucinols from the leaves of *Garcinia oblongifolia* and their migration-inhibitory activity

**DOI:** 10.1038/srep39369

**Published:** 2016-12-23

**Authors:** Hong Zhang, Dan Zheng, Zhi-Jie Ding, Yuan-Zhi Lao, Hong-Sheng Tan, Hong-Xi Xu

Scientific Reports 6: Article number: 3578910.1038/srep35789; published online: 10 21 2016; updated: 12 23 2016

The original version of this Article contained a typographical error in the name of the author Dan Zheng, which was incorrectly given as Zheng Dan. This has now been corrected in both the PDF and HTML versions of the Article.

