# Supplementary material for: UPLC-PDA-QTOFMS-guided isolation of prenylated xanthones and benzoylphloroglucinols from the leaves of Garcinia oblongifolia and their migration-inhibitory activity
Source: Sci Rep. 2016 Oct 21;6:35789. doi: 10.1038/srep35789 (PMC5073302; doi:10.1038/srep35789)
Supplement: Supplementary Information [file srep35789-s1.pdf]

Supplementary information

UPLC-PDA-QTOFMS-guided isolation of prenylated xanthones and benzoylphloroglucinols from the leaves of *Garcinia oblongifolia* and their migration-inhibitory activity

Hong Zhang[1,2,a], Dan Zheng[1,a], Zhi-Jie Ding[1], Yuan-Zhi Lao[1,2], Hong-Sheng Tan[1,2], & Hong-Xi Xu[1,2,*]

[1] School of Pharmacy, Shanghai University of Traditional Chinese Medicine, Shanghai, 201203, P.R. China

[2] Engineering Research Centre of Shanghai Colleges for TCM New Drug Discovery, Shanghai, 201203, P.R. China

[a] These authors contributed equally to this work.

*Corresponding author. E-mail: xuhongxi88@gmail.com; Tel.: +86-21-51323089; Fax: +86-21-513230890

List of Supplementary information

Part 1 Computational details; The stable conformers of compounds 3−6 and 8；

Figure CS1. The stable conformers of (1*R*,5*R*,7*R*)-**3** calculated with DFT at the B3LYP/6-31G (d) level.

Figure CS2. The stable conformers of (1*S*,5*S*,7*S*)-**4** calculated with DFT at the B3LYP/6-31G (d) level.

Figure CS3. The stable conformers of (1*R*,5*S*,7*S*,24*R*)-**5** calculated with DFT at the B3LYP/6-31G (d) level.

Figure CS4. The stable conformers of (1*S*,5*S*,7*R*)-**6** calculated with DFT at the B3LYP/6-31G (d) level.

Figure CS5. The stable conformers of (1*R*,5*R*,7*S*)-**8** calculated with DFT at the B3LYP/6-31G (d) level.

Part 2 HRESIMS, IR, NMR, spectra of compounds 1–12; ECD spectra of compounds 3−8

Table S1 Cytotoxicity of the EtOAc-soluble fraction against TE1 cells

Table S2. Cytotoxicity of isolated compounds against cancer cell lines

Figure S1. UV, MS, and MS/MS spectra of **1** acquired by UPLC-PDA-QTOFMS analysis.

Figure S2. UV, MS, and MS/MS spectra of **2** acquired by UPLC-PDA-QTOFMS analysis.

Figure S3. UV, MS, and MS/MS spectra of **3** acquired by UPLC-PDA-QTOFMS analysis.

Figure S4. UV, MS, and MS/MS spectra of **4** acquired by UPLC-PDA-QTOFMS analysis.

Figure S5. UV, MS, and MS/MS spectra of **5** acquired by UPLC-PDA-QTOFMS analysis.

Figure S6. UV, MS, and MS/MS spectra of **6** acquired by UPLC-PDA-QTOFMS analysis.

Figure S7. UV, MS, and MS/MS spectra of **7** acquired by UPLC-PDA-QTOFMS analysis.

Figure S8. UV, MS, and MS/MS spectra of **8** acquired by UPLC-PDA-QTOFMS analysis.

Figure S9. UV, MS, and MS/MS spectra of **P10** acquired by UPLC-PDA-QTOFMS analysis.

Figure S10. UV, MS, and MS/MS spectra of **P11** acquired by UPLC-PDA-QTOFMS analysis.

Figure S11. UV, MS, and MS/MS spectra of **P12** acquired by UPLC-PDA-QTOFMS analysis.

Figure S12. UV, MS, and MS/MS spectra of **P13** acquired by UPLC-PDA-QTOFMS analysis.

Figure S13. UV, MS, and MS/MS spectra of **P14** acquired by UPLC-PDA-QTOFMS analysis.

Figure S14. Key correlations observed in the HMBC and NOESY NMR spectra of **5**.

Figure S15. Key correlations observed in the HMBC and NOESY NMR spectra of **6**.

Figure S16. Key correlations observed in the HMBC and NOESY NMR spectra of **7**.

Figure S17. Experimental ECD spectra of **7** and oblongifolin Q

Figure S18. MS/MS spectra of **8** and **9** acquired by UPLC-PDA-QTOFMS analysis.

Figure S19. Calculated ECD spectrum of **8** its experimental curve.

Figure S20. Anti-migration effect of the EtOAc-soluble fraction on human esophageal carcinoma cells (TE1) by wound healing assay.

Figure S21. The effect of compounds **3**–**8** on migration in TE1 cells measured by transwell assay.

Figure S22. The effect of compounds **1**, **2**, and **9** on cell invasion was measured by matrigel coated transwell assays.

Figure S23. The effect of compounds **1**, **2**, and **9** on B-RAF and C-RAF mRNA levels.

Oblongixanthone D (**1**)

Figure S24. HRESIMS spectrum of **1**

Figure S25. IR (KBr, disc) spectrum of **1**

Figure S26. ^{1}H NMR spectrum (DMSO-d_6, 600 MHz) of **1**

Figure S27. ^{13}C NMR spectrum (DMSO-d_6, 150 MHz) of **1**

Figure S28. DEPT NMR spectrum (DMSO-d_6, 150 MHz) of **1**

Figure S29. HSQC NMR spectrum (DMSO-d_6, 600 MHz, 150 MHz) of **1**

Figure S30. HMBC NMR spectrum (DMSO-d_6, 600 MHz, 150 MHz) of **1**

Oblongixanthone E (**2**)

Figure S31. ^{1}H NMR spectrum (DMSO-d_6, 400 MHz) of **2**

Figure S32. ^{13}C NMR spectrum (DMSO-d_6, 100 MHz) of **2**

Figure S33. DEPT NMR spectrum (DMSO-d_6, 100 MHz) of **2**

Figure S34. HSQC NMR spectrum (DMSO-d_6, 400 MHz, 100 MHz) of **2**

Figure S35. HMBC NMR spectrum (DMSO-d_6, 400 MHz, 100 MHz) of **2**

Oblongifolin V (**3**)

Figure S36. ^{1}H NMR spectrum (CD_3OD/0.1% TFA, 600 MHz) of **3**

Figure S37. ^{13}C NMR spectrum (CD_3OD/0.1% TFA, 150 MHz) of **3**

Figure S38. DEPT NMR spectrum (CD_3OD/0.1% TFA, 150 MHz) of **3**

Figure S39. HSQC NMR spectrum (CD_3OD/0.1% TFA, 600 MHz, 150 MHz) of **3**

Figure S40. HMBC NMR spectrum (CD_3OD/0.1% TFA, 600 MHz, 150 MHz) of **3**

Figure S41. NOSEY NMR spectrum (CD_3OD/0.1% TFA, 600 MHz) of **3**

Oblongifolin W (**4**)

Figure S42. ^{1}H NMR spectrum (CD_3OD/0.1% TFA, 600 MH$_{Z}$) of **4**

Figure S43. ^{13}C NMR spectrum (CD_3OD/0.1% TFA, 150 MH$_{Z}$) of **4**

Figure S44. DEPT NMR spectrum (CD_3OD/0.1% TFA, 150 MH$_{Z}$) of **4**

Figure S45. HSQC NMR spectrum (CD_3OD/0.1% TFA, 600 MH$_{Z}$, 150 MH$_{Z}$) of **4**

Figure S46. HMBC NMR spectrum (CD_3OD/0.1% TFA, 600 MH$_{Z}$, 150 MH$_{Z}$) of **4**

Figure S47. NOSEY NMR spectrum (CD_3OD/0.1% TFA, 600 MH$_{Z}$) of **4**

Oblongifolin X (**5**)

Figure S48. ^{1}H NMR spectrum (CD_3OD/0.1% TFA, 600 MH$_{Z}$) of **5**

Figure S49. ^{13}C NMR spectrum (CD_3OD/0.1% TFA, 150 MH$_{Z}$) of **5**

Figure S50. DEPT NMR spectrum (CD_3OD/0.1% TFA, 150 MH$_{Z}$) of **5**

Figure S51. HSQC NMR spectrum (CD_3OD/0.1% TFA, 600 MH$_{Z}$, 150 MH$_{Z}$) of **5**

Figure S52. HMBC NMR spectrum (CD_3OD/0.1% TFA, 600 MH$_{Z}$, 150 MH$_{Z}$) of **5**

Figure S53. NOSEY NMR spectrum (CD_3OD/0.1% TFA, 600 MH$_{Z}$) of **5**

Oblongifolin Y (**6**)

Figure S54. ^{1}H NMR spectrum (Acetone-d_6, 600 MH$_{Z}$) of **6**

Figure S55. ^{13}C NMR spectrum (Acetone-d_6, 150 MH$_{Z}$) of **6**

Figure S56. DEPT NMR spectrum (Acetone-d_6, 150 MH$_{Z}$) of **6**

Figure S57. HSQC NMR spectrum (Acetone-d_6, 600 MH$_{Z}$, 150 MH$_{Z}$) of **6**

Figure S58. HMBC NMR spectrum (Acetone-d_6, 600 MH$_{Z}$, 150 MH$_{Z}$) of **6**

Figure S59. NOSEY NMR spectrum (Acetone-d_6, 600 MH$_{Z}$) of **6**

Oblongifolin Z (**7**)

Figure S60. ^{1}H NMR spectrum (CD_3OD/0.1% TFA, 400 MHz) of **7**

Figure S61. ^{13}C NMR spectrum (CD_3OD/0.1% TFA, 100 MHz) of **7**

Figure S62. DEPT NMR spectrum (CD_3OD/0.1% TFA, 100 MHz) of **7**

Figure S63. HSQC NMR spectrum (CD_3OD/0.1% TFA, 400 MHz, 100 MHz) of **7**

Figure S64. HMBC NMR spectrum (CD_3OD/0.1% TFA, 400 MHz, 100 MHz) of **7**

Figure S65. NOSEY NMR spectrum (CD_3OD/0.1% TFA, 400 MHz) of **7**

Oblongifolin AA (**8**)

Figure S66. ^{1}H NMR spectrum (CD_3OD/0.1% TFA, 400 MHz) of **8**

Figure S67. ^{13}C NMR spectrum (CD_3OD/0.1% TFA, 100 MHz) of **8**

Figure S68. DEPT NMR spectrum (CD_3OD/0.1% TFA, 100 MHz) of **8**

Figure S69. HSQC NMR spectrum (CD_3OD/0.1% TFA, 400 MHz, 100 MHz) of **8**

Figure S70. HMBC NMR spectrum (CD_3OD/0.1% TFA, 400 MHz, 100 MHz) of **8**

Figure S71. NOSEY NMR spectrum (CD_3OD/0.1% TFA, 400 MHz) of **8**

Supplementary information Available

Part 1. Computational details

The theoretical calculations of compounds **3**–**6** and **8** were performed using Gaussian 09.[1] Conformational analysis was initially carried out using Accelrys Discovery Studio 2.5 to generate conformations by Best, then minimize them by Smart Minimizer using the CHARMm molecular mechanics force field. The minimized conformers were further optimized at the B3LYP/6-31G (d) level in the gas phase. Room-temperature equilibrium populations were calculated according to the Boltzmann distribution law. The theoretical calculation of ECD was performed using TDDFT at the B3LYP/6-31G (d, p) level in the gas phase. The ECD spectra of **3**–**6** and **8** were obtained by weighing the Boltzmann distribution rate of each geometric conformation. SpecDis 1.61[2] was used to sum up single CD spectra after a Boltzmann statistical weighting, for the gauss curve generation and for the comparison with experimental data.

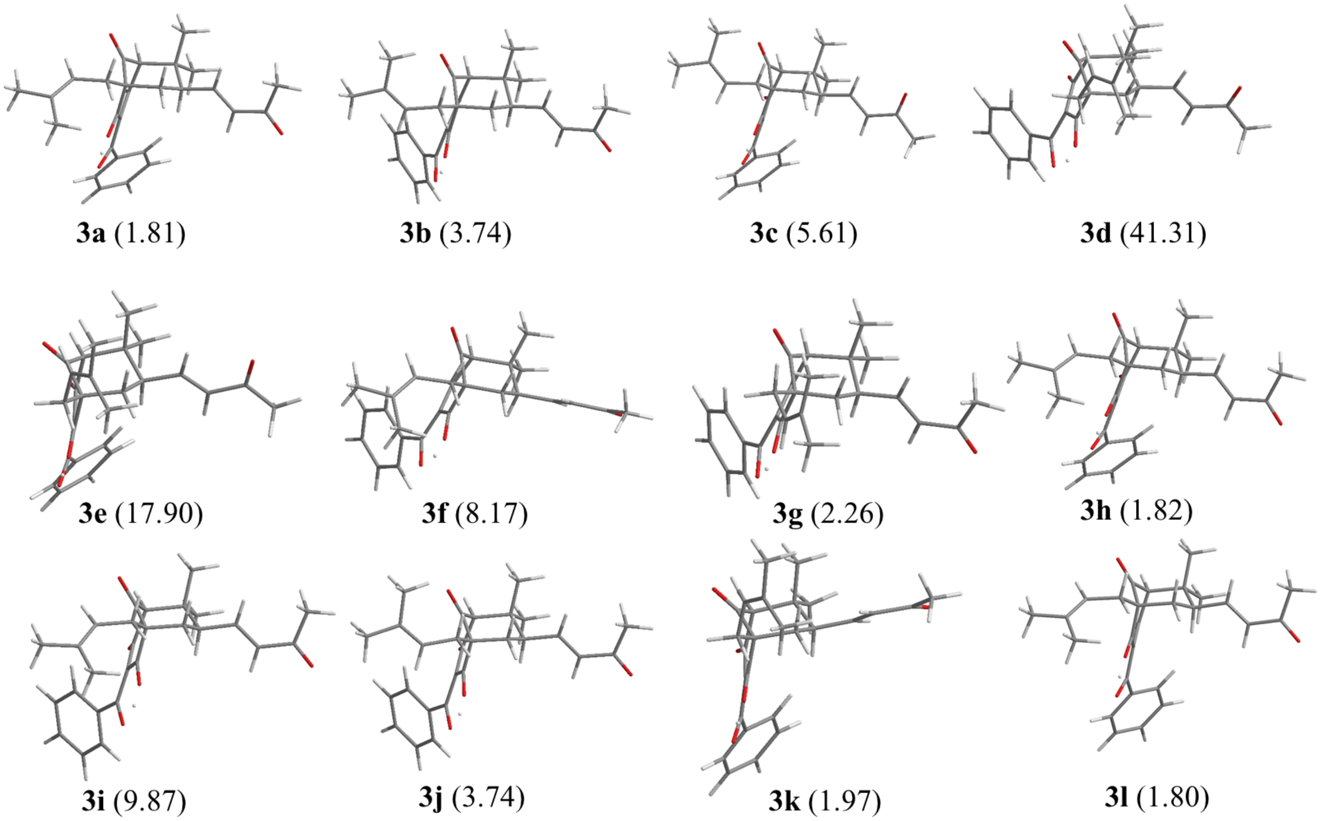

Figure CS1. The stable conformers of (1*R*,5*R*,7*R*)-**3** calculated with DFT at the B3LYP/6-31G (d) level. Relative populations are in parentheses. Equilibrium Populations calculated by the relative free Gibbs energies at B3LYP/6-31G (d) level in the gas phase, assuming Boltzman statistics at T = 298.15 K and 1 atm.

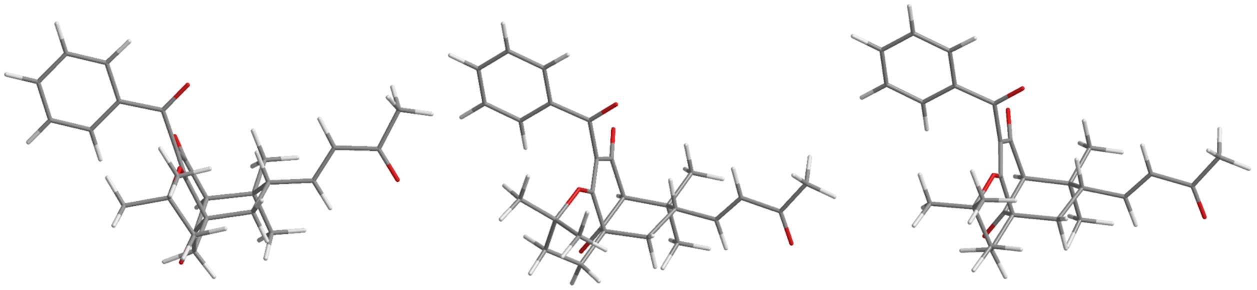

4a (48.41) **4b** (3.17) **4c** (48.41)

Figure CS2. The stable conformers of (1*S*,5*S*,7*S*)-**4** calculated with DFT at the B3LYP/6-31G (d) level. Relative populations are in parentheses. Equilibrium Populations calculated by the relative free Gibbs energies at B3LYP/6-31G (d) level in the gas phase, assuming Boltzman statistics at T = 298.15 K and 1 atm.

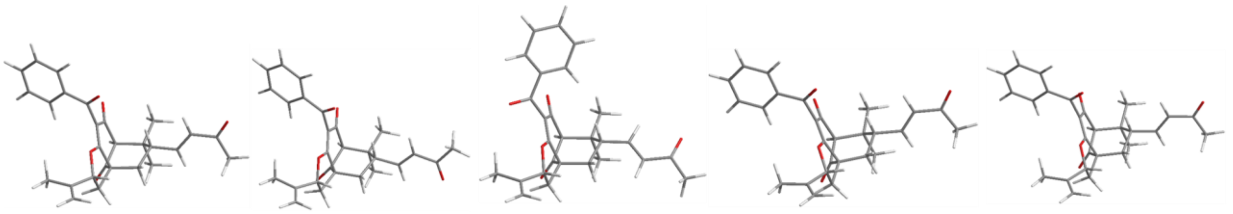

4a (4.12) **4b** (52.10) **4c** (1.16) **4d** (4.12) **4e** (4.13)

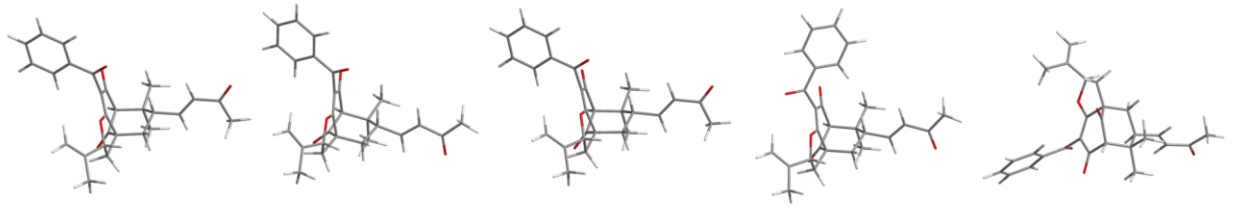

4f (1.73) **4g** (23.47) **4h** (1.71) **4i** (3.34) **4a** (4.12)

Figure CS3. The stable conformers of (1*R*,5*S*,7*S*,24*R*)-**5** calculated with DFT at the B3LYP/6-31G (d) level. Relative populations are in parentheses. Equilibrium Populations calculated by the relative free Gibbs energies at B3LYP/6-31G (d) level in the gas phase, assuming Boltzman statistics at T = 298.15 K and 1 atm.

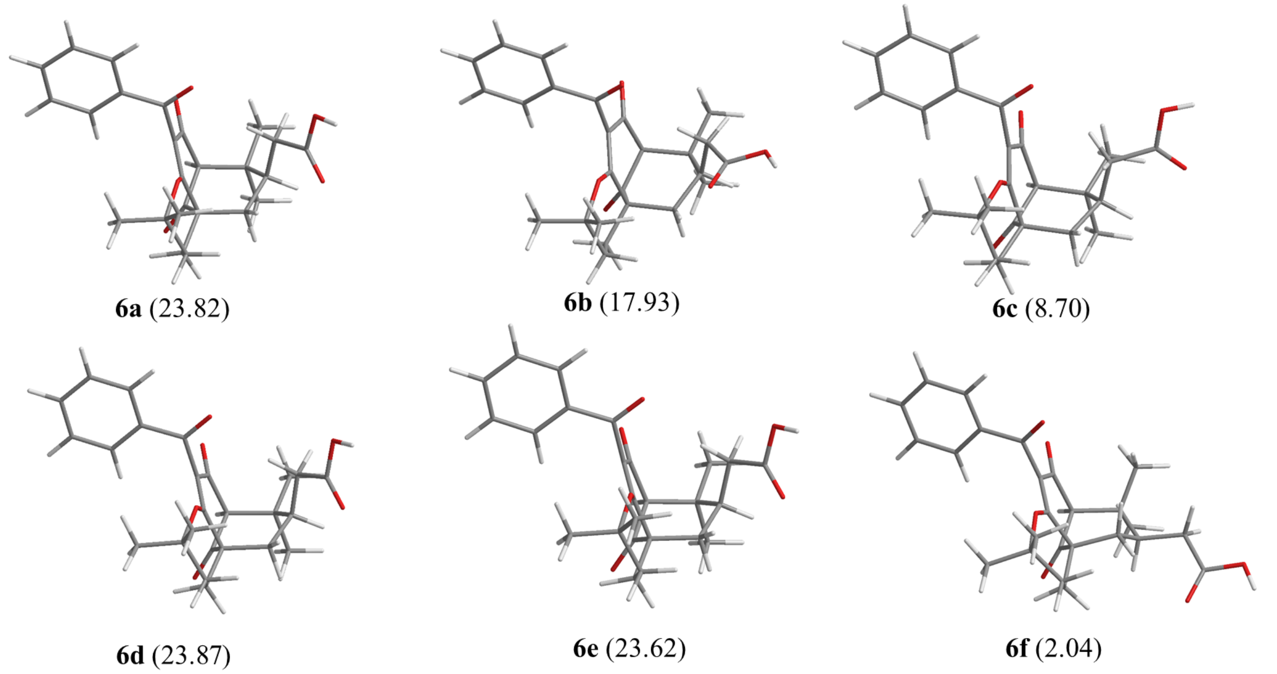

Figure CS4. The stable conformers of (1*S*,5*S*,7*R*)-**6** calculated with DFT at the B3LYP/6-31G (d) level. Relative populations are in parentheses. Equilibrium Populations calculated by the relative free Gibbs energies at B3LYP/6-31G (d) level in the gas phase, assuming Boltzman statistics at T = 298.15 K and 1 atm.

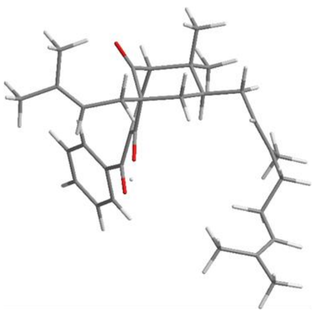

8a (21.48)

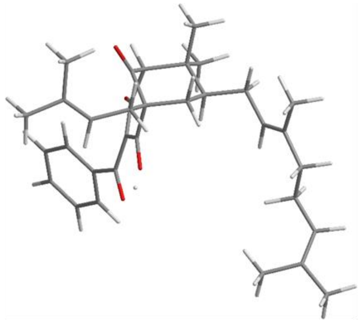

8b (68.78)

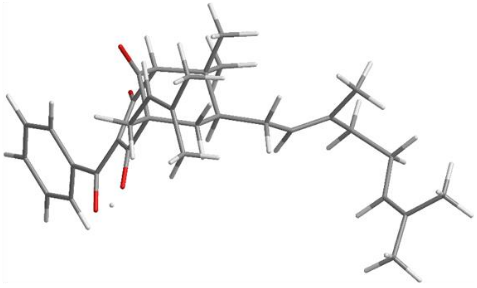

8c (2.13)

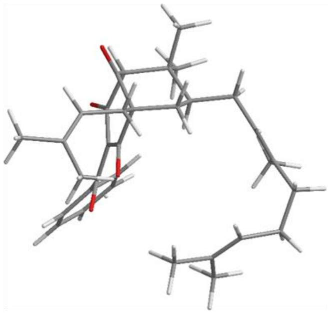

8d (4.01)

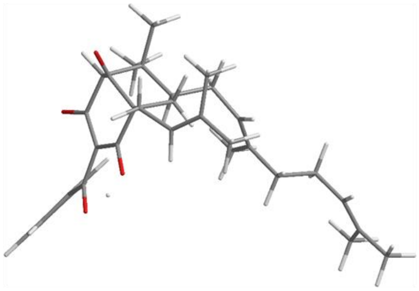

8e (3.60)

Figure CS5. The stable conformers of (1*R*,5*R*,7*S*)-**8** calculated with DFT at the B3LYP/6-31G (d) level. Relative populations are in parentheses. Equilibrium Populations calculated by the relative free Gibbs energies at B3LYP/6-31G (d) level in the gas phase, assuming Boltzman statistics at T = 298.15 K and 1 atm.

References:

(1) Gaussian 09, Revision D.01, M. J. Frisch, G. W. Trucks, H. B. Schlegel, G. E. Scuseria, M. A. Robb, J. R. Cheeseman, G. Scalmani, V. Barone, B. Mennucci, G. A. Petersson, H. Nakatsuji, M. Caricato, X. Li, H. P. Hratchian, A. F. Izmaylov, J. Bloino, G. Zheng, J. L. Sonnenberg, M. Hada, M. Ehara, K. Toyota, R. Fukuda, J. Hasegawa, M. Ishida, T. Nakajima, Y. Honda, O. Kitao, H. Nakai, T. Vreven, J. A. Montgomery, Jr., J. E. Peralta, F. Ogliaro, M. Bearpark, J. J. Heyd, E. Brothers, K. N. Kudin, V. N. Staroverov, T. Keith, R. Kobayashi, J. Normand, K. Raghavachari, A. Rendell, J. C. Burant, S. S. Iyengar, J. Tomasi, M. Cossi, N. Rega, J. M. Millam, M. Klene, J. E. Knox, J. B. Cross, V. Bakken, C. Adamo, J. Jaramillo, R. Gomperts, R. E. Stratmann, O. Yazyev, A. J. Austin, R. Cammi, C. Pomelli, J. W. Ochterski, R. L. Martin, K. Morokuma, V. G. Zakrzewski, G. A. Voth, P. Salvador, J. J. Dannenberg, S. Dapprich, A. D. Daniels, O. Farkas, J. B. Foresman,

J. V. Ortiz, J. Cioslowski, and D. J. Fox, Gaussian, Inc., Wallingford CT, 2013.
(2) T. Bruhn, A. Schaumlöffel, Y. Hemberger, G. Bringmann, SpecDis version 1.64, University of Wuerzburg, Germany, 2015.

Part 2 Results; HRESIMS, IR, NMR, spectra of compounds **1–12;** ECD spectra of compounds **3–8**

Table S1 Cytotoxicity of the EtOAc-soluble fraction against TE1 cells.

Samples	IC_{50}
Fraction	68.3 ± 8.1 μg/ml
Sorafenib	17.0 ± 3.0 μM

Table S2 Cytotoxicity of isolated compounds against cancer cell lines[a]

Compounds	IC_{50} (μM)				
	TE1	KYSE150	HepG2	A549	HL7702
**1**	15.4 ± 4.8	38.6 ± 2.3	> 100	28.3 ± 0.5	64.2 ± 3.8
**2**	19.7 ± 3.6	39.0 ± 4.7	> 100	> 100	88.5 ± 2.9
**3**	> 100	> 100	> 100	> 100	> 100
**4**	> 100	> 100	> 100	> 100	> 100
**5**	> 100	> 100	> 100	> 100	> 100
**6**	> 100	> 100	> 100	> 100	> 100
**7**	> 100	> 100	> 100	> 100	> 100
**8**	> 100	> 100	> 100	> 100	> 100
**9**	80.2 ± 10.4	43.6 ± 1.6	>100	>100	40.8 ± 5.3
**SFB**[b]	22.2 ± 1.63	< 6.25	12.9 ± 0.4	18.9 ± 2.3	< 6.25

[a]Results are expressed as IC_{50} (mean values ± SD, n = 3) in μM. [b]Positive control: sorafenib. [c]Human normal hepatic cells.

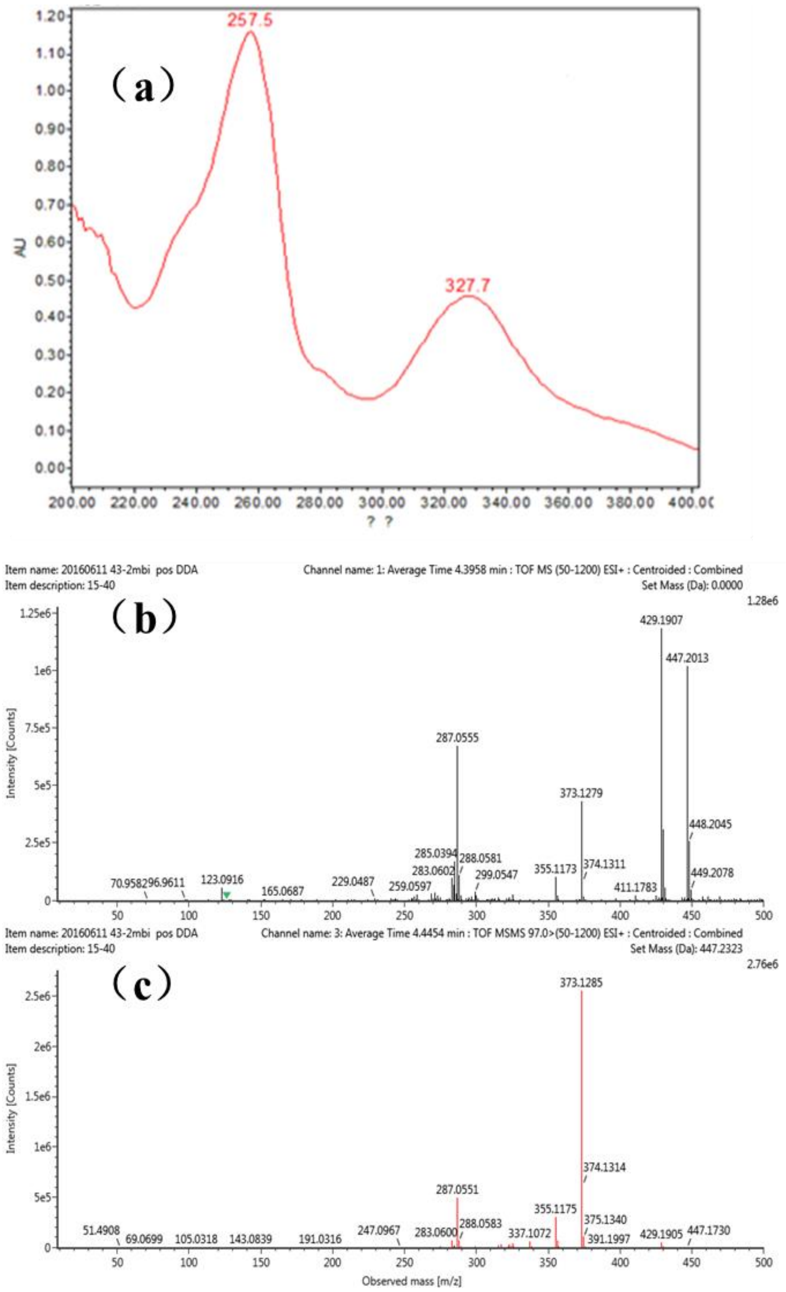

Figure S1. UV, MS, and MS/MS spectra of **1** acquired by UPLC-PDA-QTOFMS analysis. (a) UV spectrum of compound **1**; (b) MS spectrum of **1** in positive mode; (c) MS/MS spectrum of **1** for selected $[M + H]^+$ at m/z 447 (ramping collision energy 15–40 V).

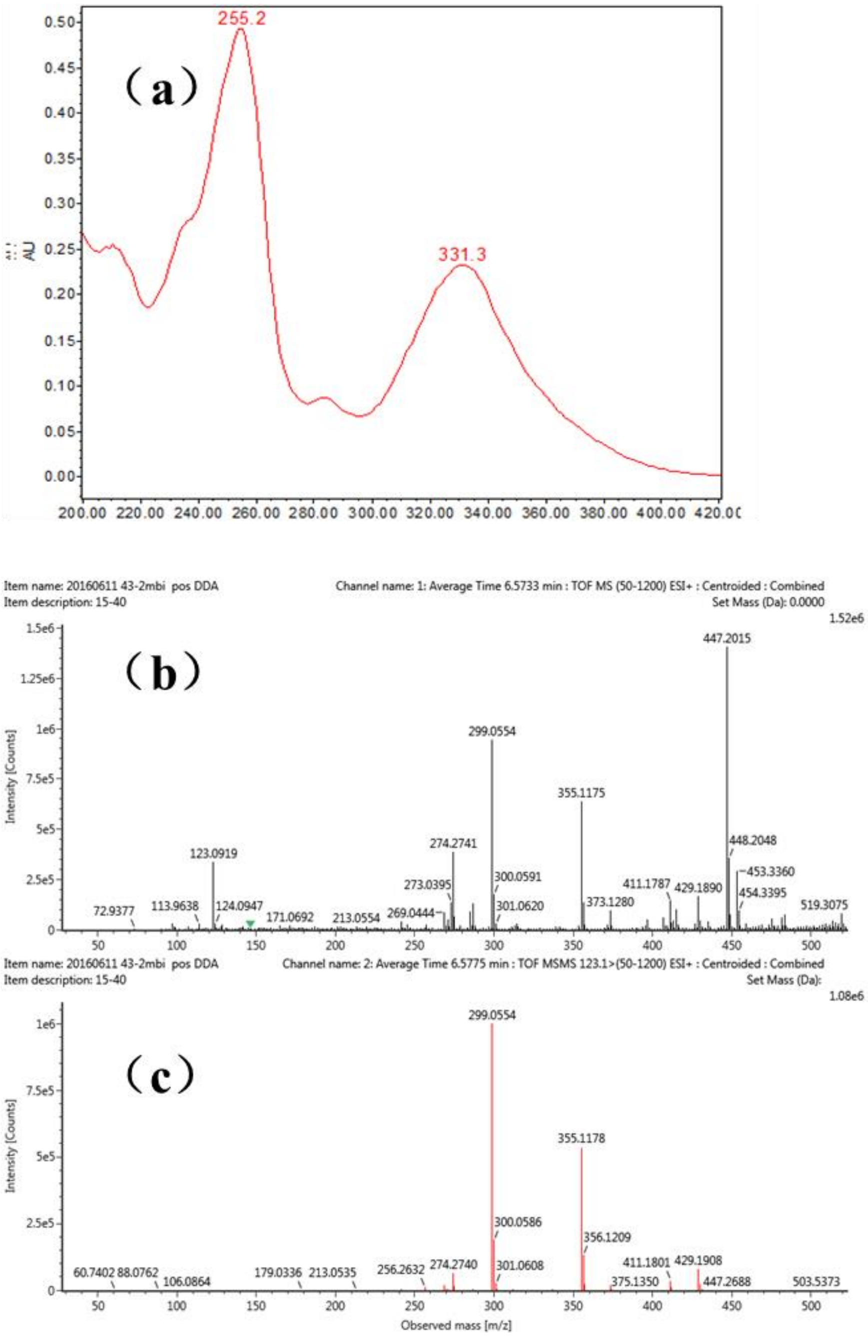

Figure S2. UV, MS, and MS/MS spectra of **2** acquired by UPLC-PDA-QTOFMS analysis. (a) UV spectrum of compound **2**; (b) MS spectrum of **2** in positive mode; (c) MS/MS spectrum of **2** for selected $[M + H]^+$ at m/z 447 (ramping collision energy 15–40 V).

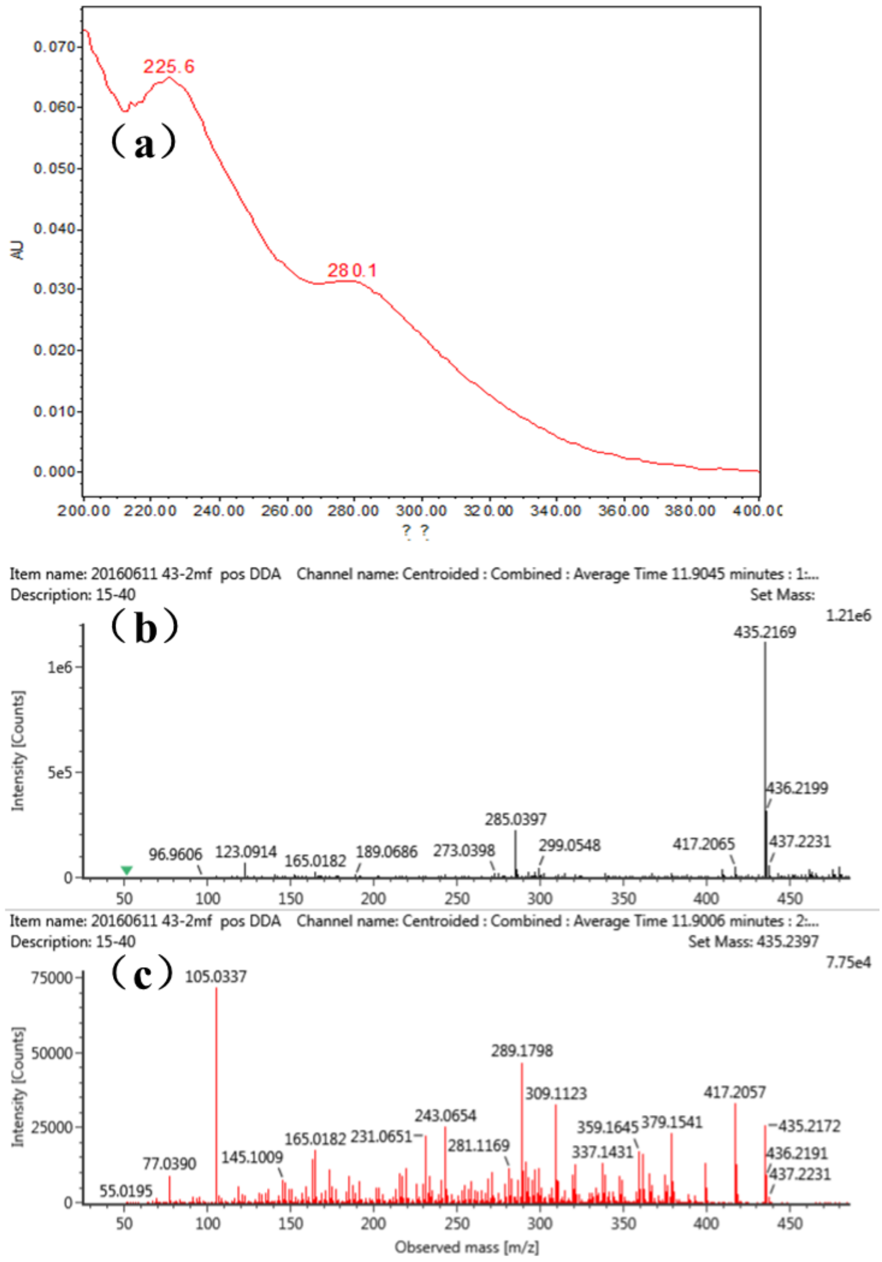

Figure S3. UV, MS, and MS/MS spectra of **3** acquired by UPLC-PDA-QTOFMS analysis. (a) UV spectrum of compound **3**; (b) MS spectrum of **3** in positive mode; (c) MS/MS spectrum of **3** for selected $[M + H]^+$ at *m*/*z* 435 (ramping collision energy 15–40 V).

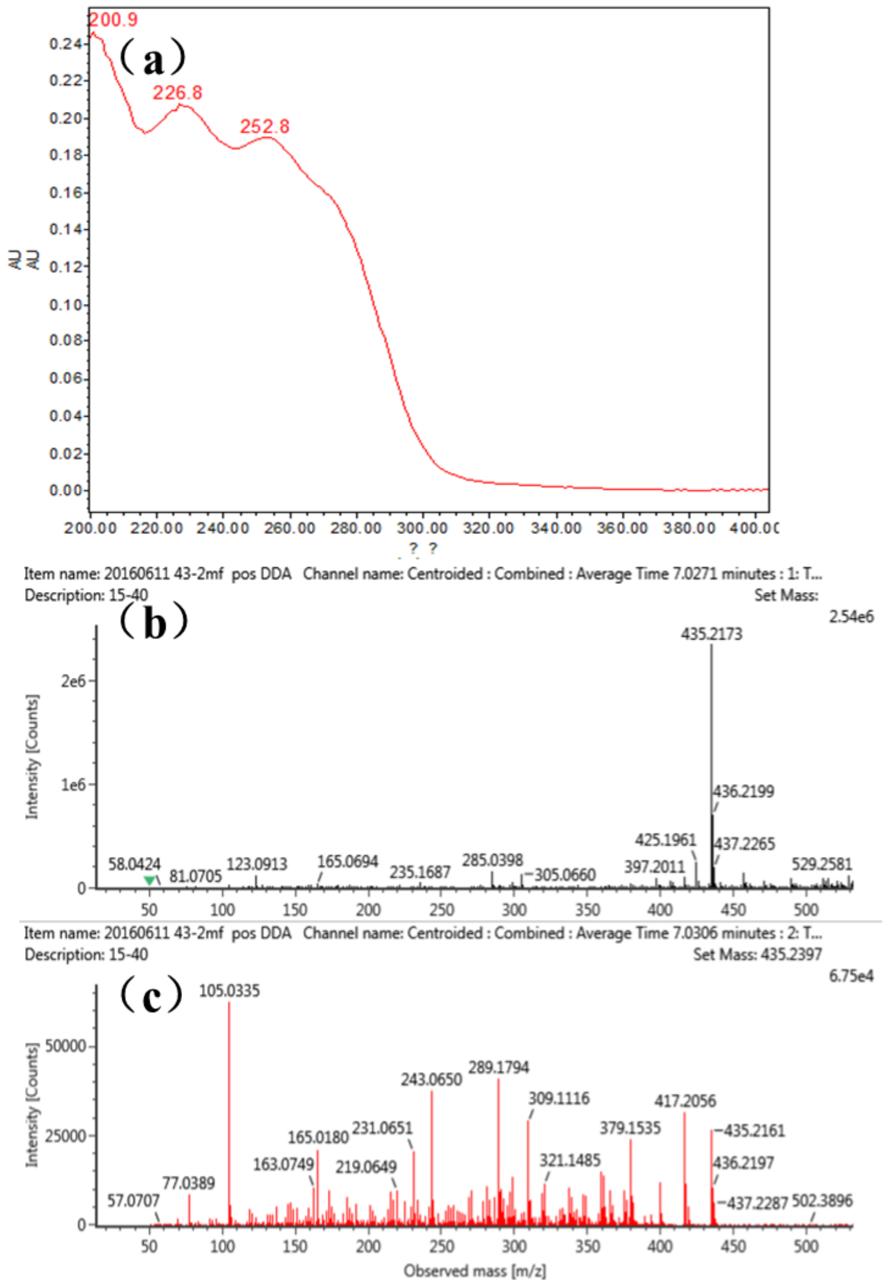

Figure S4. UV, MS, and MS/MS spectra of **4** acquired by UPLC-PDA-QTOFMS analysis. (a) UV spectrum of compound **4**; (b) MS spectrum of **4** in positive mode; (c) MS/MS spectrum of **4** for selected $[M + H]^+$ at *m*/*z* 435 (ramping collision energy 15–40 V).

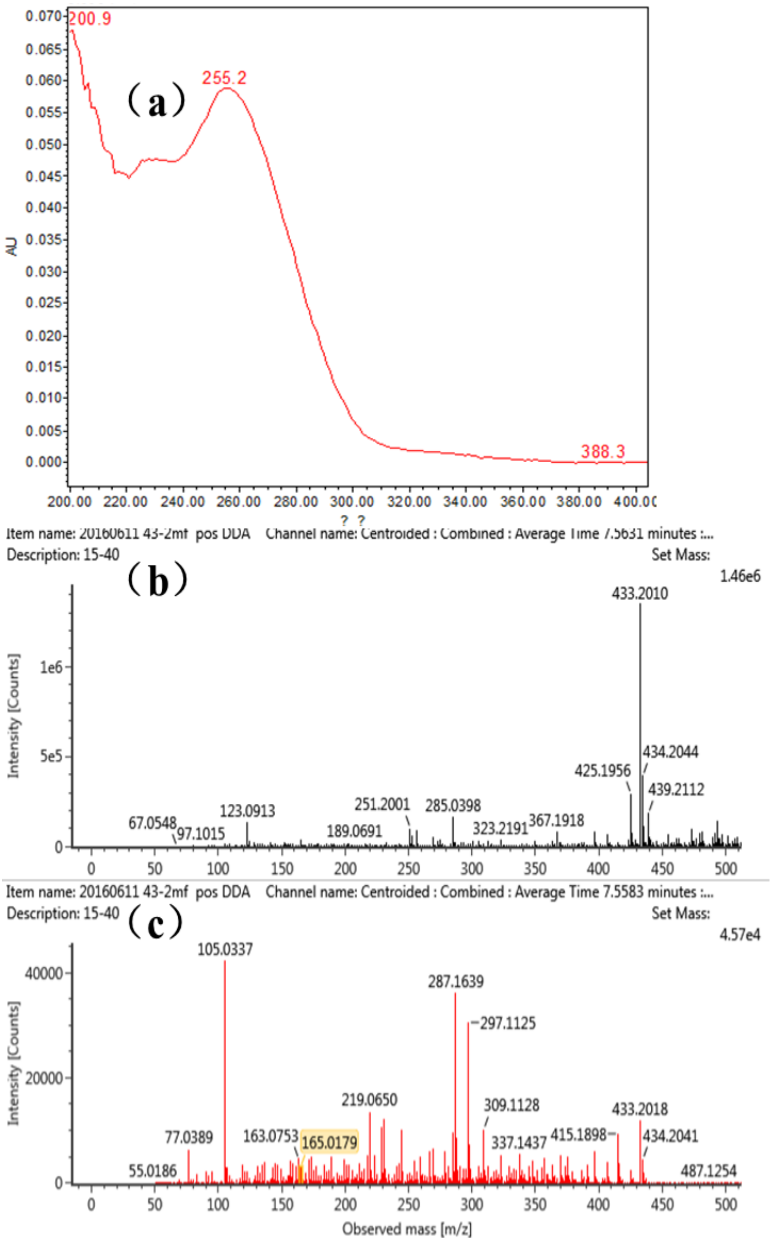

Figure S5. UV, MS, and MS/MS spectra of **5** acquired by UPLC-PDA-QTOFMS analysis. (a) UV spectrum of compound **5**; (b) MS spectrum of **5** in positive mode; (c) MS/MS spectrum of **5** for selected $[M + H]^+$ at *m*/*z* 433 (ramping collision energy 15–40 V).

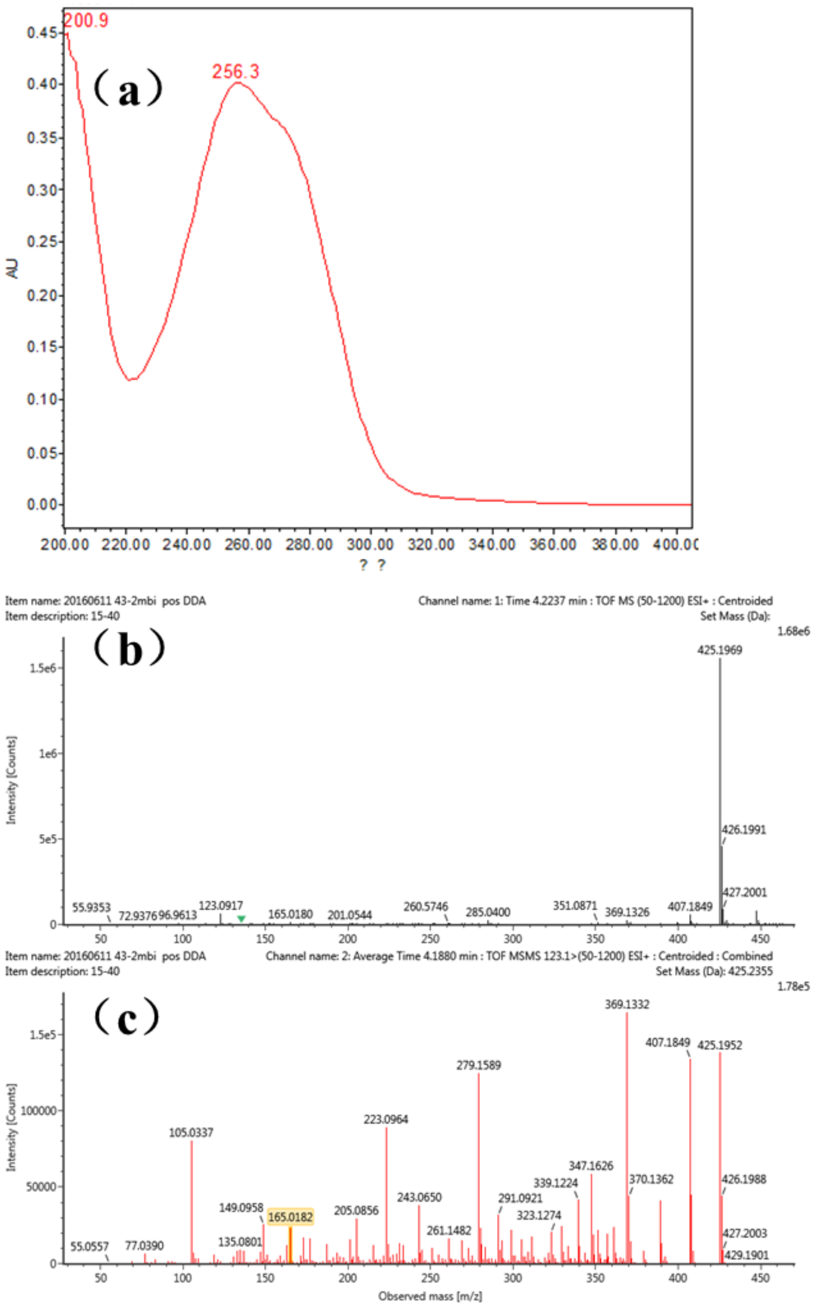

Figure S6. UV, MS, and MS/MS spectra of **6** acquired by UPLC-PDA-QTOFMS analysis. (a) UV spectrum of compound **6**; (b) MS spectrum of **6** in positive mode; (c) MS/MS spectrum of **6** for selected $[M + H]^+$ at m/z 433 (ramping collision energy 15–40 V).

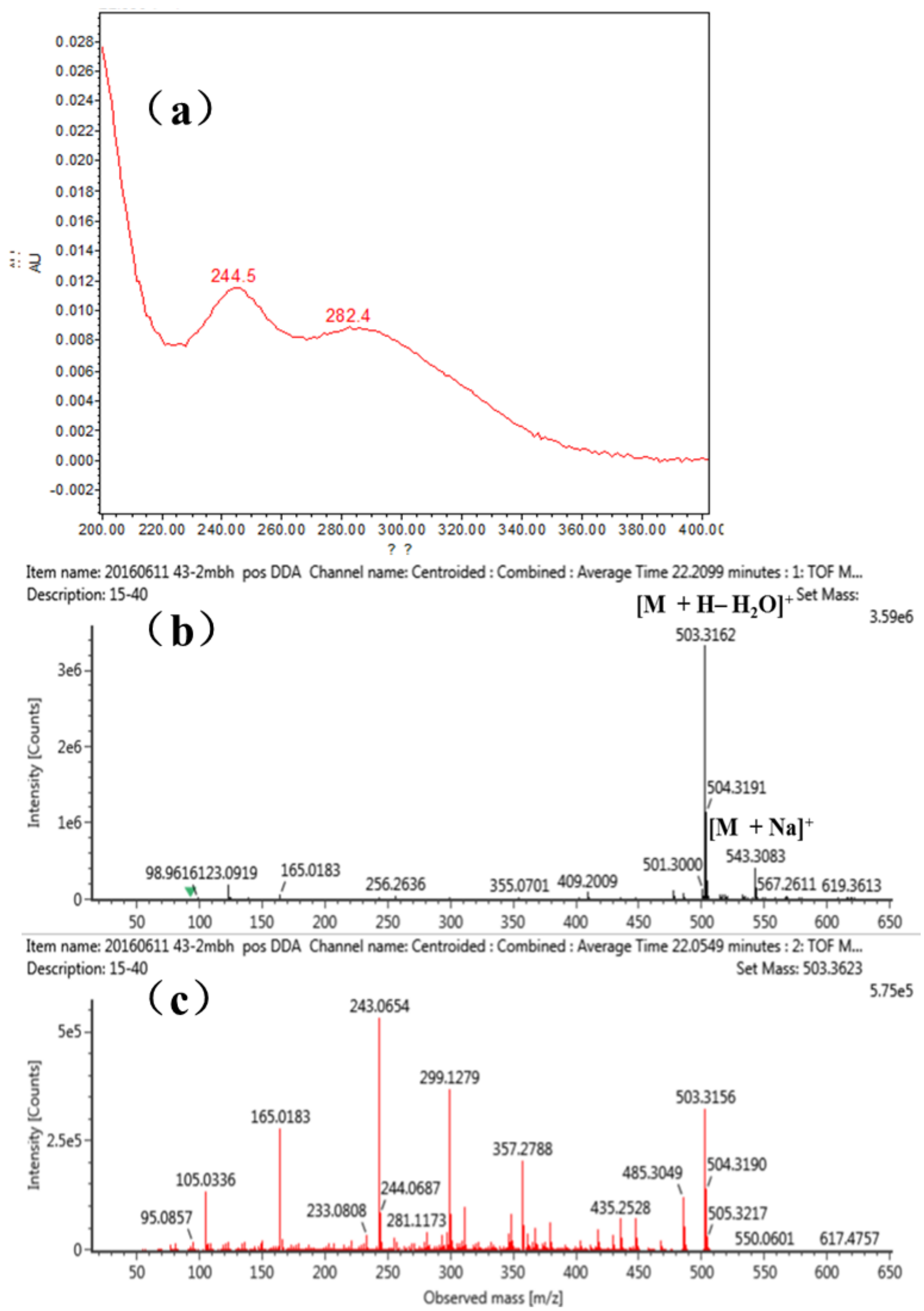

Figure S7. UV, MS, and MS/MS spectra of **7** acquired by UPLC-PDA-QTOFMS analysis. (a) UV spectrum of **7**; (b) MS spectrum of **7** in positive mode; (c) MS/MS spectrum of **7** for selected $[M + H]^+$ at m/z 503 (ramping collision energy 15–40 V).

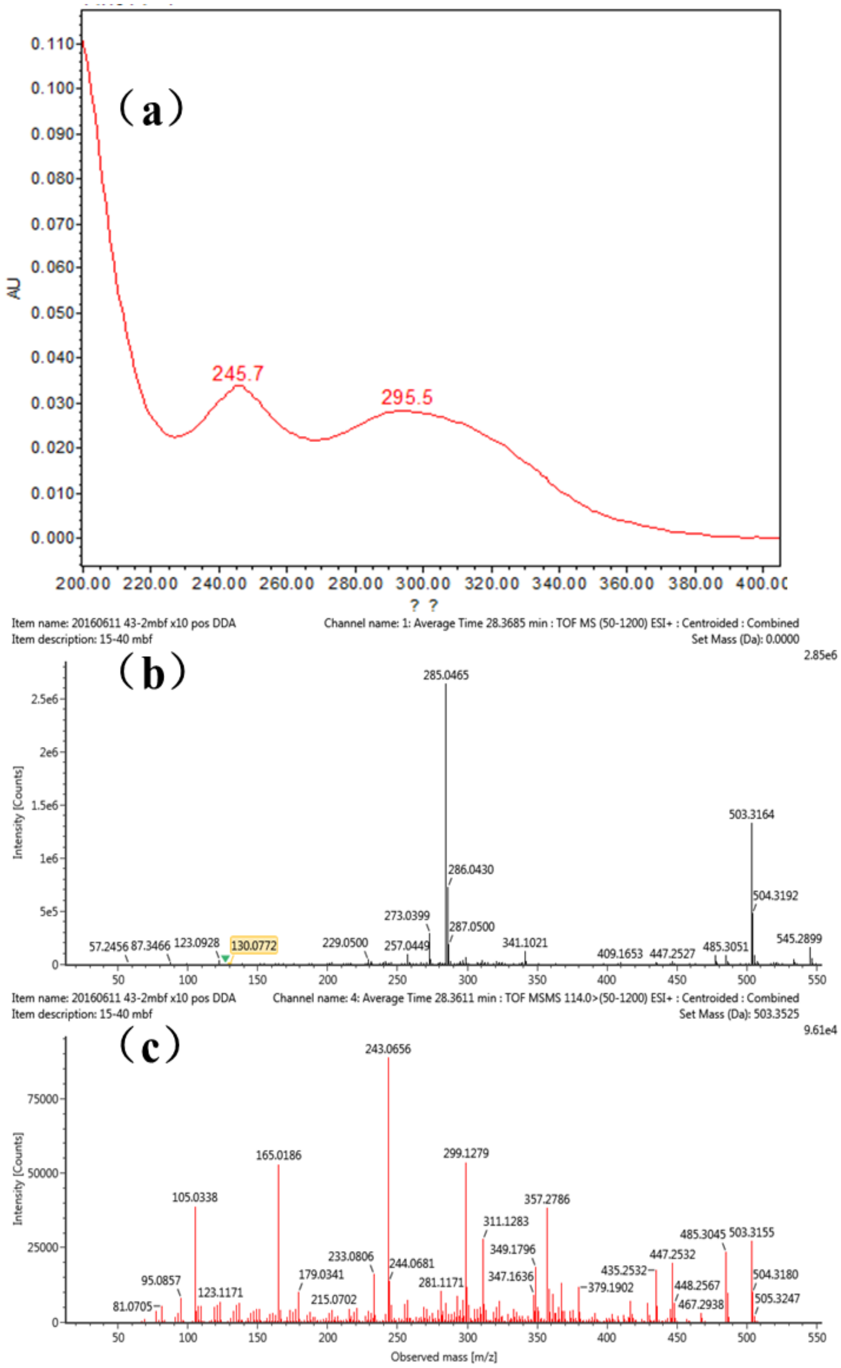

Figure S8. UV, MS, and MS/MS spectra of **8** acquired by UPLC-PDA-QTOFMS analysis. (a) UV spectrum of **8**; (b) MS spectrum of **8** in positive mode; (c) MS/MS spectrum of **8** for selected $[M + H]^+$ at m/z 503 (ramping collision energy 15–40 V).

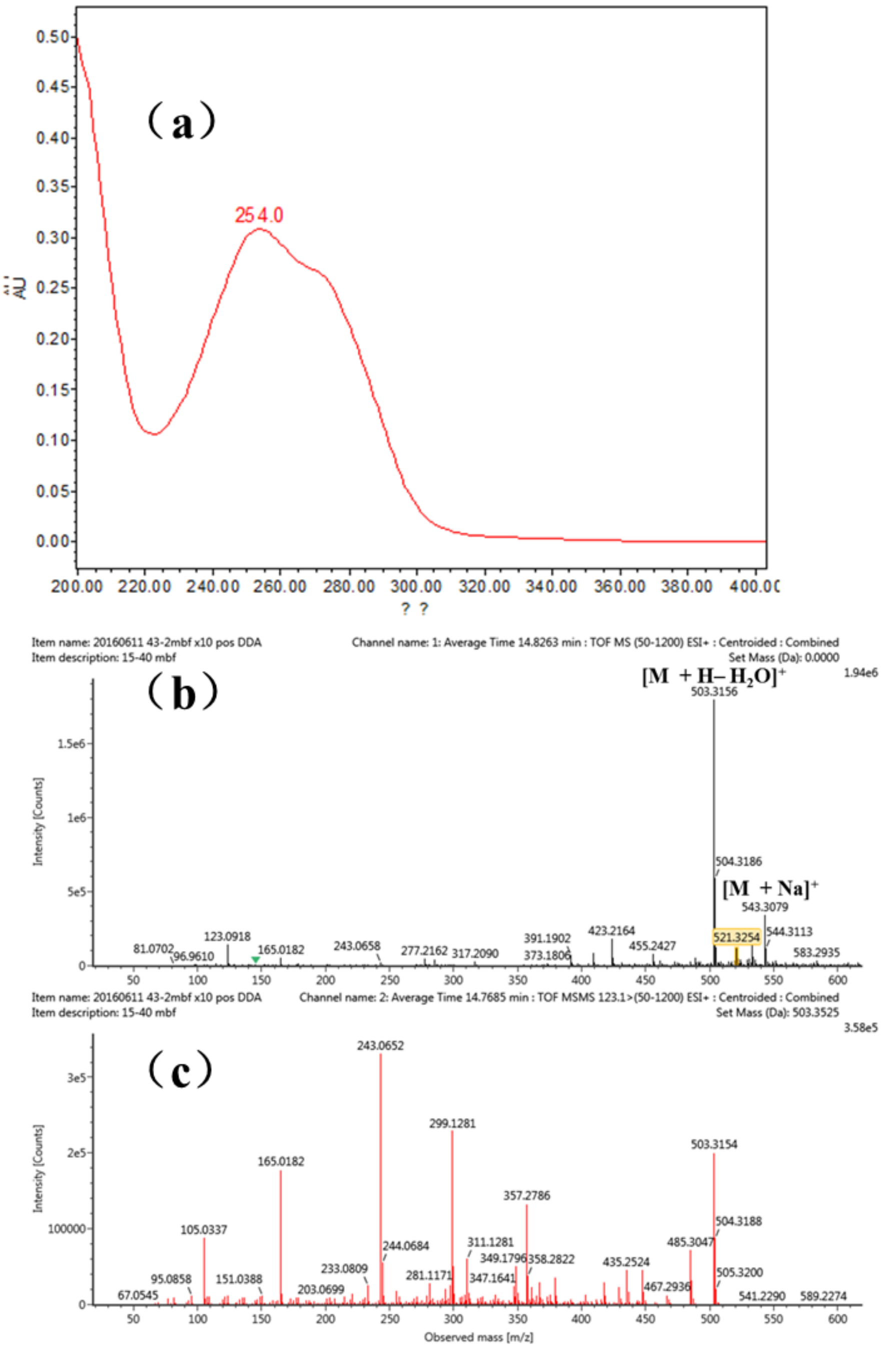

Figure S9. UV, MS, and MS/MS spectra of **P10** acquired by UPLC-PDA-QTOFMS analysis. (a) UV spectrum of **P10**; (b) MS spectrum of **P10** in positive mode; (c) MS/MS spectrum of **P10** for selected $[M + H – H_2O]^+$ at *m*/*z* 503 (ramping collision energy 15–40 V).

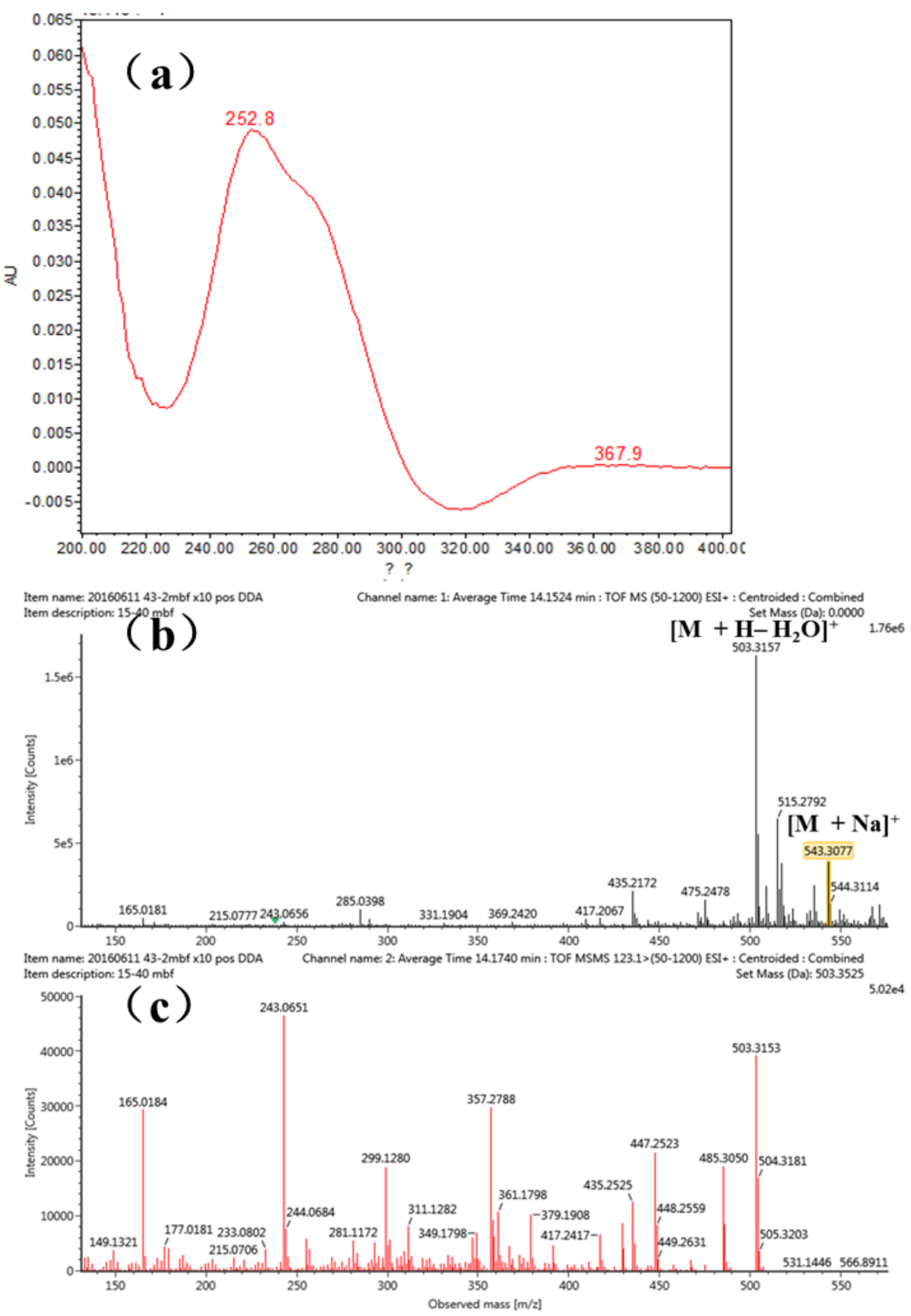

Figure S10. UV, MS, and MS/MS spectra of **P11** acquired by UPLC-PDA-QTOFMS analysis. (a) UV spectrum of **P11**; (b) MS spectrum of **P11** in positive mode; (c) MS/MS spectrum of **P11** for selected $[M + H - H_2O]^+$ at *m*/*z* 503 (ramping collision energy 15–40 V).

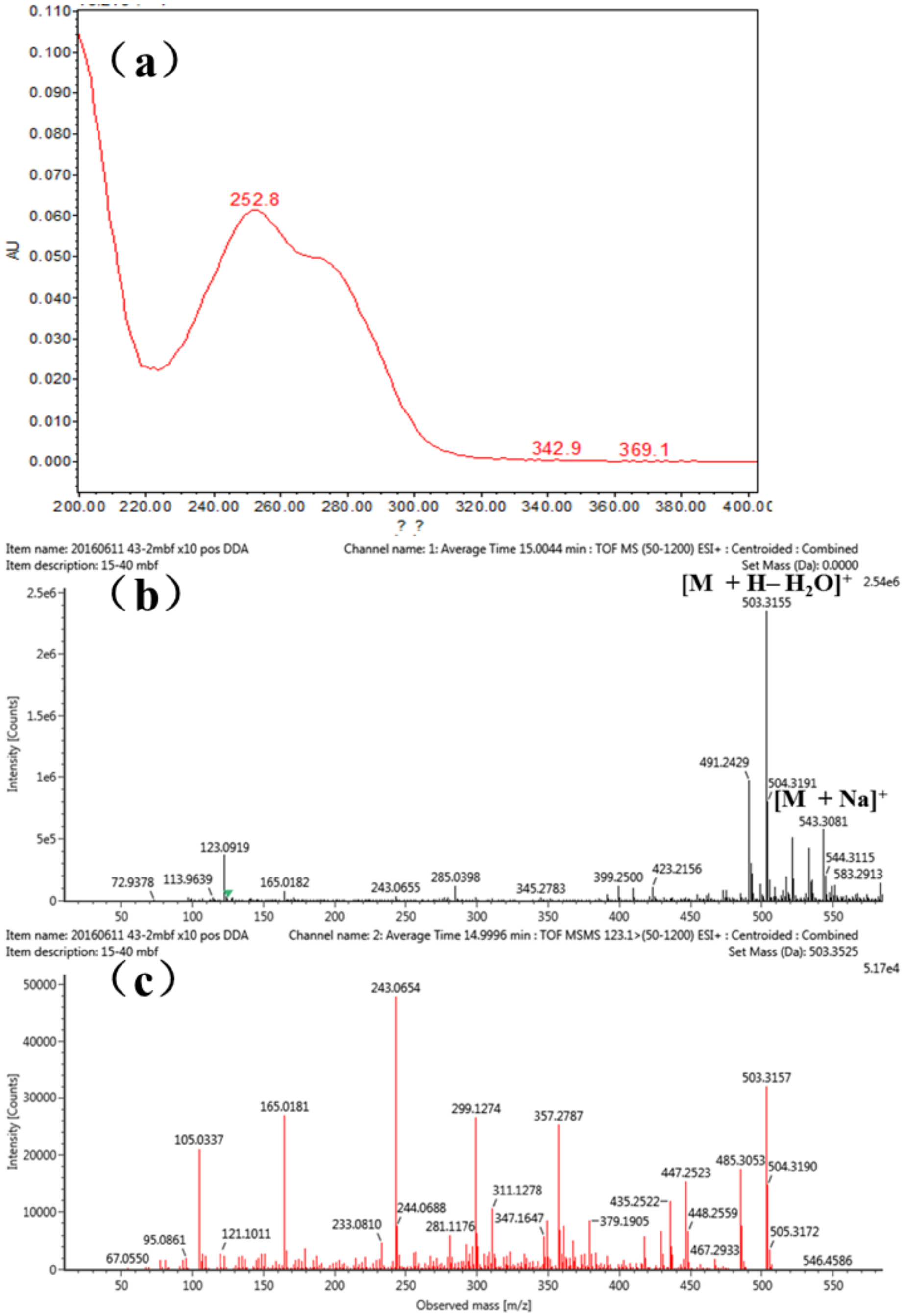

Figure S11. UV, MS, and MS/MS spectra of **P12** acquired by UPLC-PDA-QTOFMS analysis. (a) UV spectrum of **P12**; (b) MS spectrum of **P12** in positive mode; (c) MS/MS spectrum of **P12** for selected $[M + H - H_2O]^+$ at *m*/*z* 503 (ramping collision energy 15–40 V).

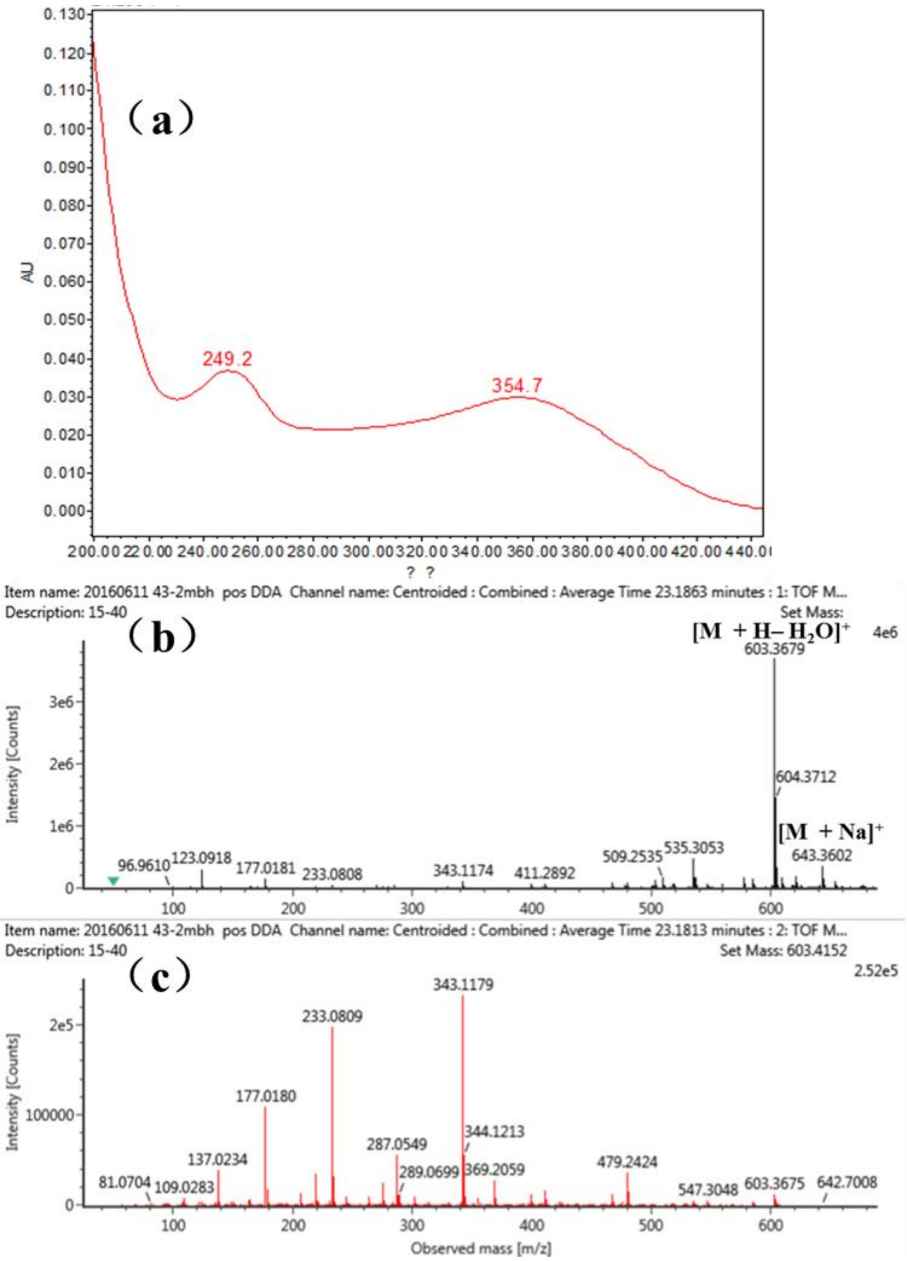

Figure S12. UV, MS, and MS/MS spectra of **P13** acquired by UPLC-PDA-QTOFMS analysis. (a) UV spectrum of **P13**; (b) MS spectrum of **P13** in positive mode; (c) MS/MS spectrum of **P13** for selected $[M + H - H_2O]^+$ at *m/z* 603 (ramping collision energy 15–40 V).

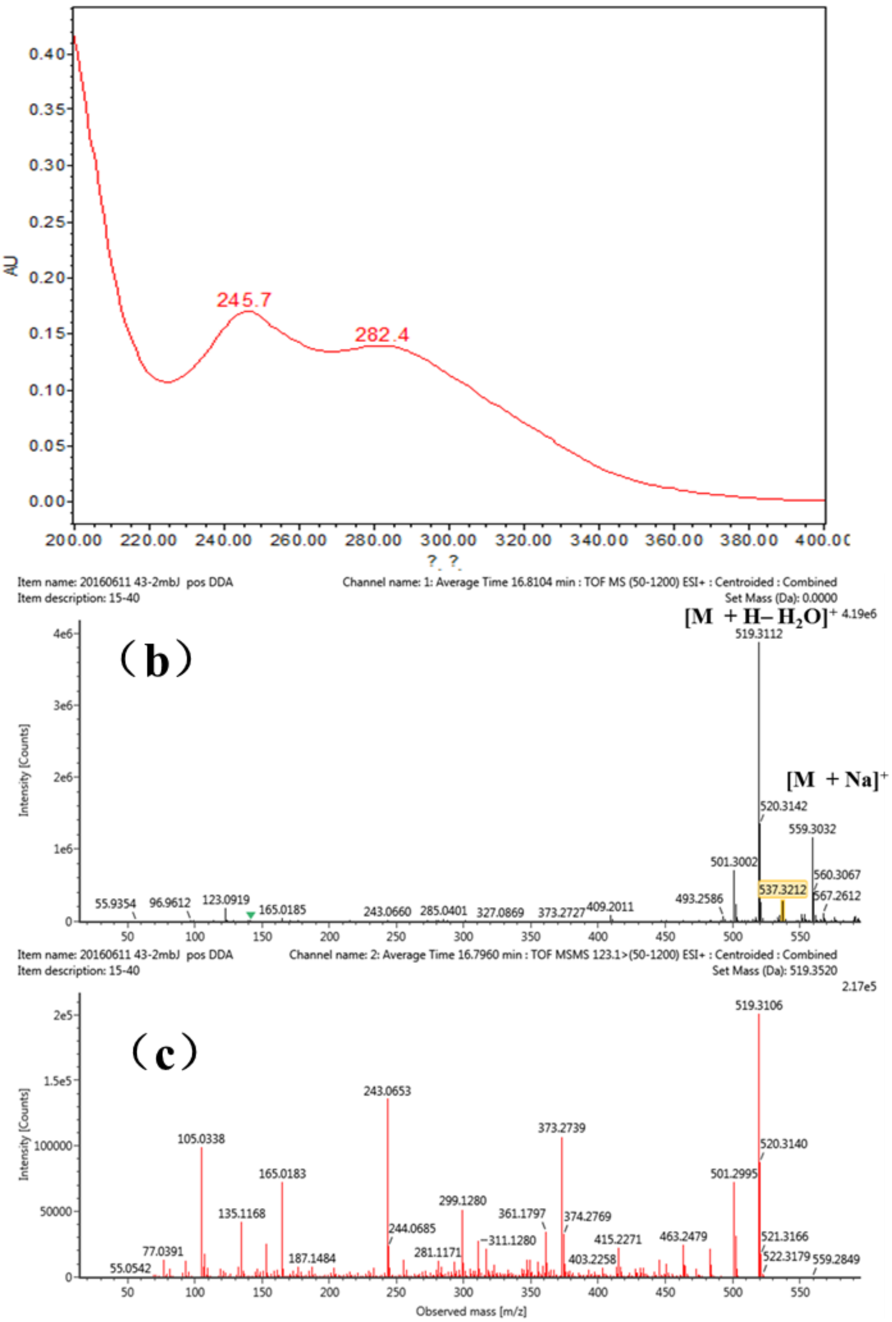

Figure S13. UV, MS, and MS/MS spectra of **P14** acquired by UPLC-PDA-QTOFMS analysis. (a) UV spectrum of **P14**; (b) MS spectrum of **P14** in positive mode; (c) MS/MS spectrum of **P14** for selected $[M + H – 2H_2O]^+$ at m/z 519 (ramping collision energy 15–40 V).

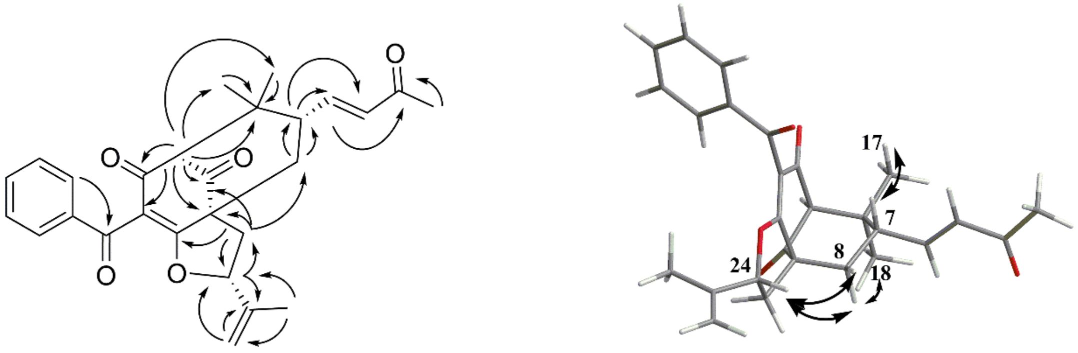

Figure S14. Key correlations observed in the HMBC and NOESY NMR spectra of **5**.

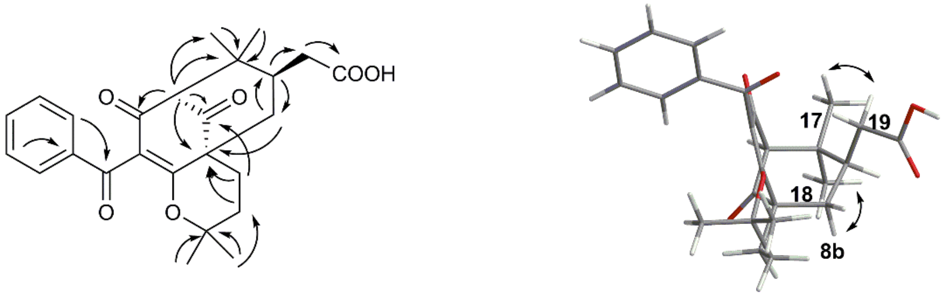

Figure S15. Key correlations observed in the HMBC and NOESY NMR spectra of **6**.

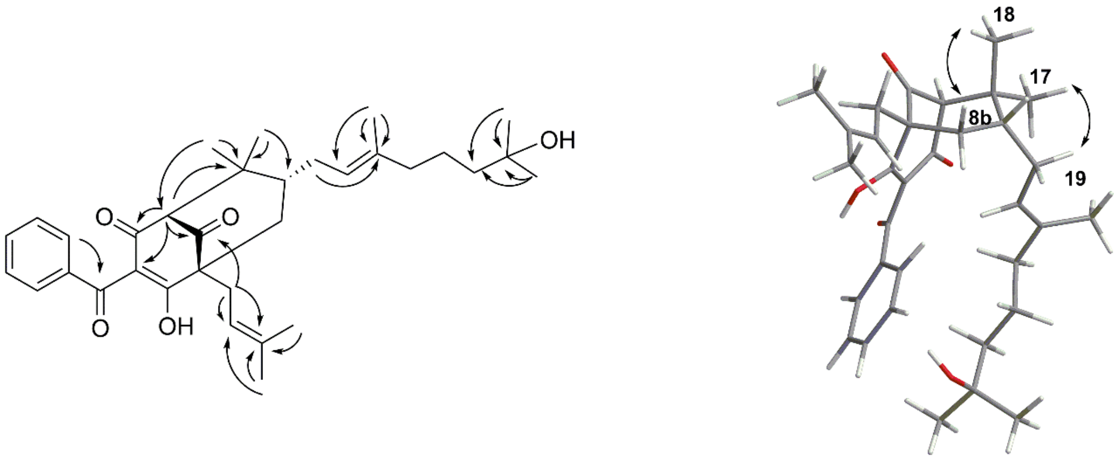

Figure S16. Key correlations observed in the HMBC and NOESY NMR spectra of **7**.

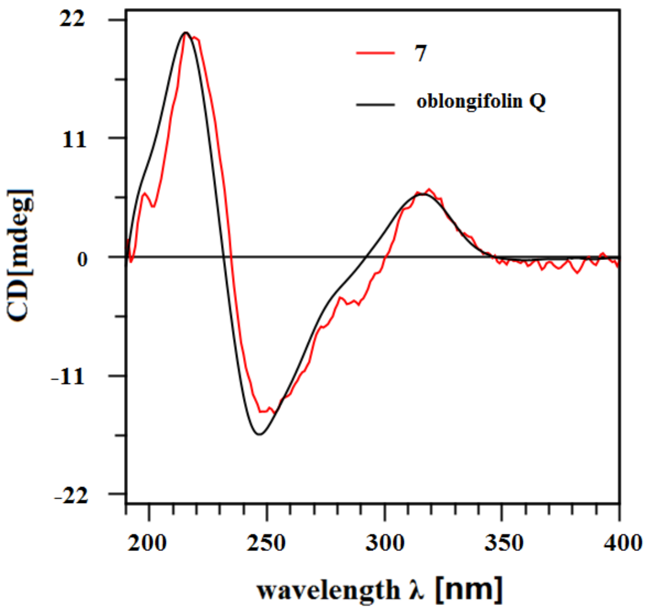

Figure S17. Experimental ECD spectra of **7** and oblongifolin Q

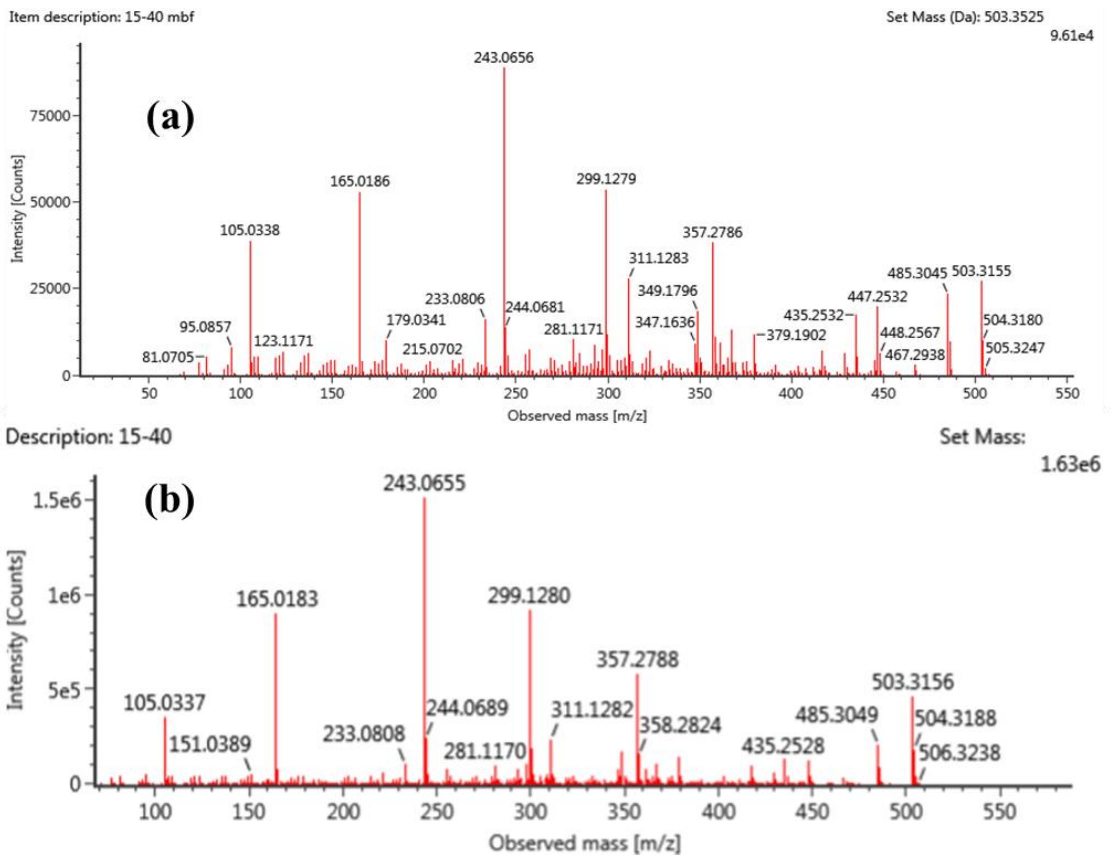

Figure S18. MS/MS spectra of **8** and **9** acquired by UPLC-PDA-QTOFMS analysis. (a) MS/MS spectrum of **8** for selected $[M + H]^+$ at *m*/*z* 503 (ramping collision energy 15–40 V); (b) MS/MS spectrum of **9** for selected $[M + H]^+$ at *m*/*z* 503 (ramping collision energy 15–40 V).

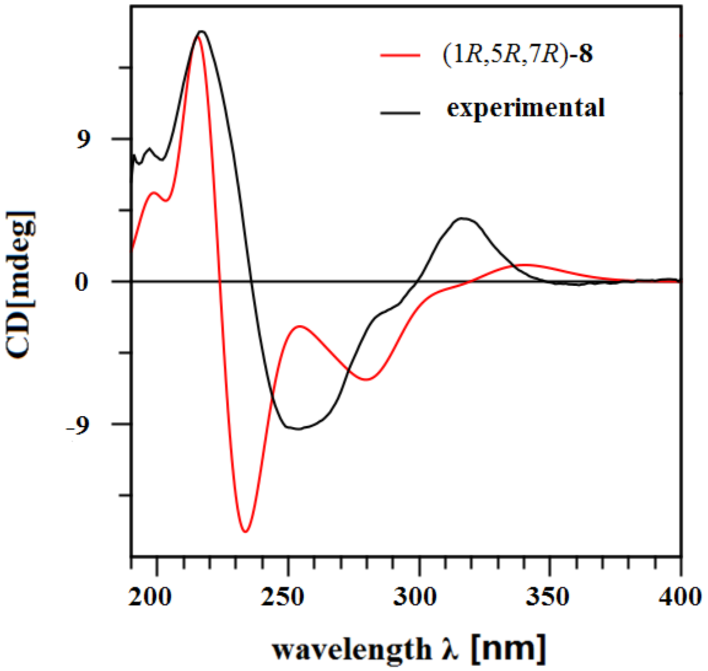

Figure S19. Calculated ECD spectrum of **8** its experimental curve.

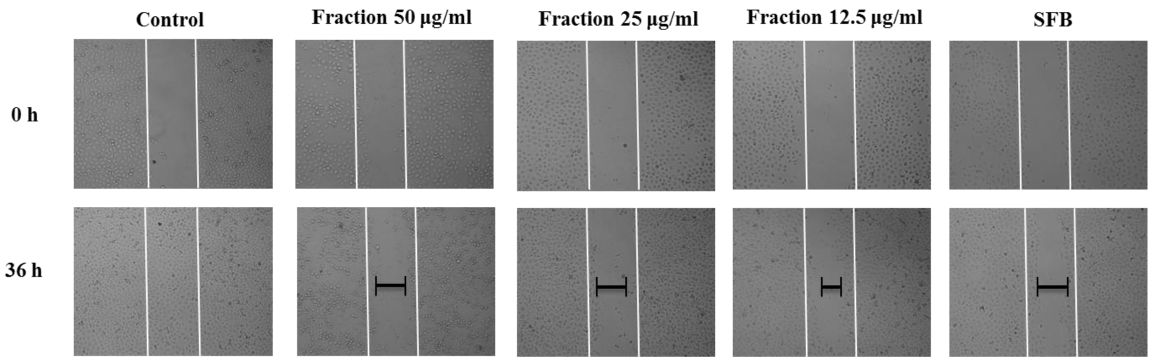

Figure S20. Anti-migration effect of the EtOAc-soluble fraction on human esophageal carcinoma cells (TE1) by wound healing assay. TE1 cells monolayer was scratched and treated with the EtOAc-soluble fraction at different concentrations of 12.5, 25, and 50 μg/ml. Sorafenib (SFB) was used as the positive control. Images were accessed by a microscope.

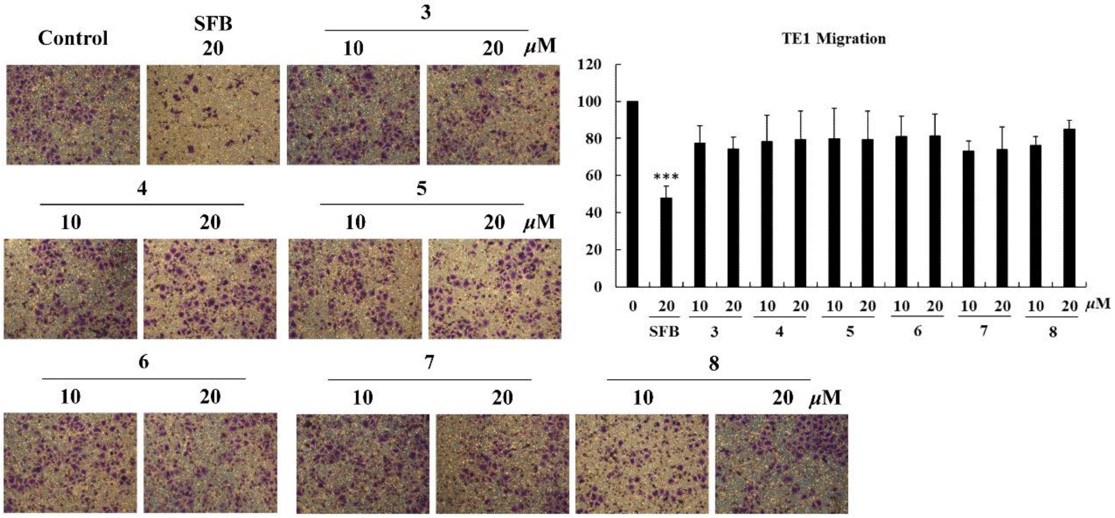

Figure S21. The effect of compounds **3–8** on migration in TE1 cells measured by transwell assay. Cells were incubated with **3–8** for 36 h, and migrated cells were fixed and stained with 0.1% crystal violet. Sorafenib (SFB) was used as the positive control. The summary data for transwell migration assay were presented as the means ± S.D. ***P < 0.001.

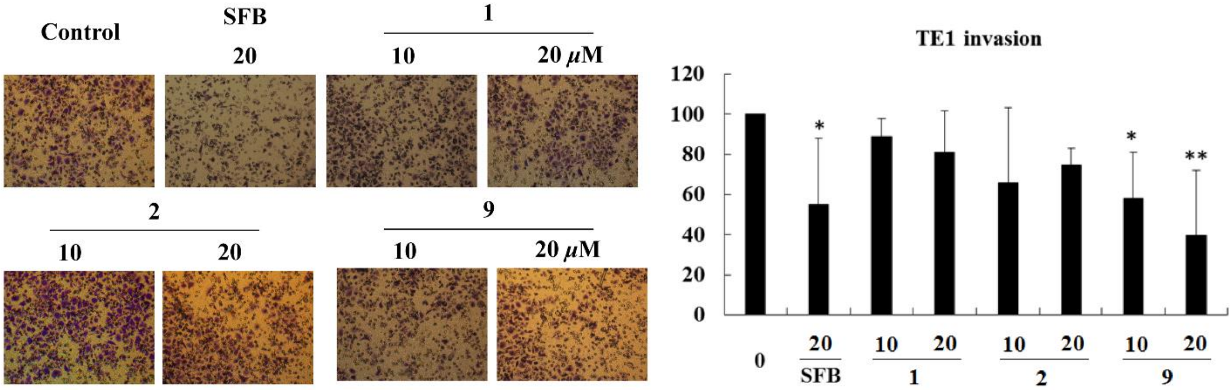

Figure S22. The effect of compounds **1**, **2**, and **9** on cell invasion was measured by matrigel coated transwell assays. Cells were incubated with compounds **1**, **2**, and **9** for 24 h, and invaded cells were fixed and stained with 0.1% crystal violet. The summary data for invasion assays were presented as the means ± S.D. *P < 0.01, **P < 0.01.

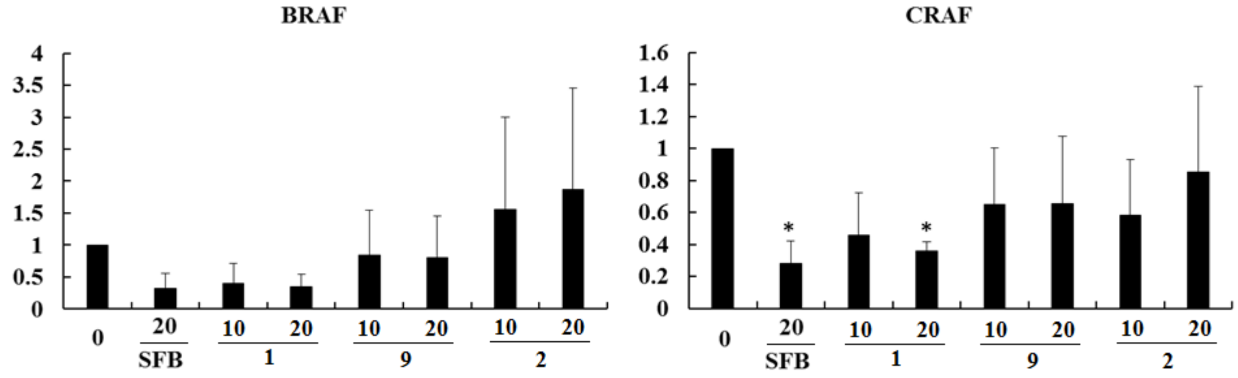

Figure S23. The effect of compounds **1**, **2**, and **9** on B-RAF and C-RAF mRNA levels. After treated with compounds **1**, **2**, and **9**, RNA was extracted and the B-RAF and C-RAF mRNA levels were analyzed by Real-Time PCR. Data are analyzed by One-way ANNOVA and presented as the means ± S.D. *$P < 0.05$

Composition	i-FIT Confidence (%)	m/z RMS (PPM)	Intensity RMS (%)	Predicted m/z	m/z error (PPM)	m/z error (mDa)	DBE
C24H30O8	100.000000	36.675014	434.638550	447.201344	-0.099480	-0.044387	10.000000

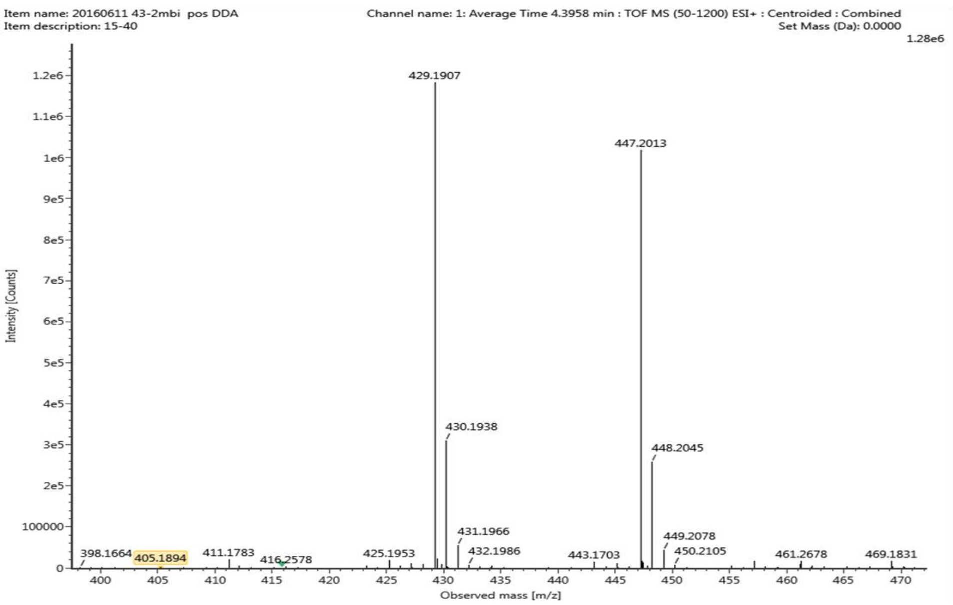

Figure S24. HRESIMS spectrum of **1**

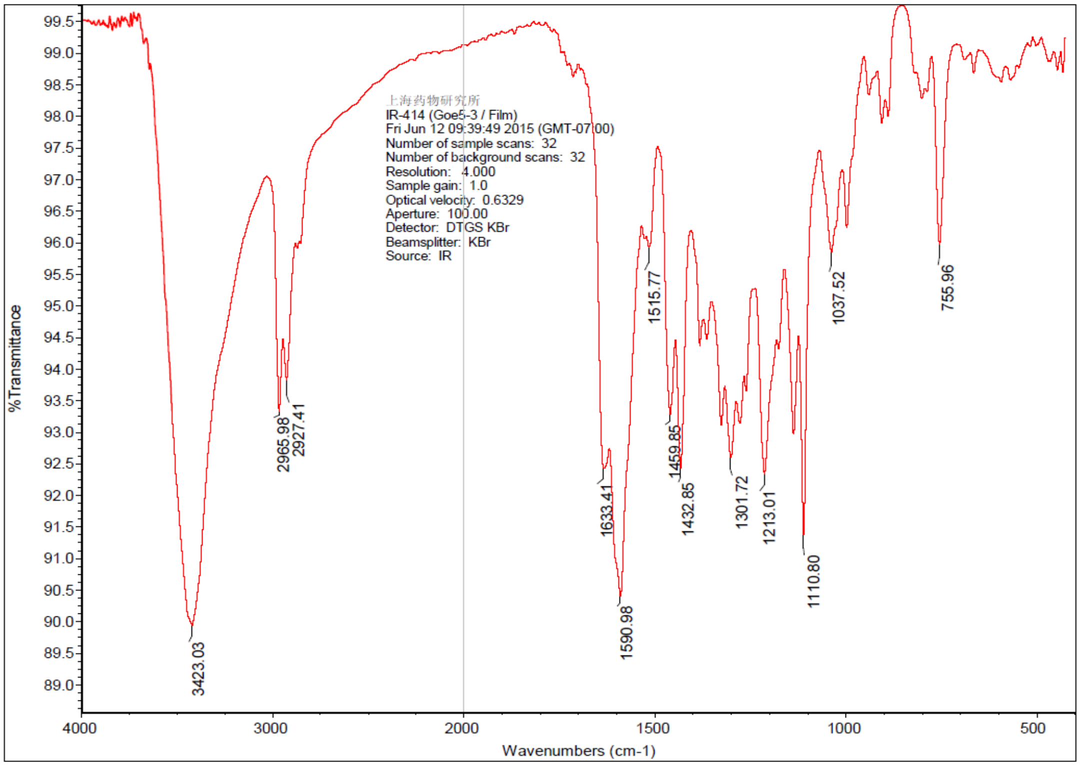

Figure S25. IR (KBr, disc) spectrum of **1**

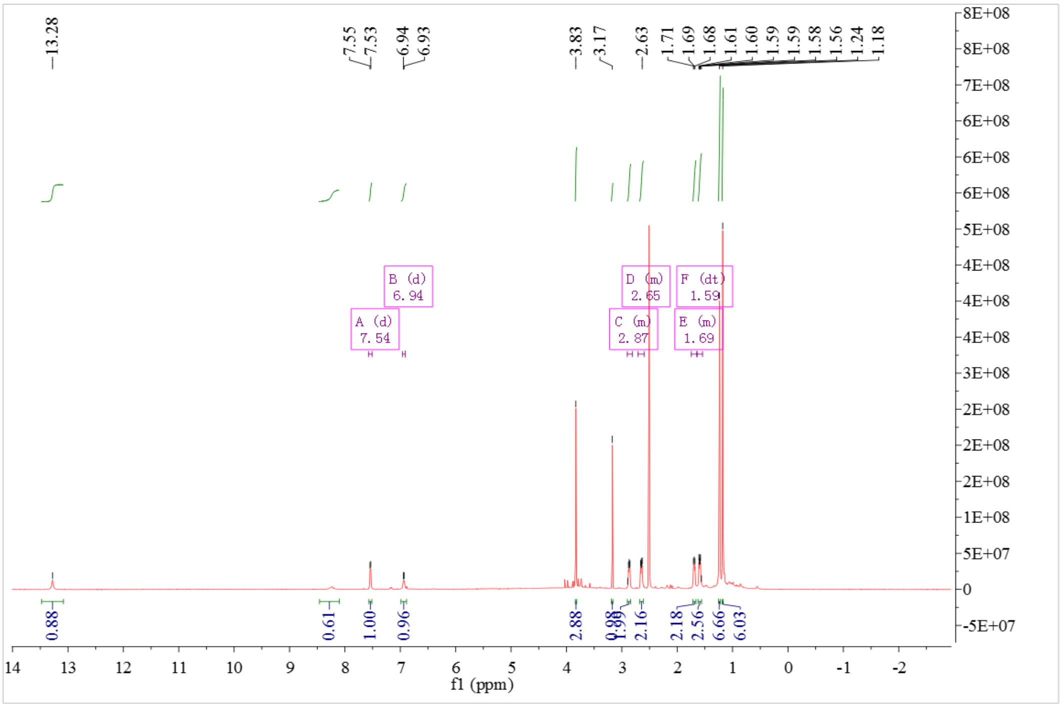

Figure S26. ^{1}H NMR spectrum (DMSO-d_6, 600 MHz) of **1**

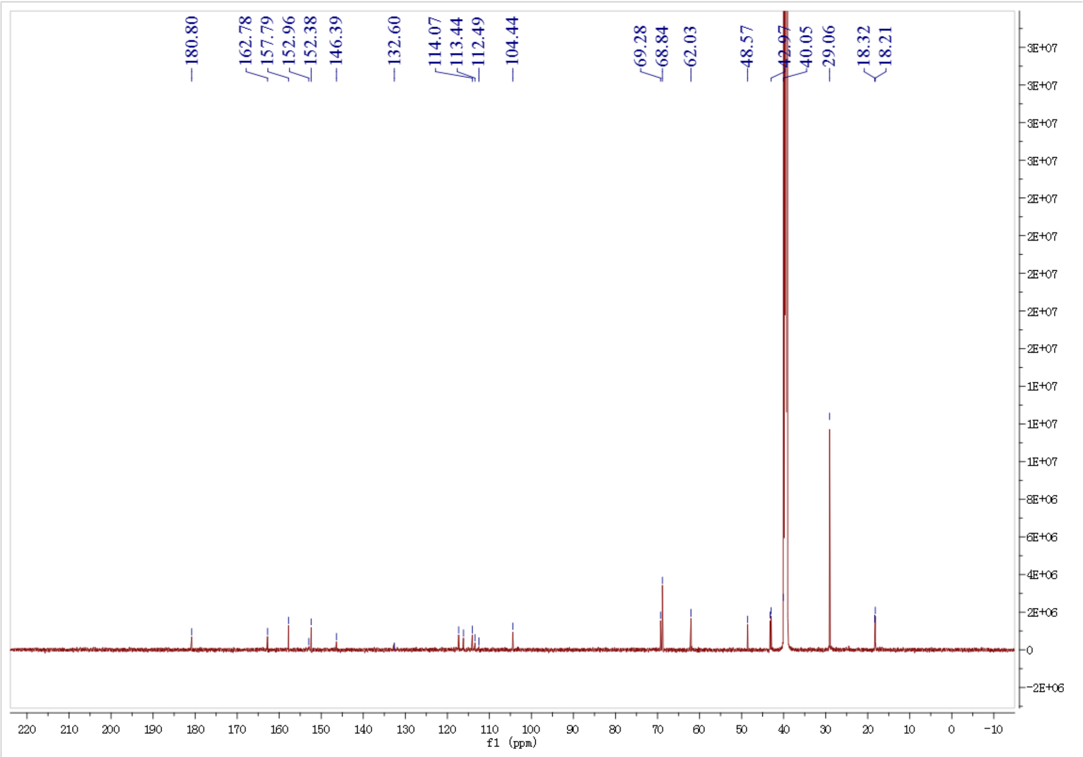

Figure S27. ^{13}C NMR spectrum (DMSO-d_6, 150 MHz) of **1**

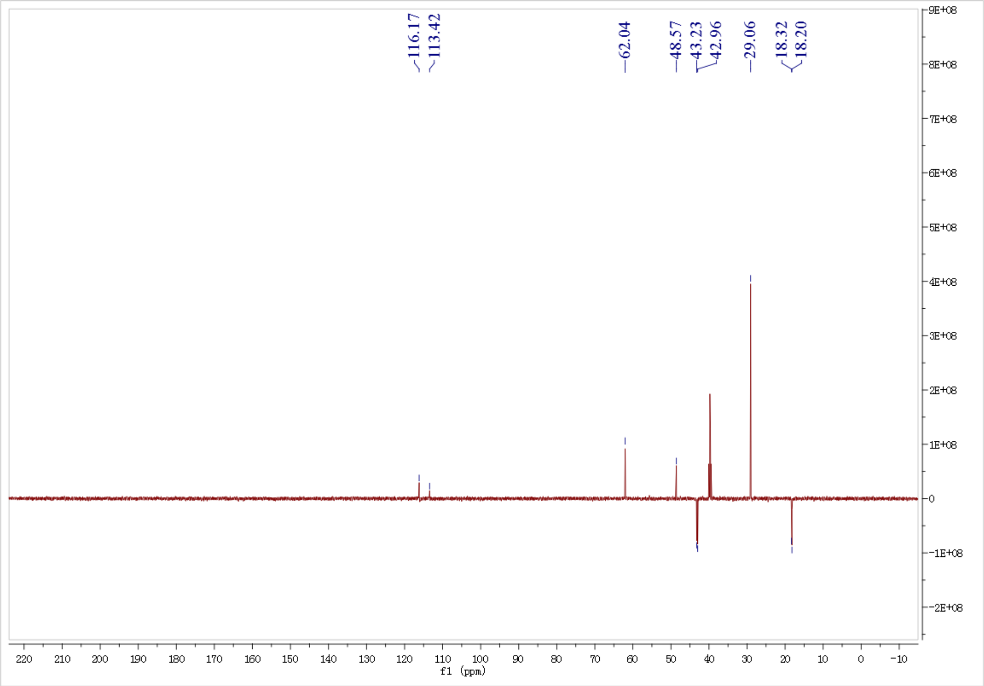

Figure S28. DEPT NMR spectrum (DMSO-d_6, 150 MHz) of **1**

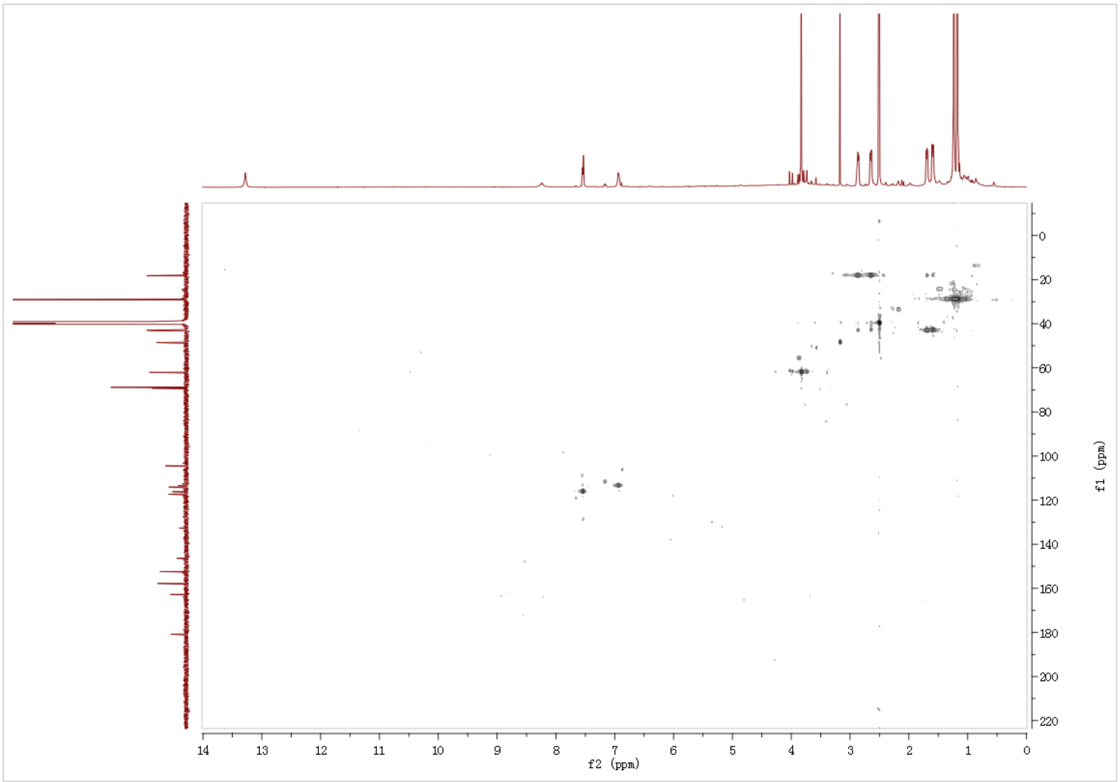

Figure S29. HSQC NMR spectrum (DMSO-d_6, 600 MHz, 150 MHz) of **1**

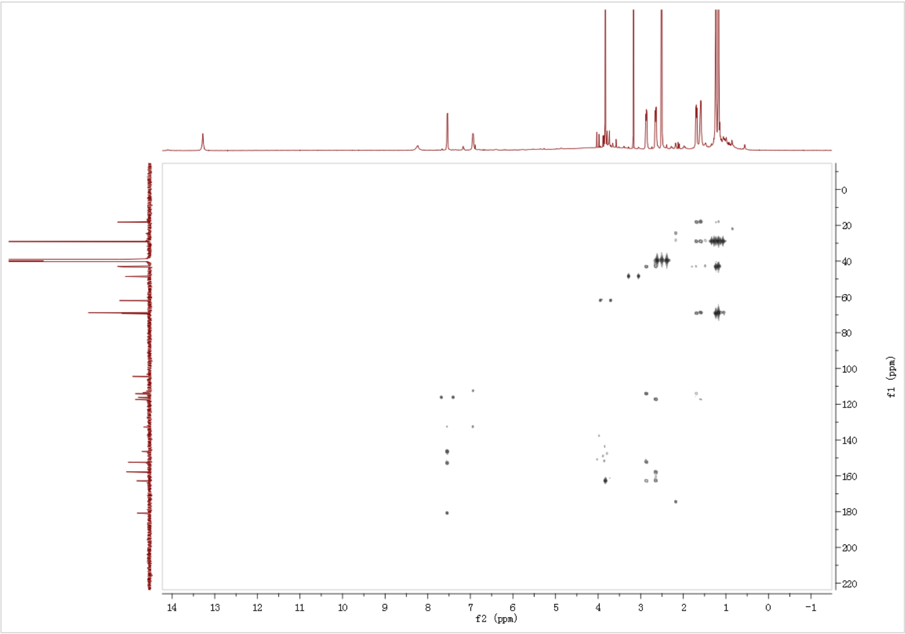

Figure S30. HMBC NMR spectrum (DMSO-d_6, 600 MHz, 150 MHz) of **1**

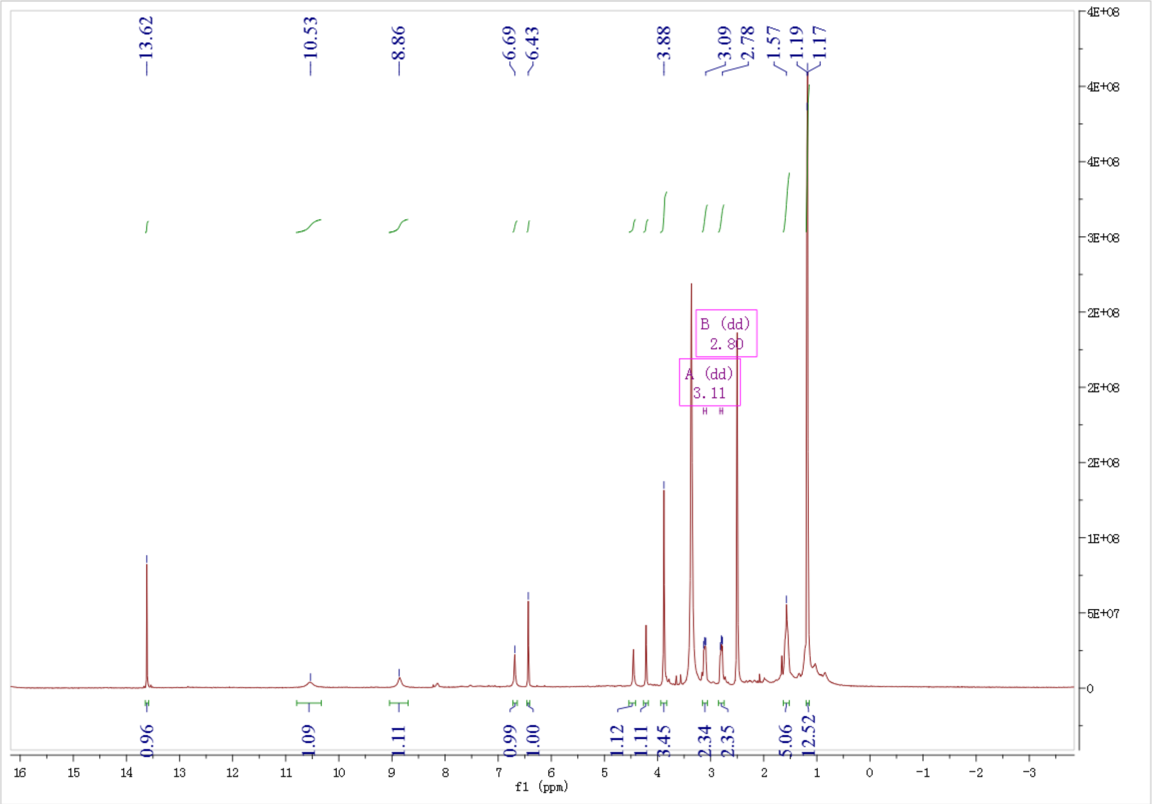

Figure S31. ^{1}H NMR spectrum (DMSO-d_6, 400 MHz) of **2**

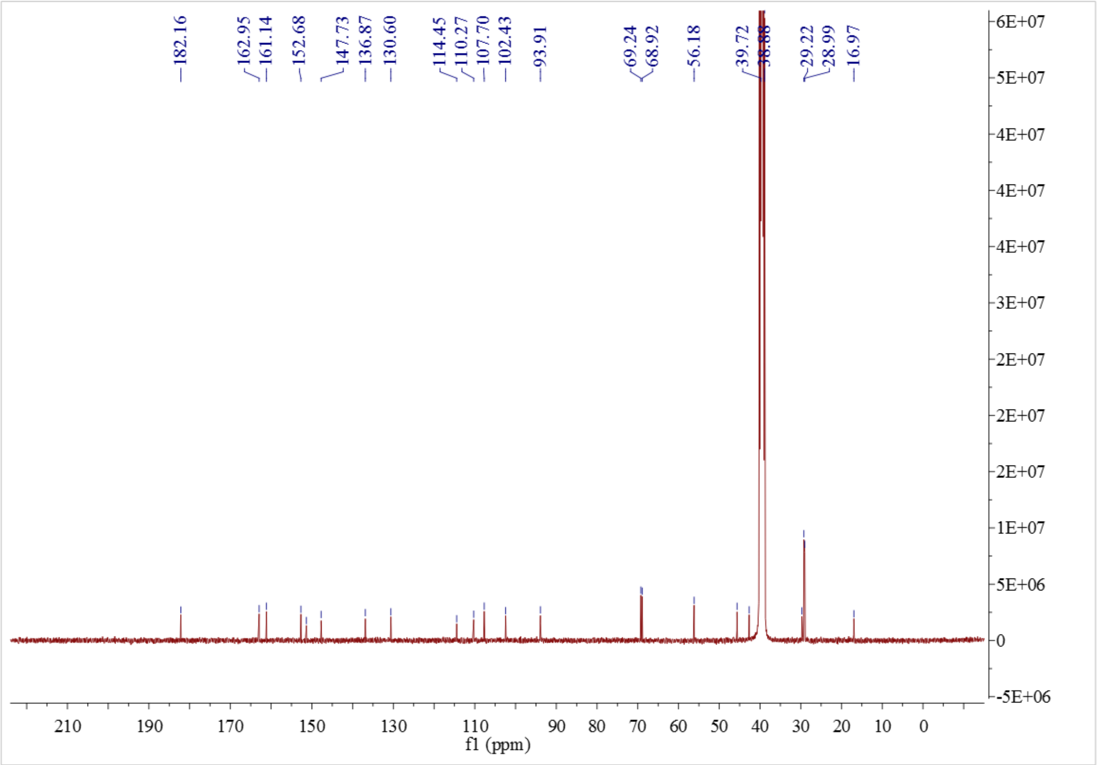

Figure S32. ^{13}C NMR spectrum (DMSO-d_6, 100 MHz) of **2**

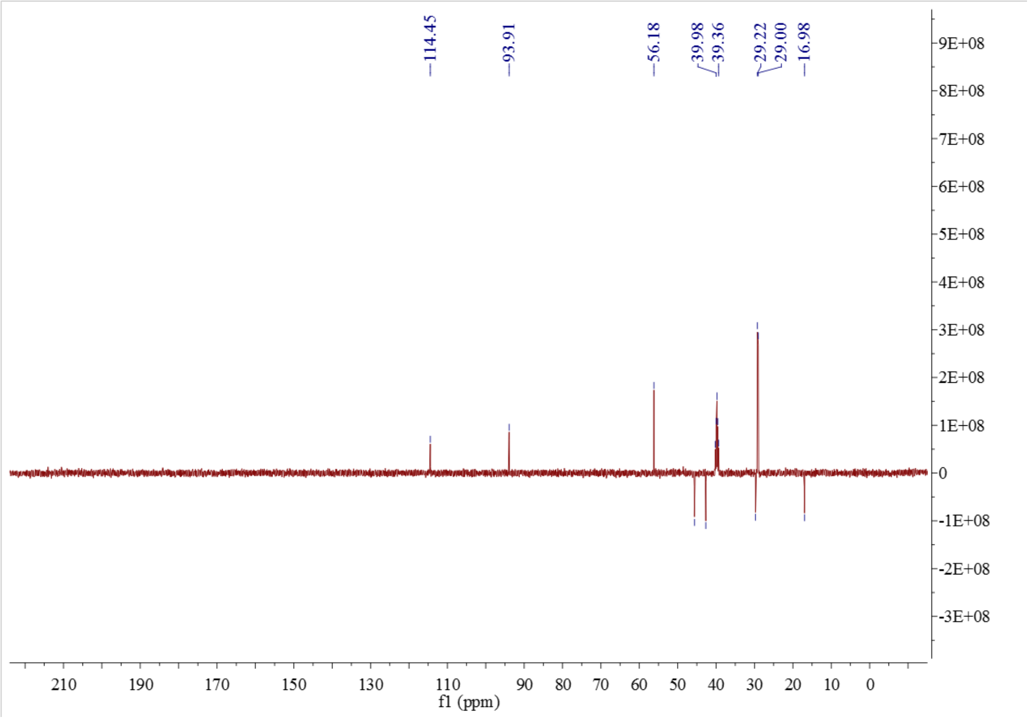

Figure S33. DEPT NMR spectrum (DMSO-d_6, 100 MHz) of **2**

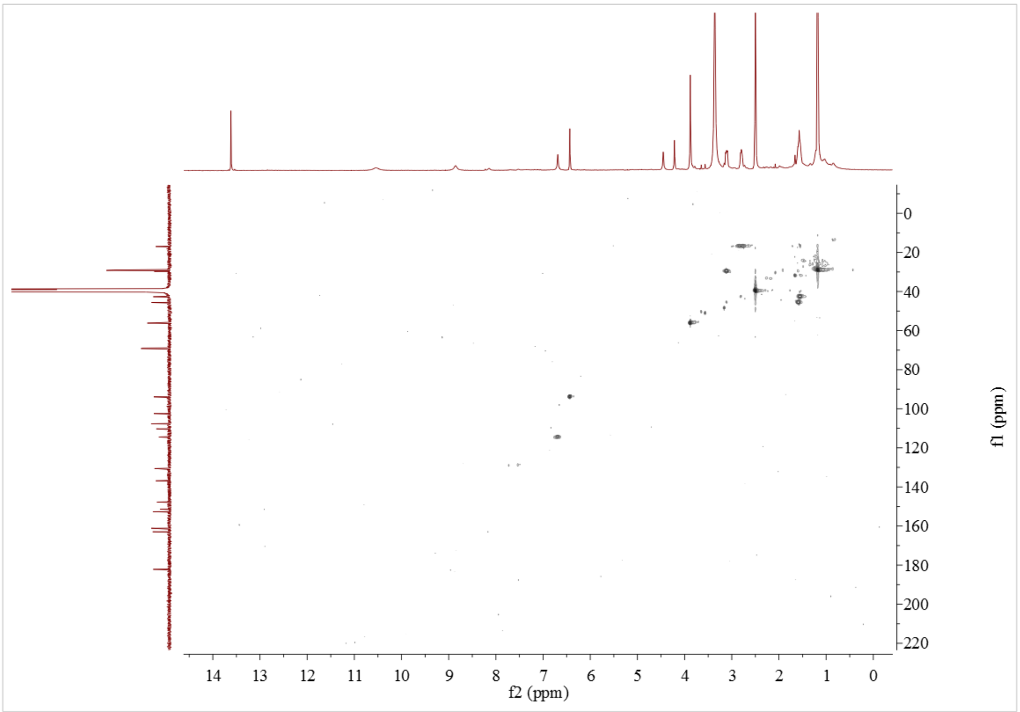

Figure S34. HSQC NMR spectrum (DMSO-d_6, 400 MHz, 100 MHz) of **2**

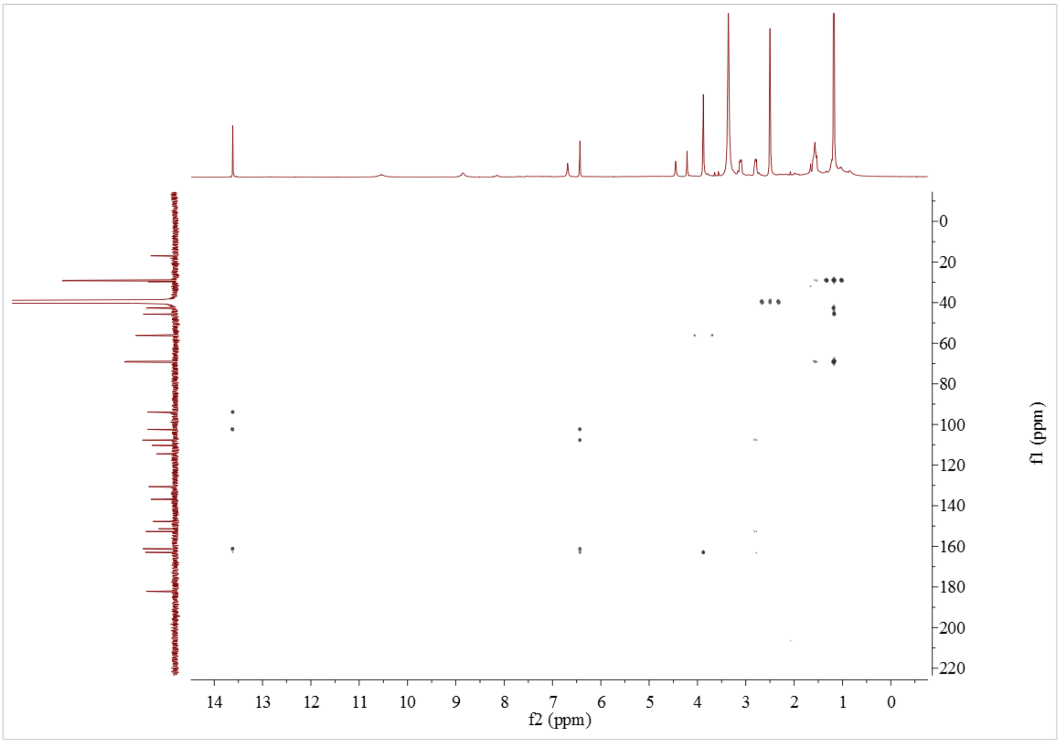

Figure S35. HMBC NMR spectrum (DMSO-d_6, 400 MHz, 100 MHz) of **2**

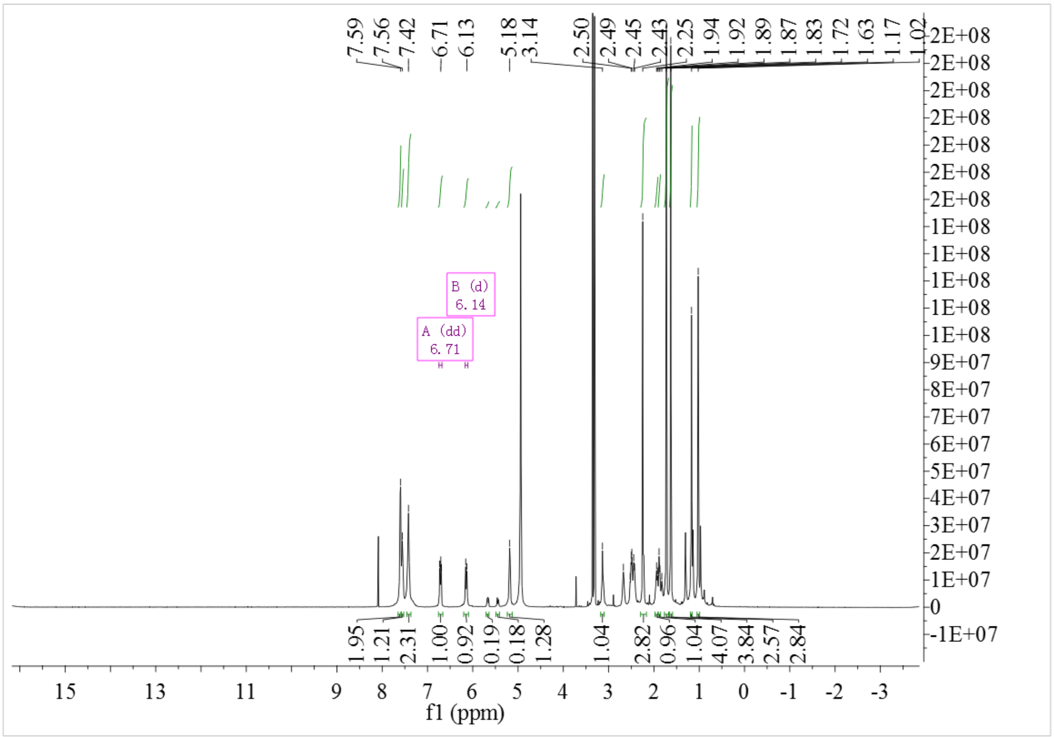

Figure S36. ^{1}H NMR spectrum (CD_3OD/0.1% TFA, 600 MHz) of **3**

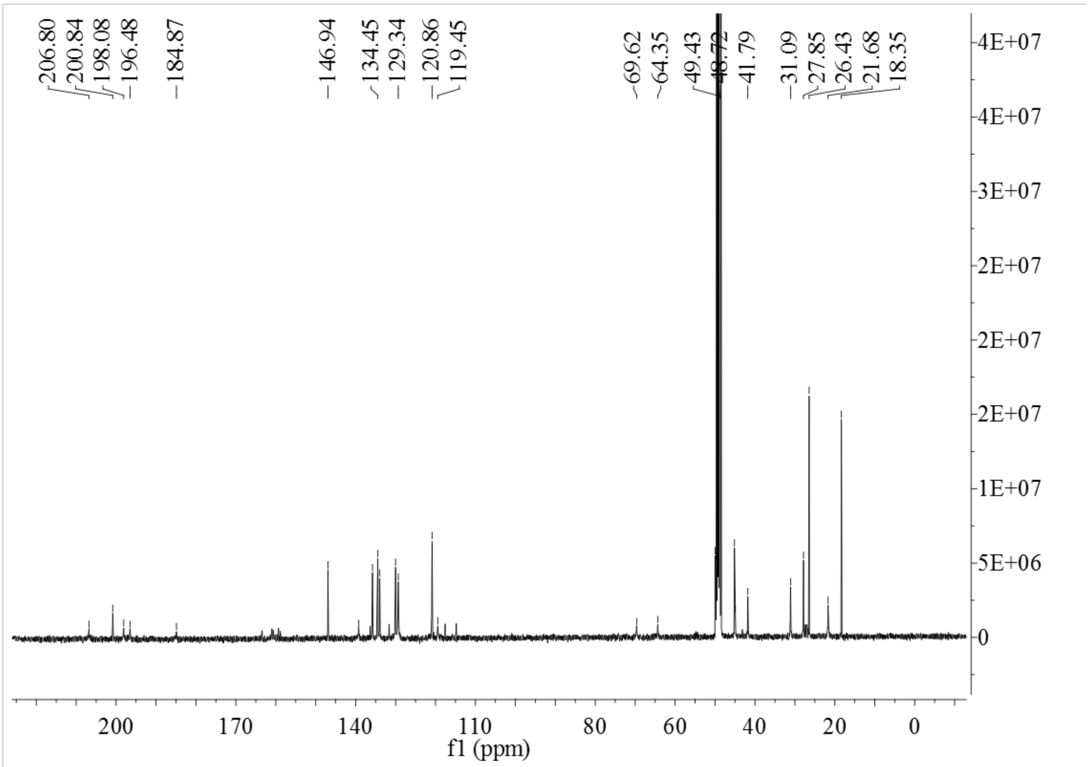

Figure S37. ^{13}C NMR spectrum (CD_3OD/0.1% TFA, 150 MHz) of **3**

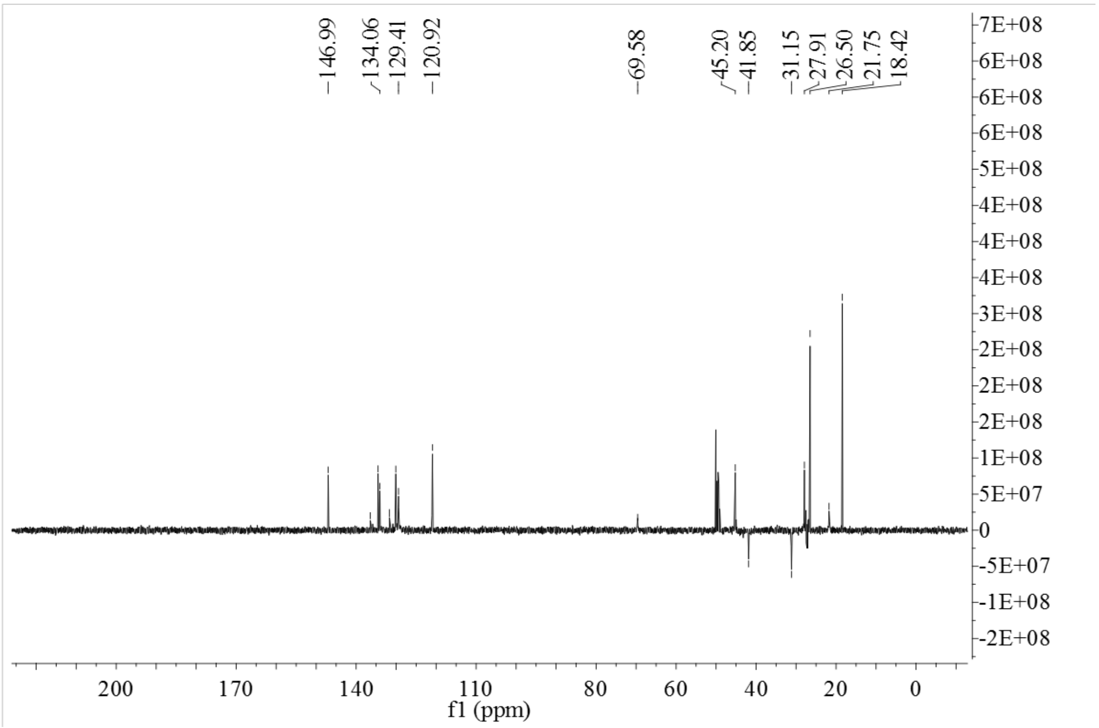

Figure S38. DEPT NMR spectrum (CD_3OD/0.1% TFA, 150 MHz) of **3**

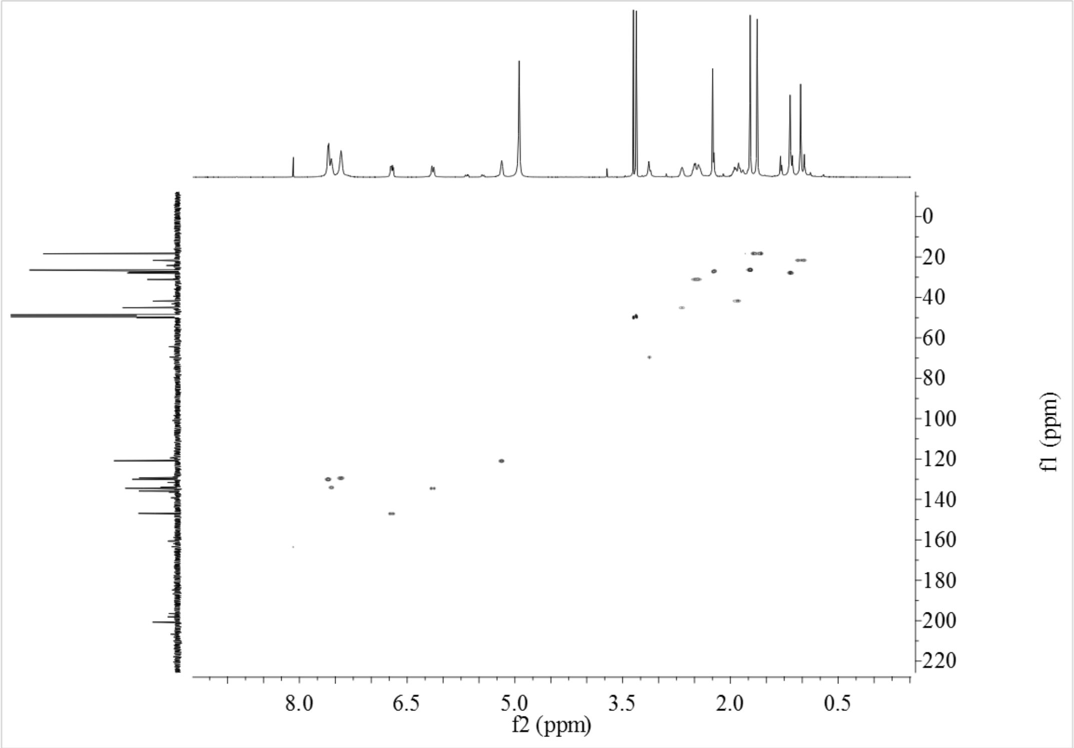

Figure S39. HSQC NMR spectrum (CD_3OD/0.1% TFA, 600 MHz, 150 MHz) of **3**

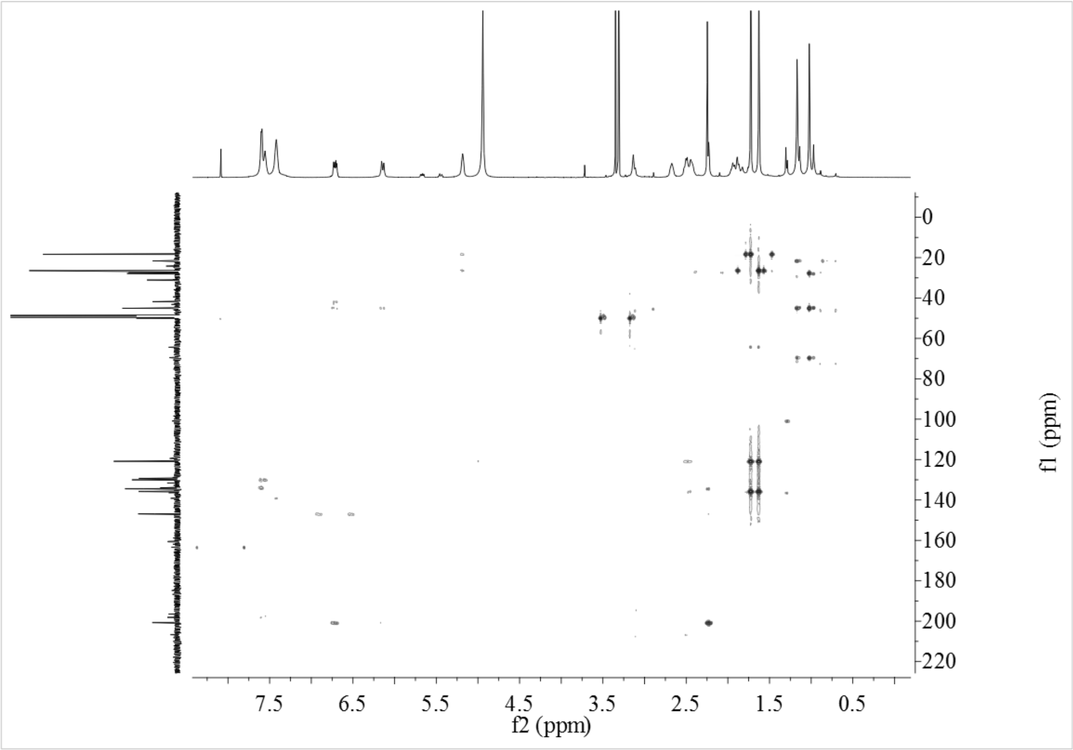

Figure S40. HMBC NMR spectrum (CD_3OD/0.1% TFA, 600 MHz, 150 MHz) of **3**

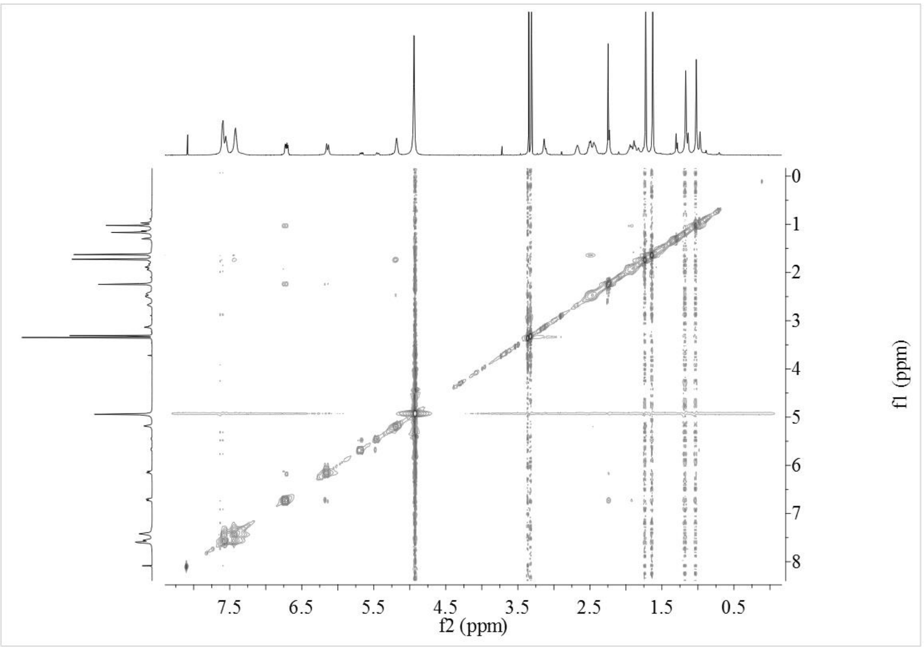

Figure S41. NOSEY NMR spectrum (CD_3OD/0.1% TFA, 600 MHz) of **3**

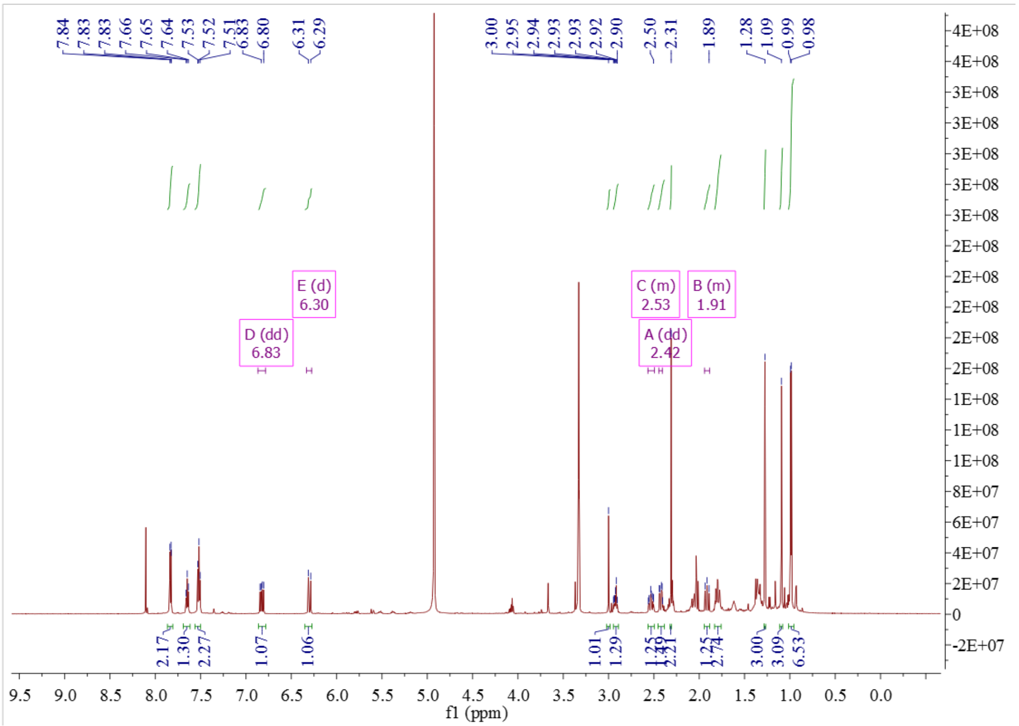

Figure S42. ^{1}H NMR spectrum (CD_3OD/0.1% TFA, 600 MHz) of **4**

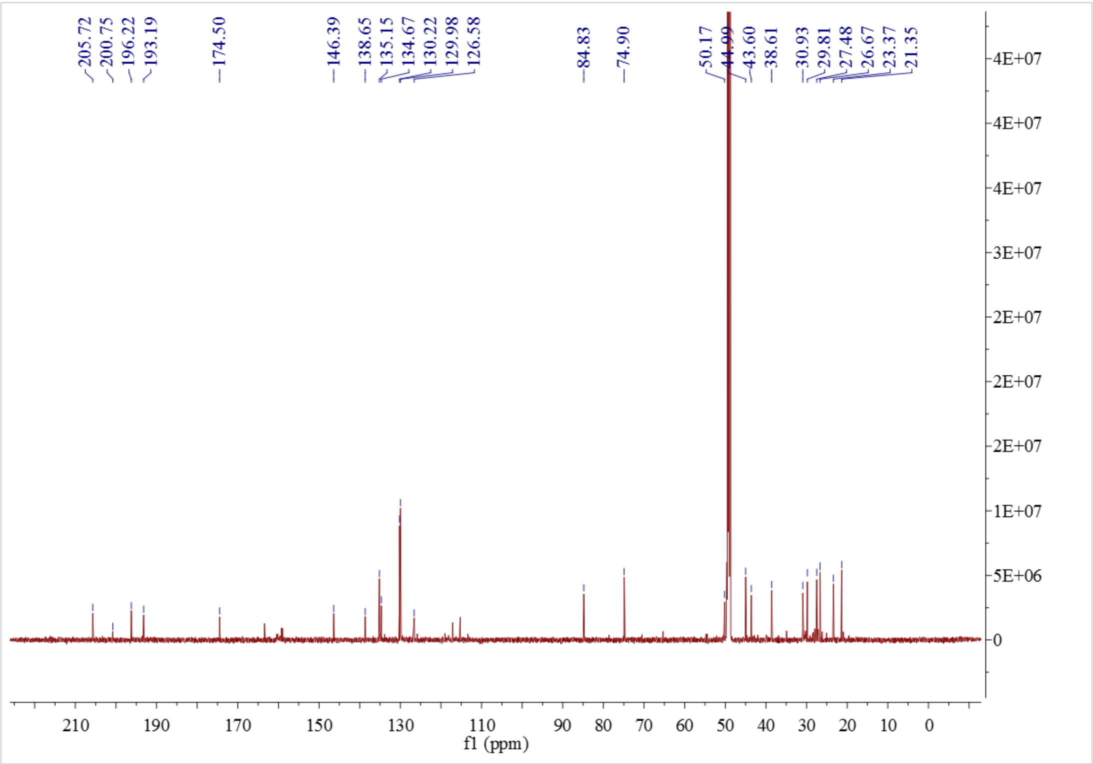

Figure S43. ^{13}C NMR spectrum (CD_3OD/0.1% TFA, 150 MHz) of **4**

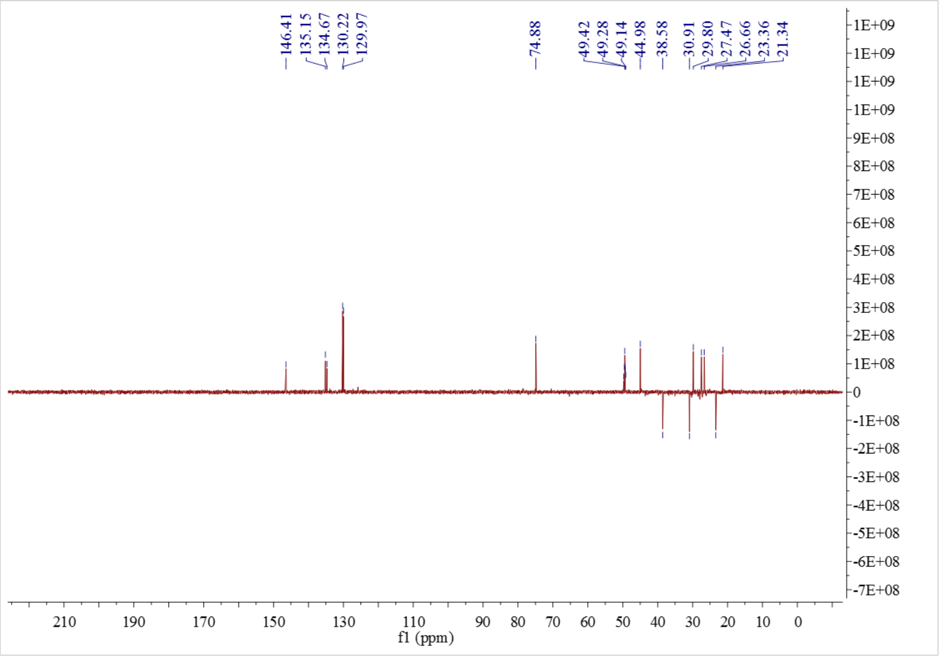

Figure S44. DEPT NMR spectrum (CD_3OD/0.1% TFA, 150 MHz) of **4**

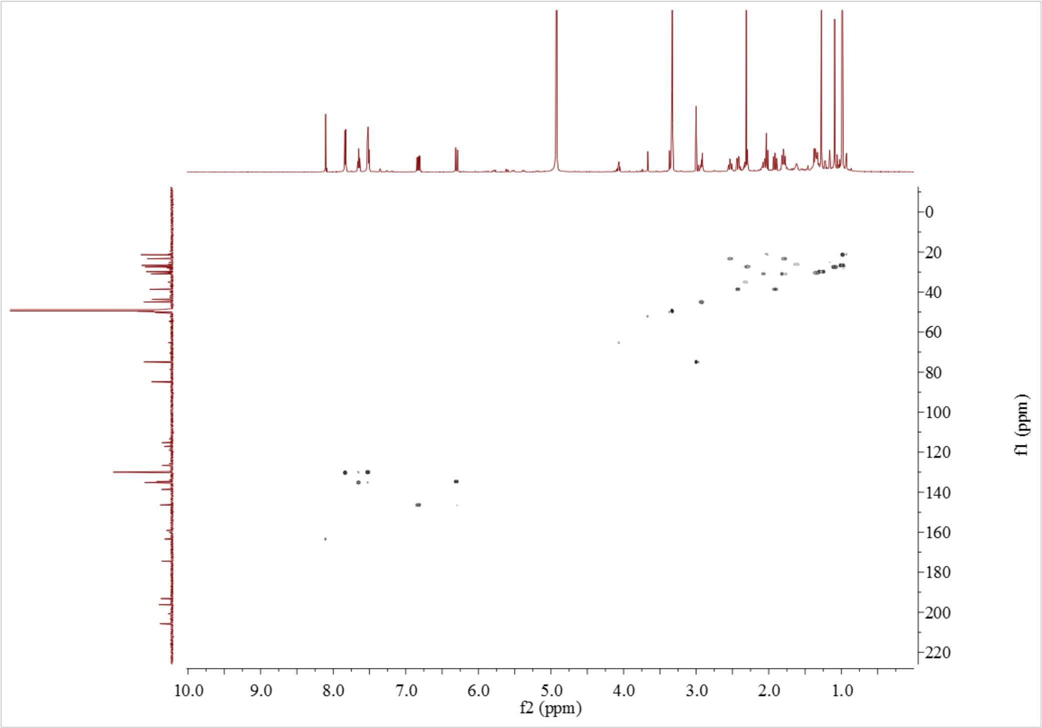

Figure S45. HSQC NMR spectrum (CD_3OD/0.1% TFA, 600 MHz, 150 MHz) of **4**

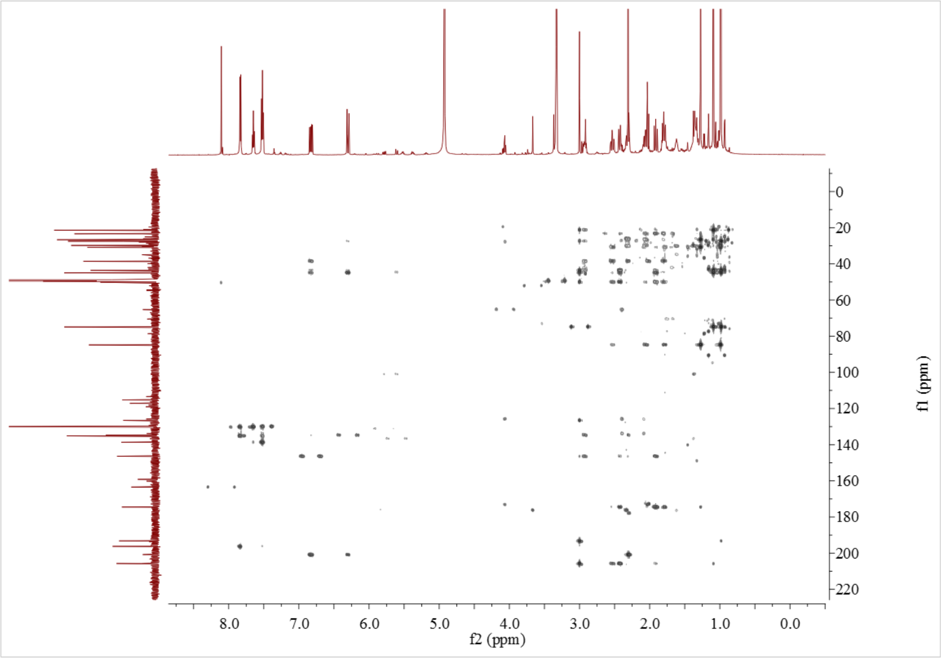

Figure S46. HMBC NMR spectrum (CD_3OD/0.1% TFA, 600 MHz, 150 MHz) of **4**

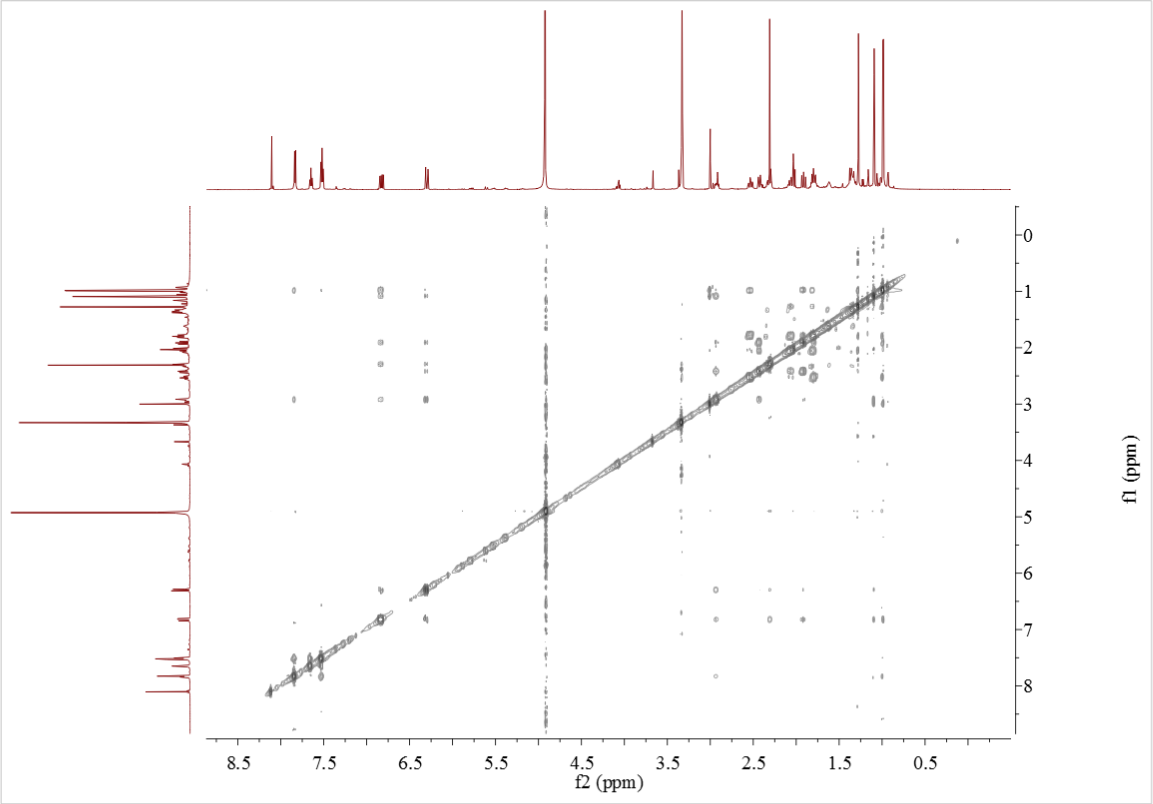

Figure S47. NOSEY NMR spectrum (CD_3OD/0.1% TFA, 600 MHz) of **4**

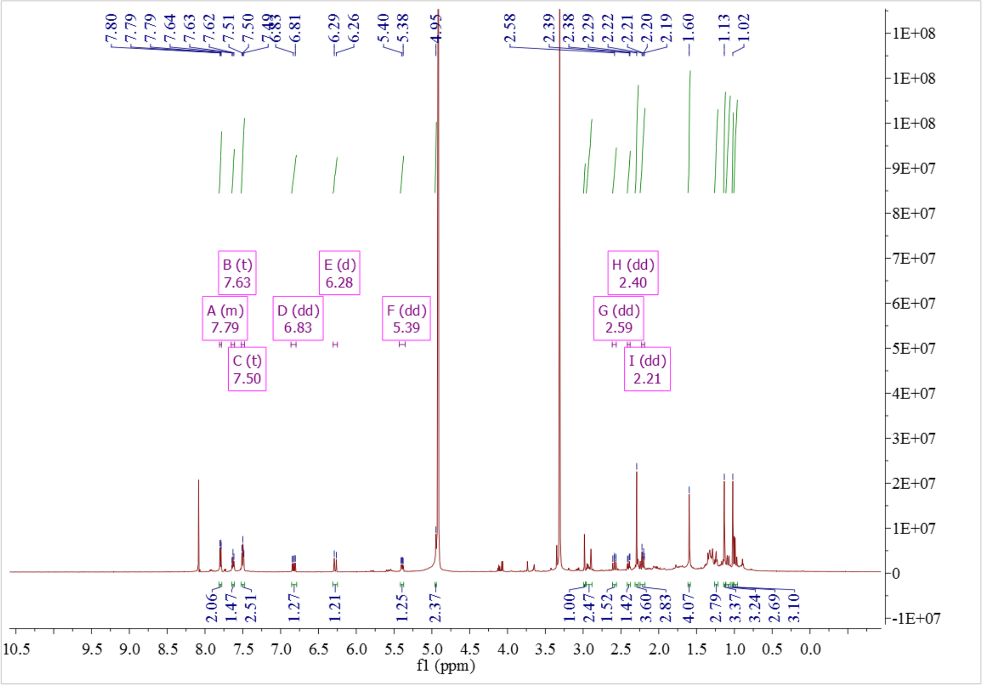

Figure S48. ^{1}H NMR spectrum (CD$_3$OD/0.1% TFA, 600 MHz) of **5**

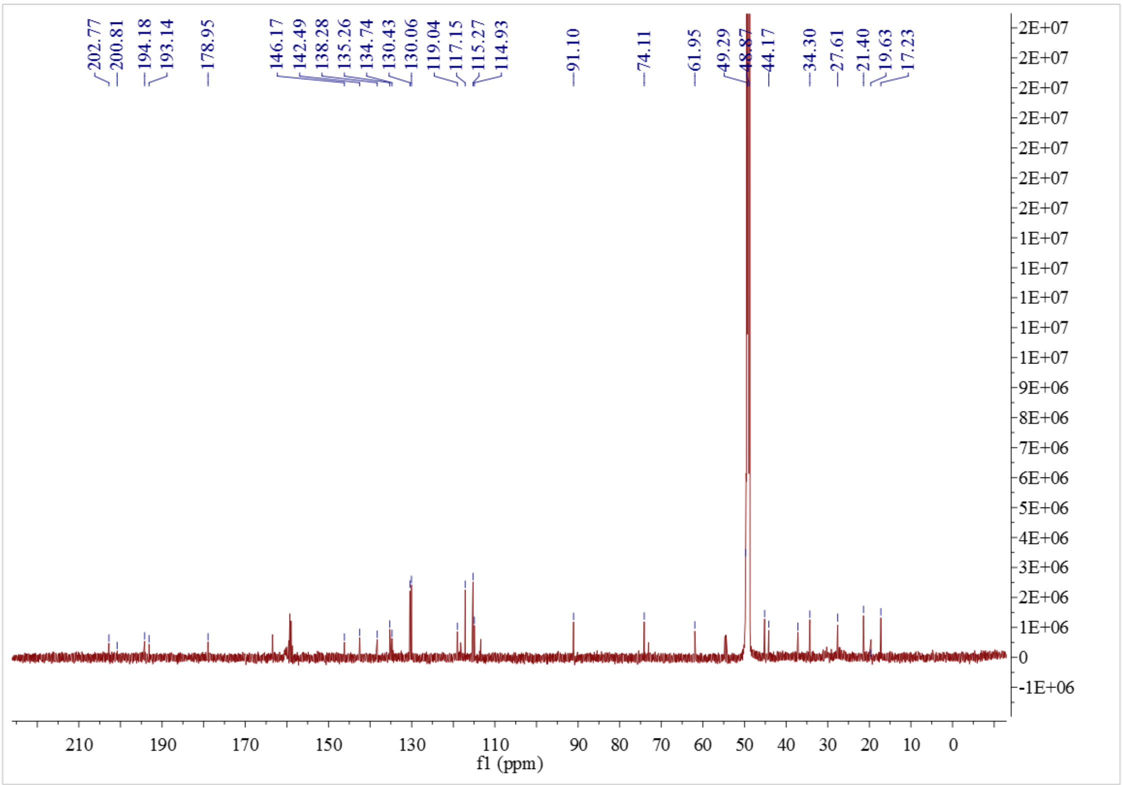

Figure S49. ^{13}C NMR spectrum (CD$_3$OD/0.1% TFA, 150 MHz) of **5**

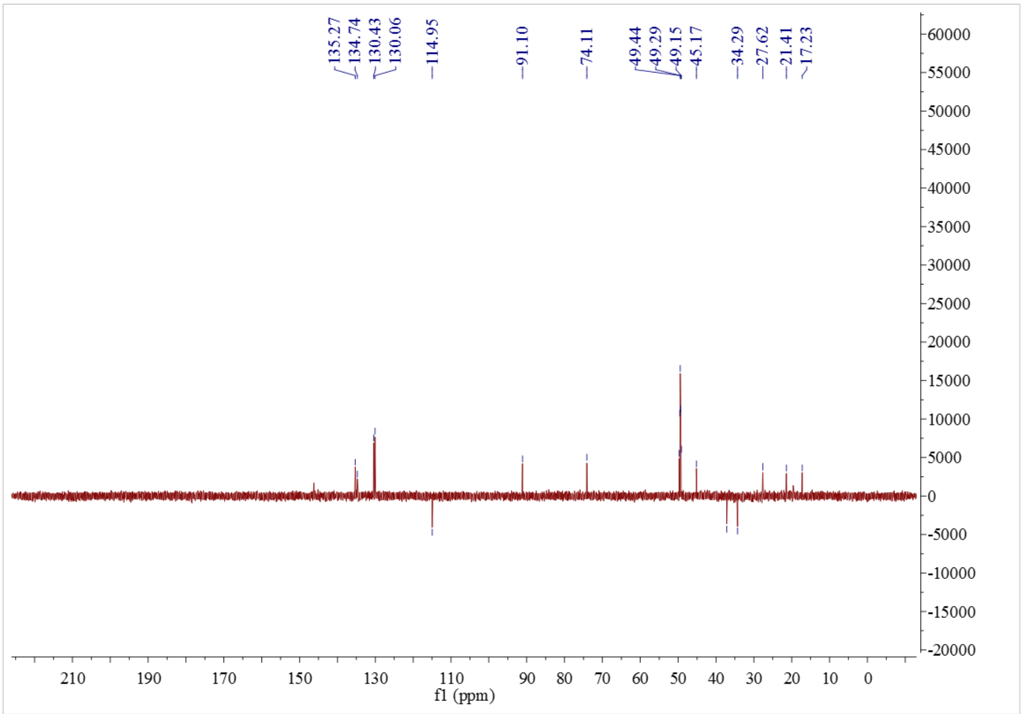

Figure S50. DEPT NMR spectrum (CD_3OD/0.1% TFA, 150 MHz) of **5**

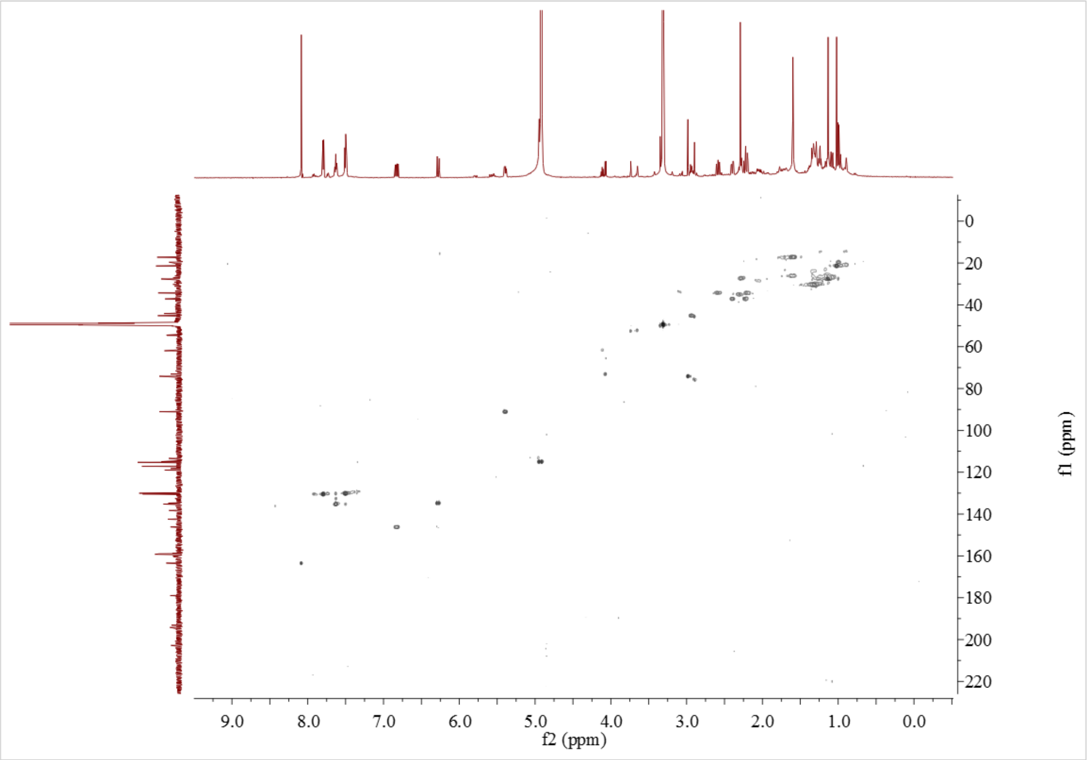

Figure S51. HSQC NMR spectrum (CD_3OD/0.1% TFA, 600 MHz, 150 MHz) of **5**

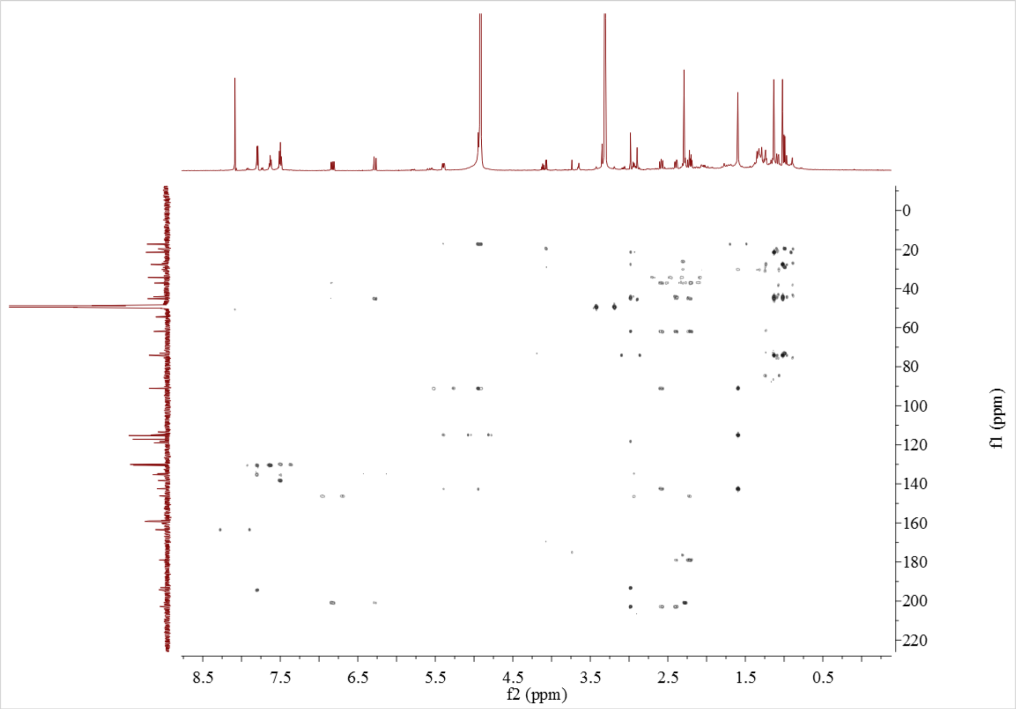

Figure S52. HMBC NMR spectrum (CD_3OD/0.1% TFA, 600 MHz, 150 MHz) of **5**

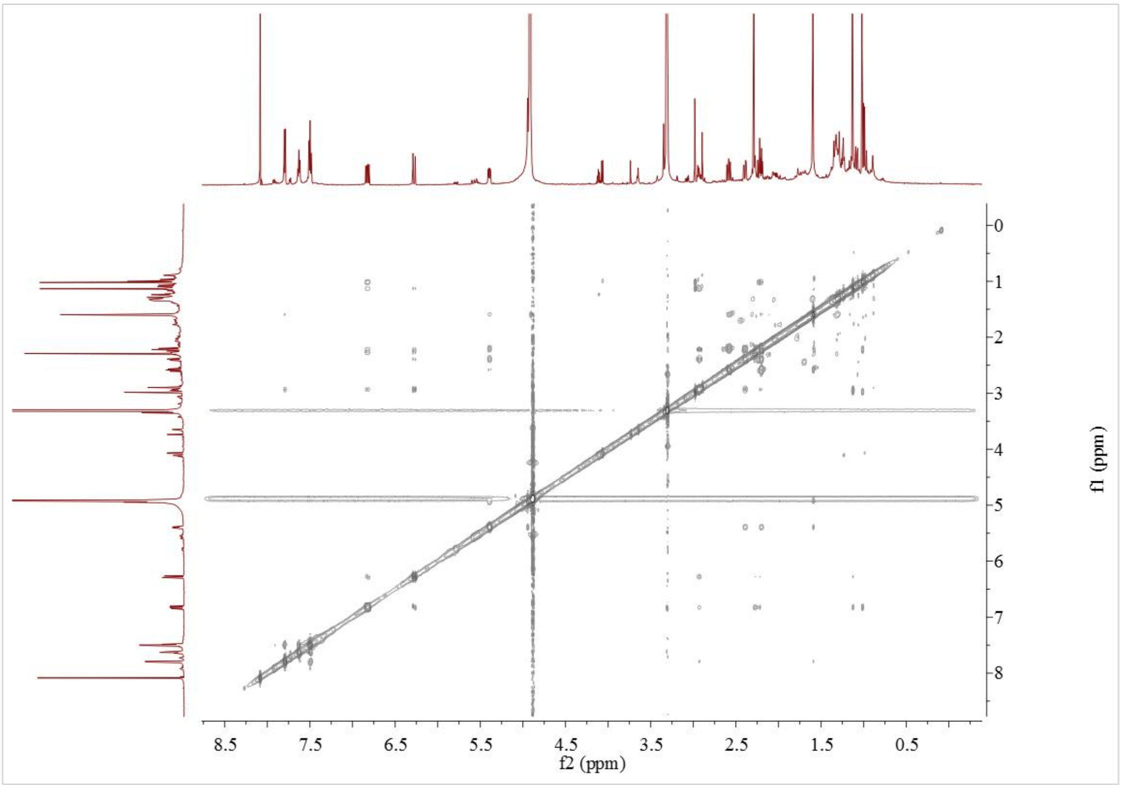

Figure S53. NOSEY NMR spectrum (CD_3OD/0.1% TFA, 600 MHz) of **5**

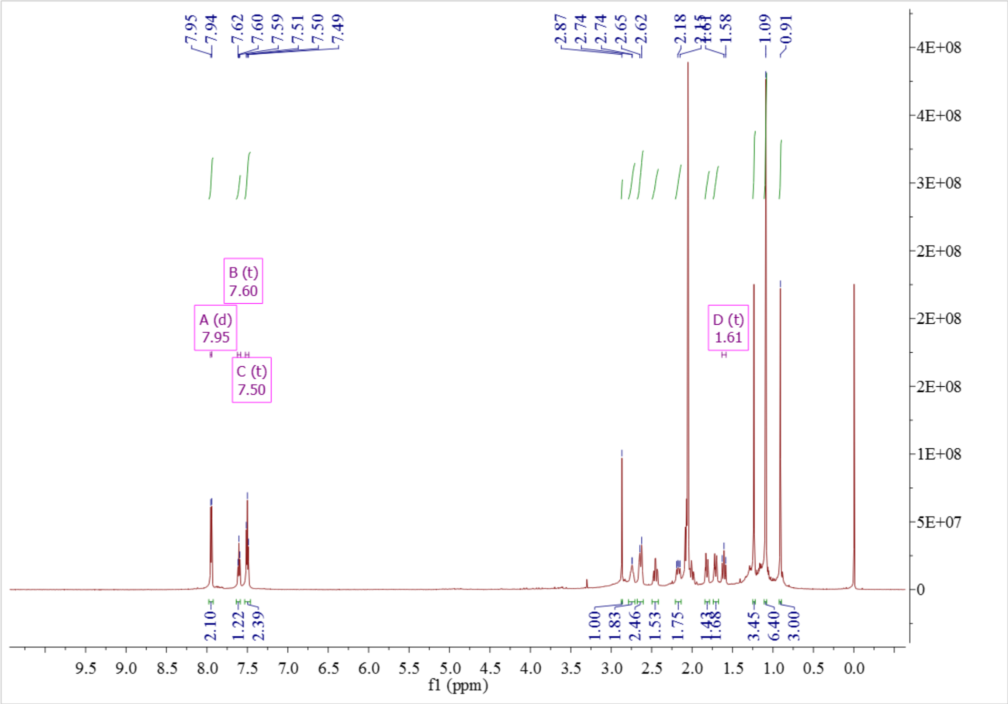

Figure S54. ^{1}H NMR spectrum (Acetone-d_6, 600 MHz) of **6**

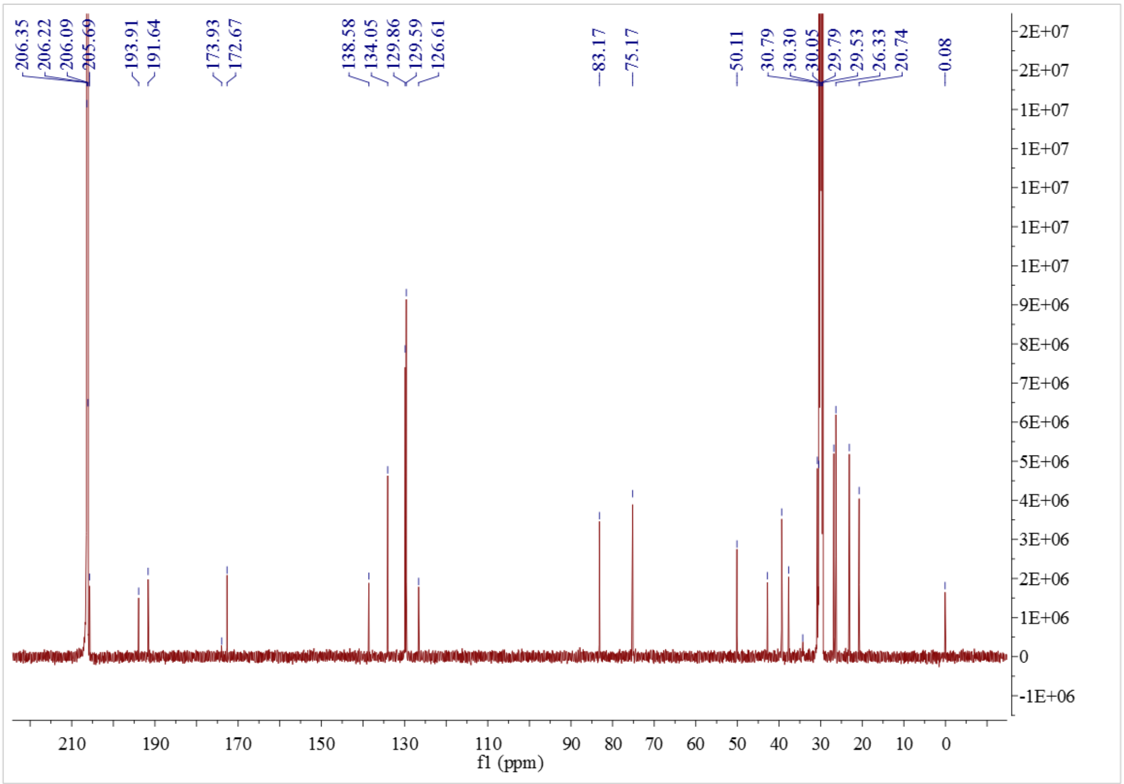

Figure S55. ^{13}C NMR spectrum (Acetone-d_6, 150 MHz) of **6**

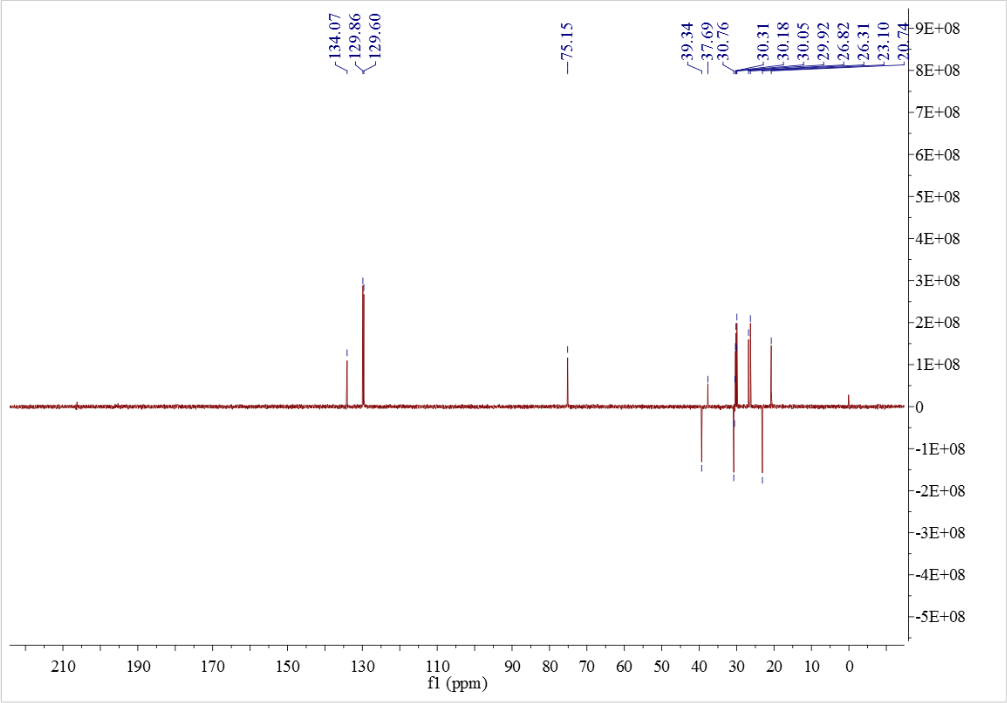

Figure S56. DEPT NMR spectrum (Acetone-d_6, 150 MHz) of **6**

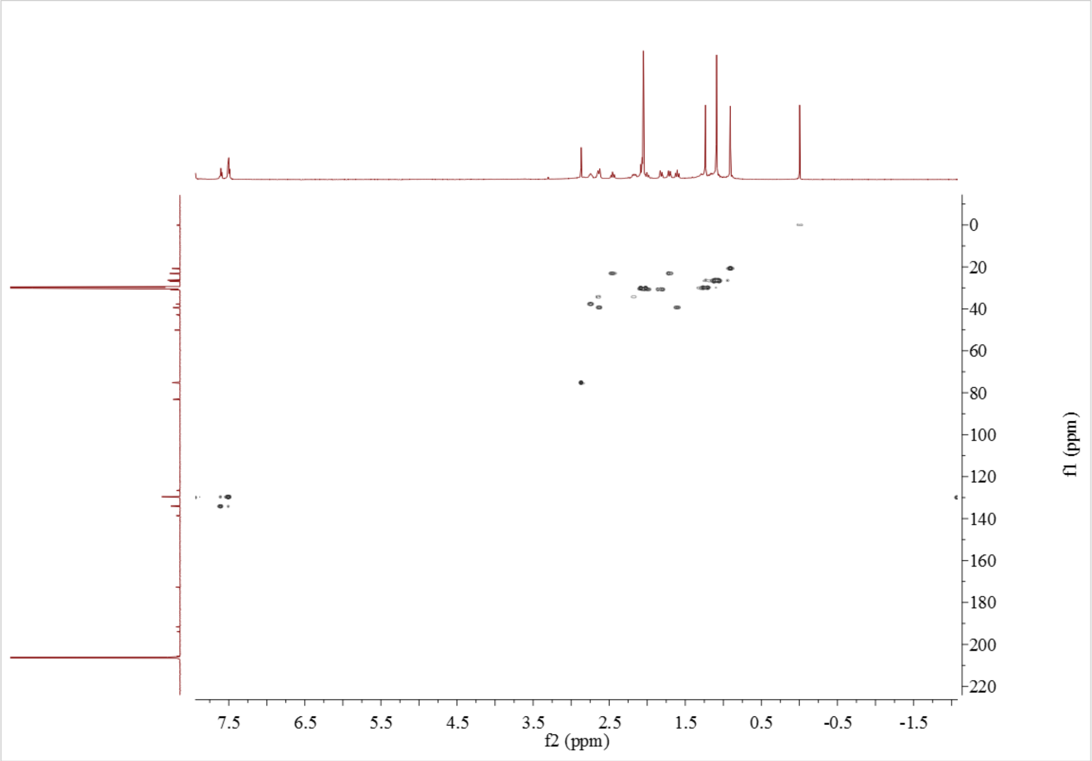

Figure S57. HSQC NMR spectrum (Acetone-d_6, 600 MHz, 150 MHz) of **6**

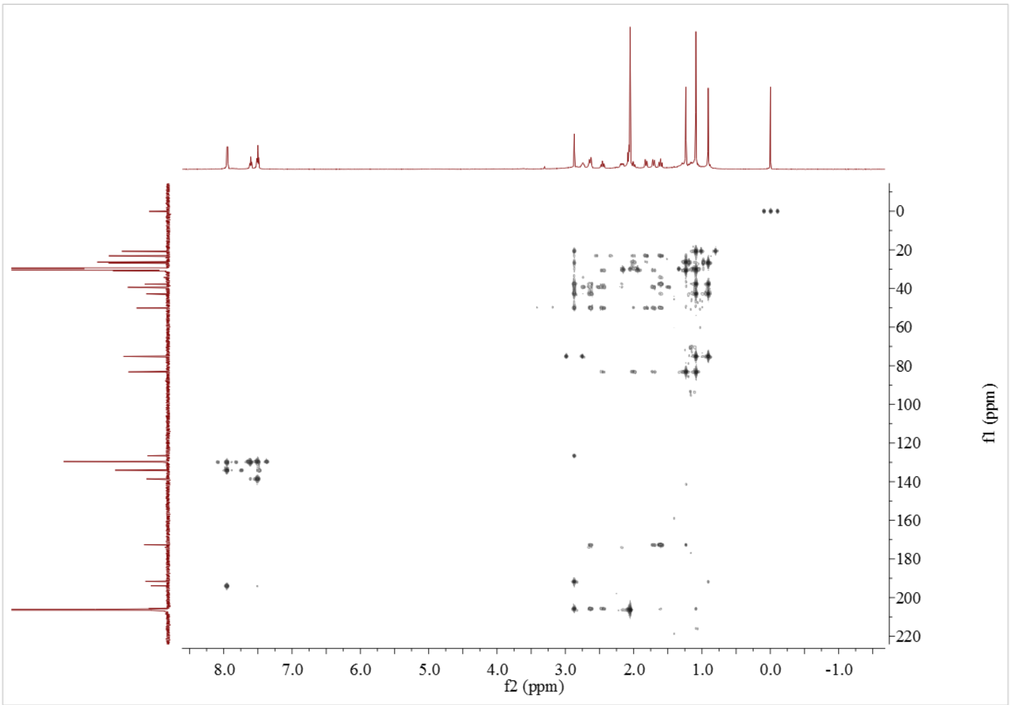

Figure S58. HMBC NMR spectrum (Acetone-d_6, 600 MHz, 150 MHz) of **6**

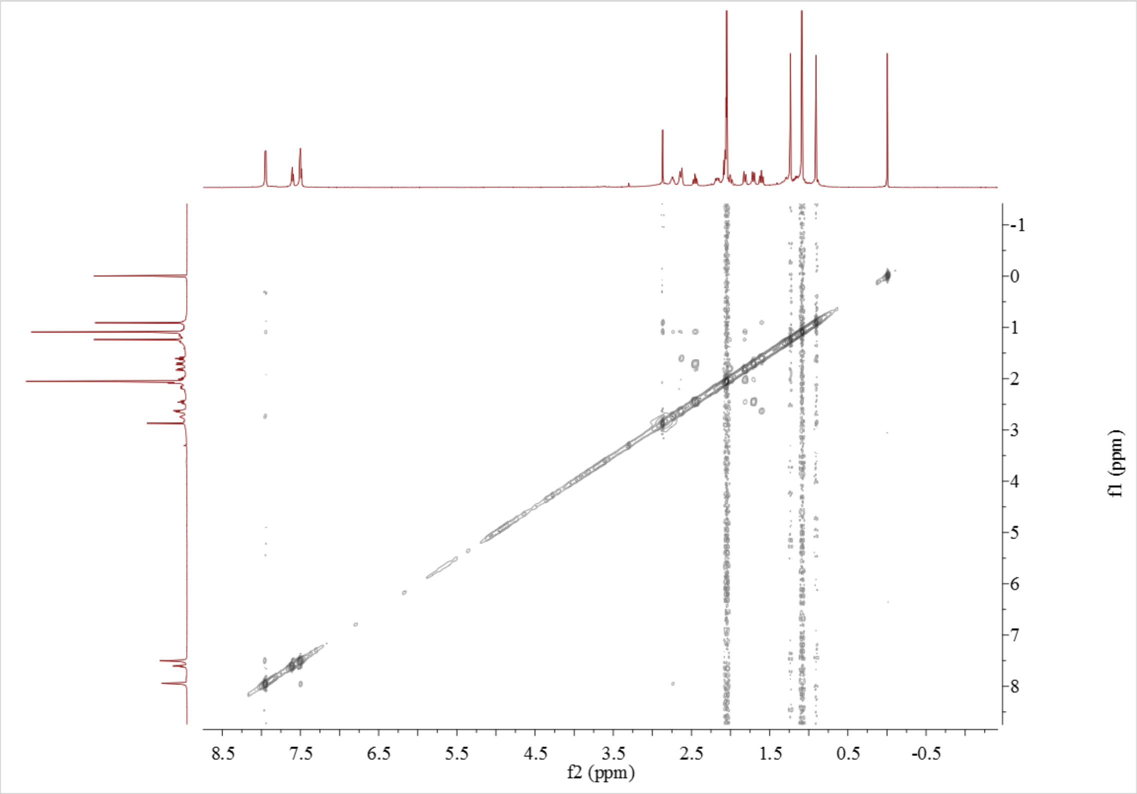

Figure S59. NOSEY NMR spectrum (Acetone-d_6, 600 MHz) of **6**

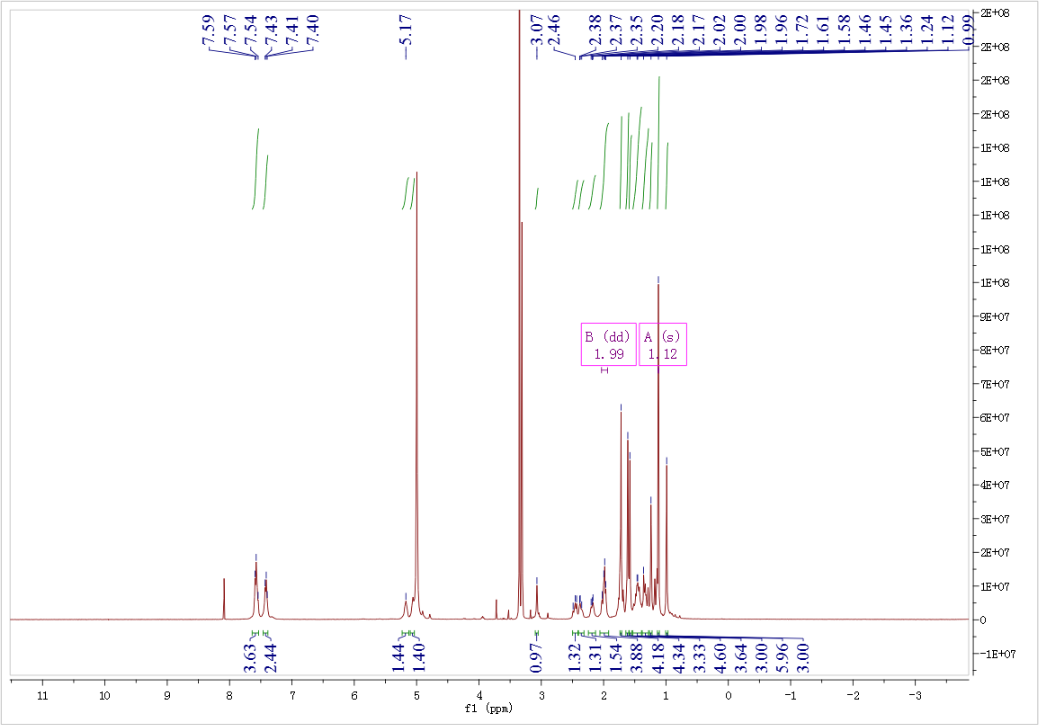

Figure S60. ^{1}H NMR spectrum (CD_3OD/0.1% TFA, 400 MHz) of **7**

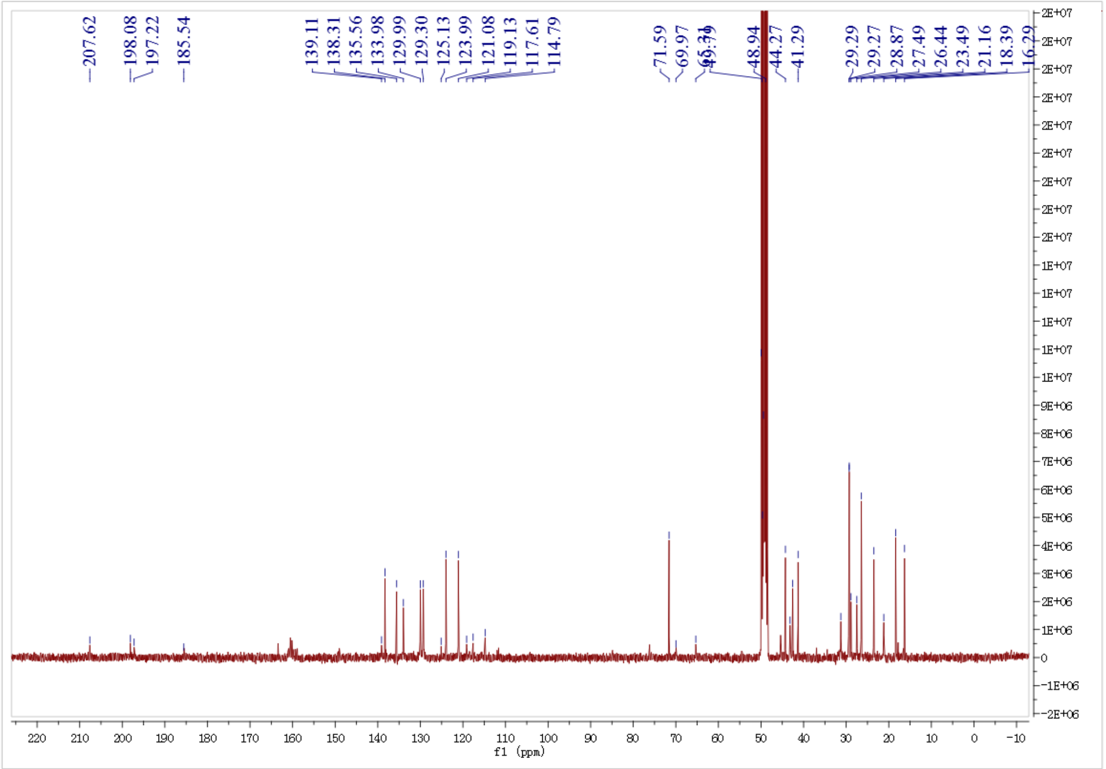

Figure S61. ^{13}C NMR spectrum (CD_3OD/0.1% TFA, 100 MHz) of **7**

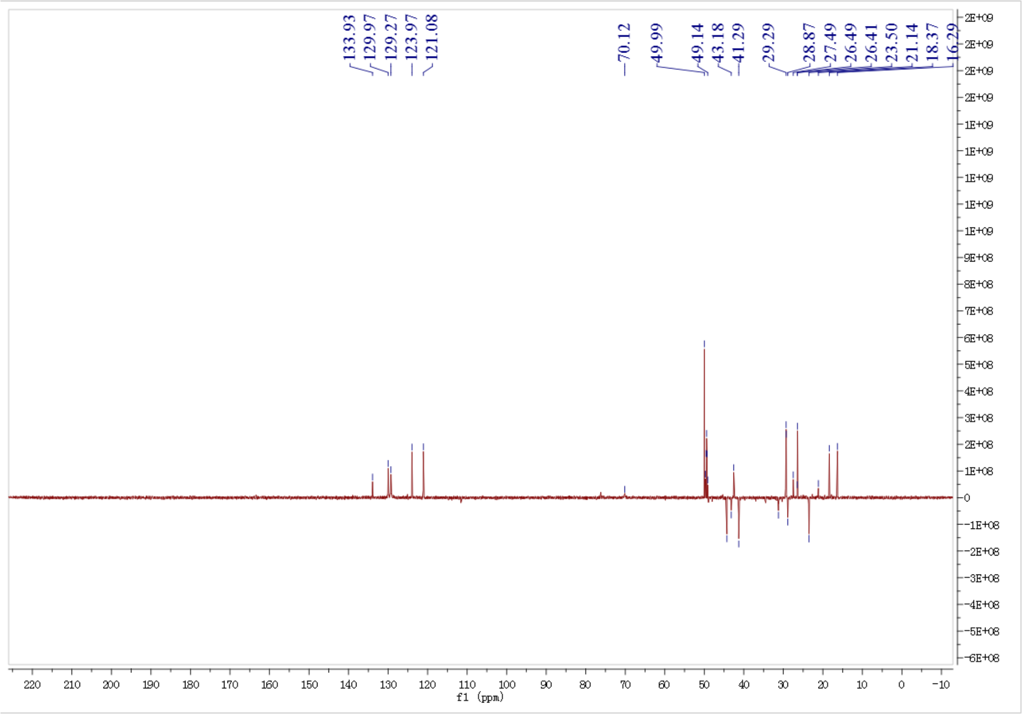

Figure S62. DEPT NMR spectrum (CD_3OD/0.1% TFA, 100 MHz) of **7**

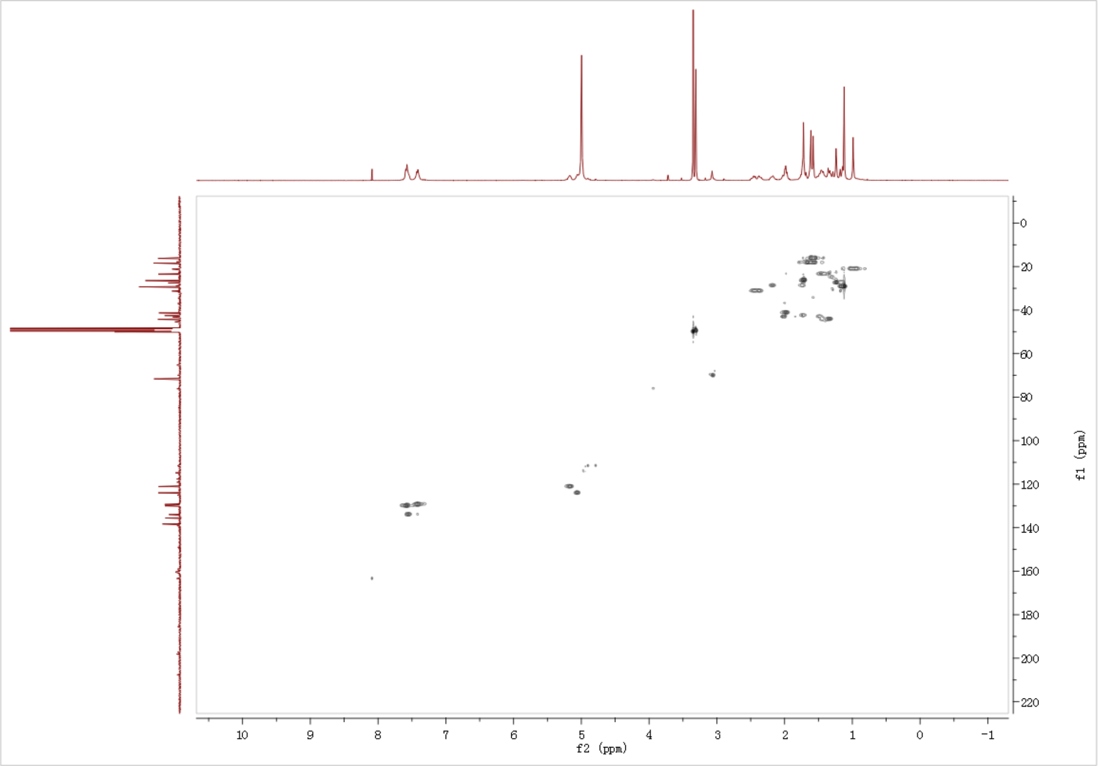

Figure S63. HSQC NMR spectrum (CD_3OD/0.1% TFA, 400 MHz, 100 MHz) of **7**

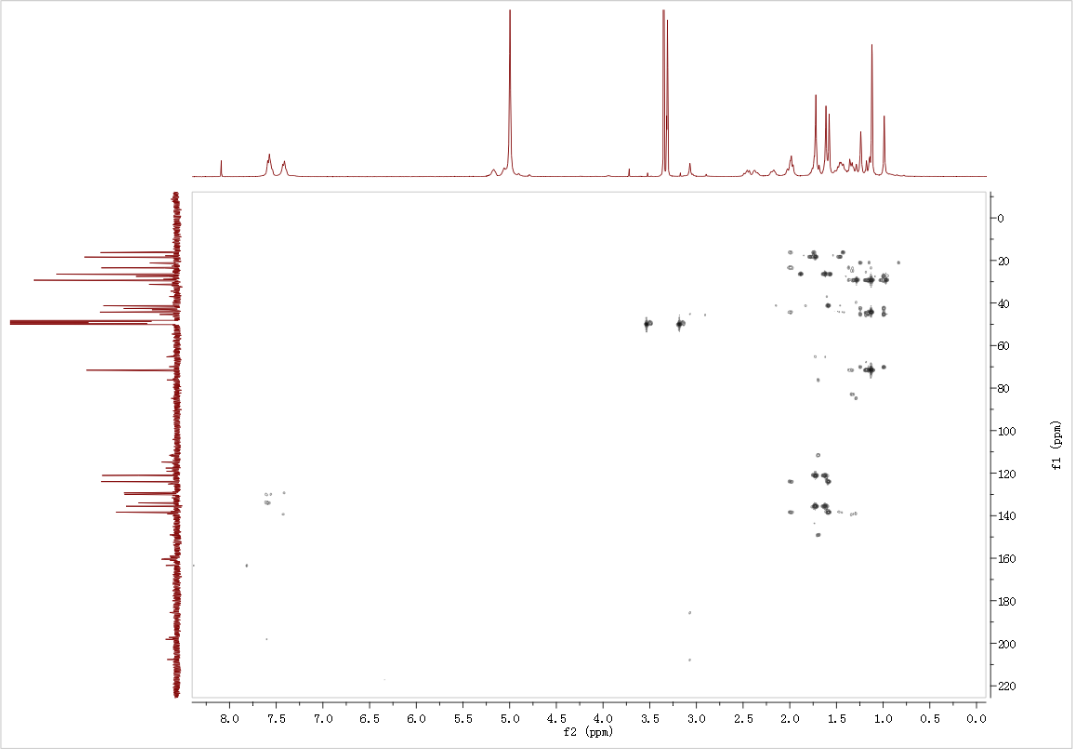

Figure S64. HMBC NMR spectrum (CD_3OD/0.1% TFA, 400 MH$_Z$, 100 MH$_Z$) of **7**

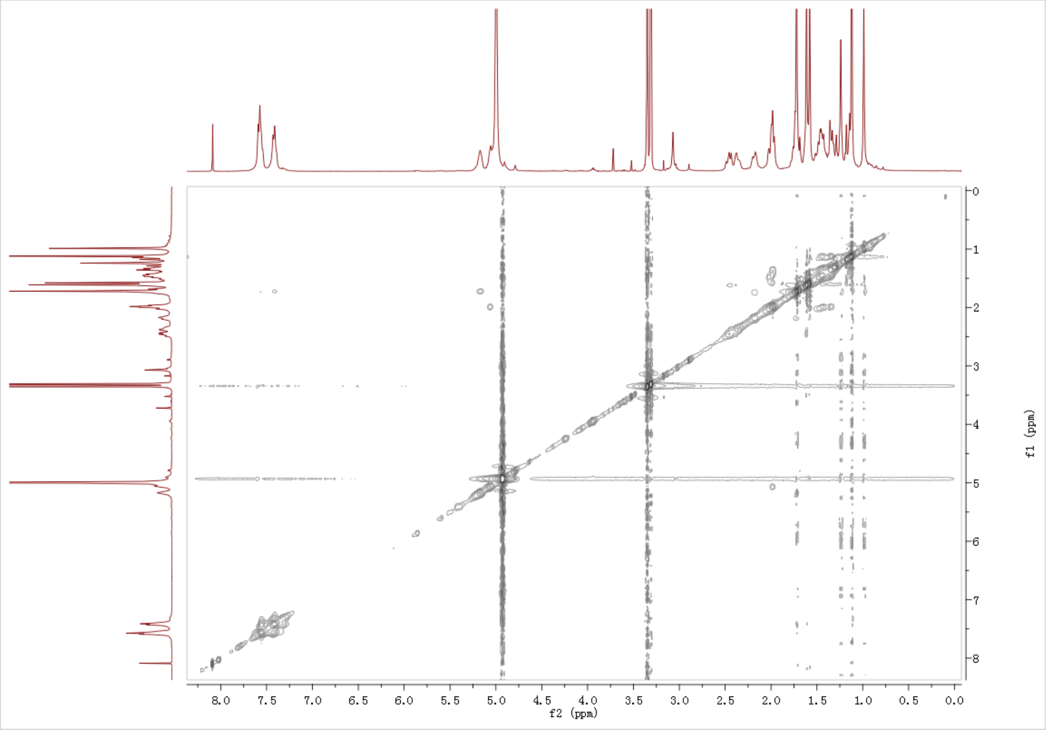

Figure S65. NOSEY NMR spectrum (CD_3OD/0.1% TFA, 400 MH$_Z$) of **7**

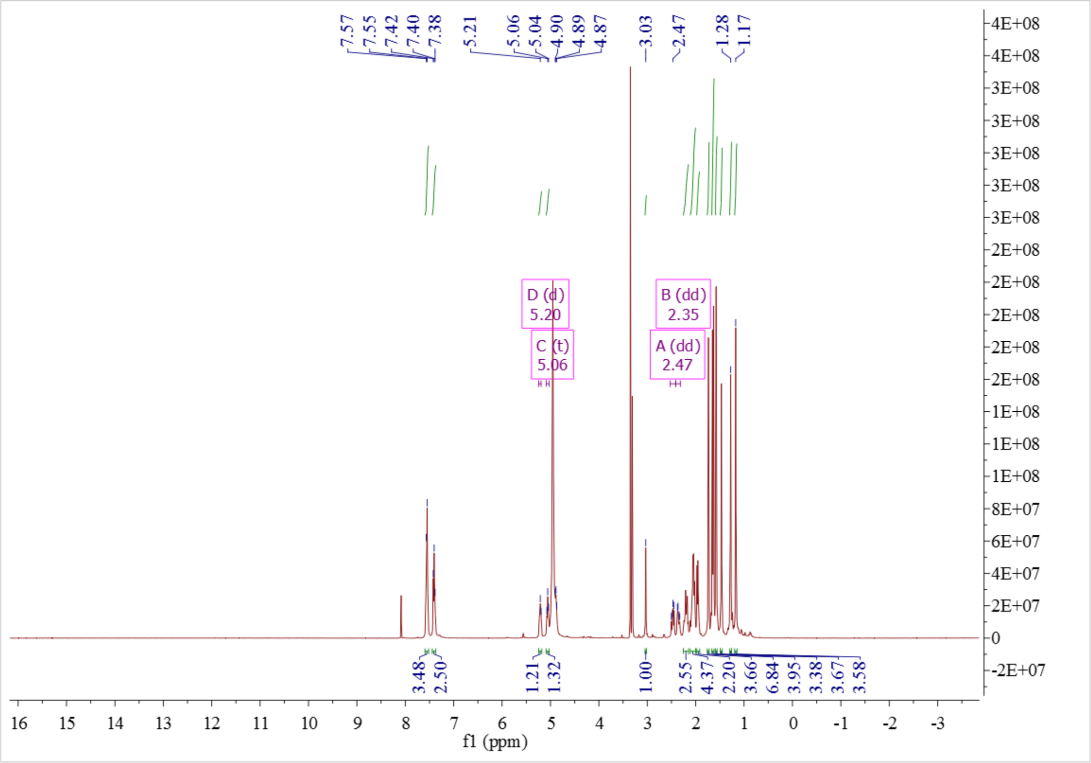

Figure S66. ^{1}H NMR spectrum (CD_3OD/0.1% TFA, 400 MHz) of **8**

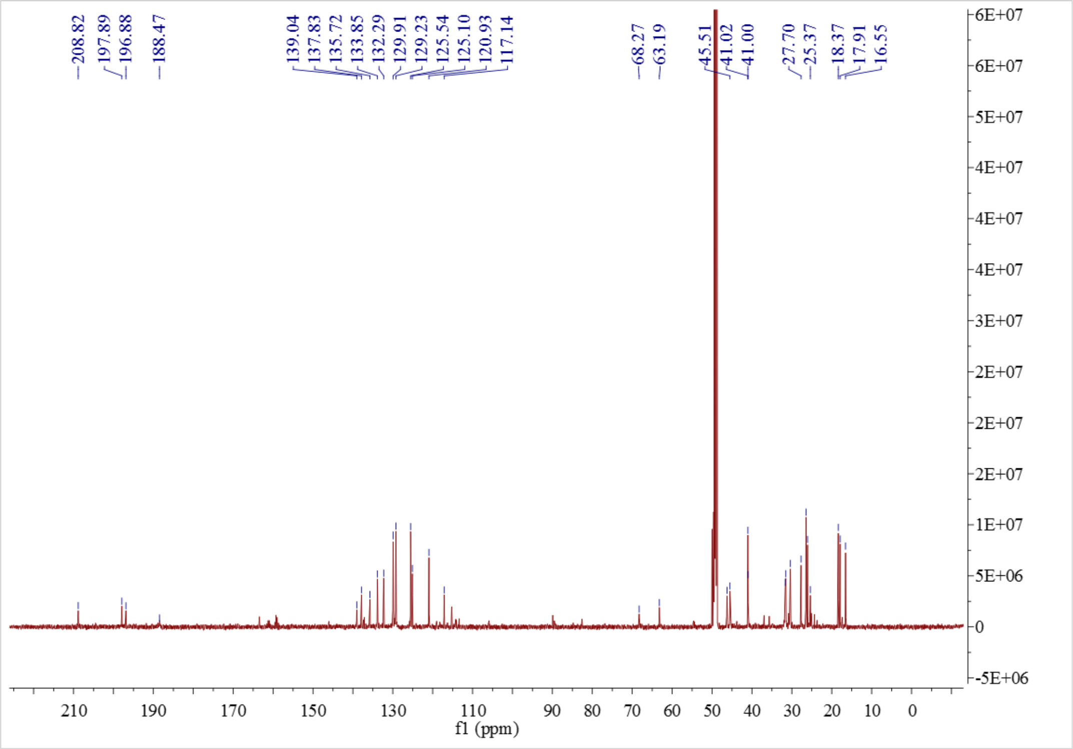

Figure S67. ^{13}C NMR spectrum (CD_3OD/0.1% TFA, 100 MHz) of **8**

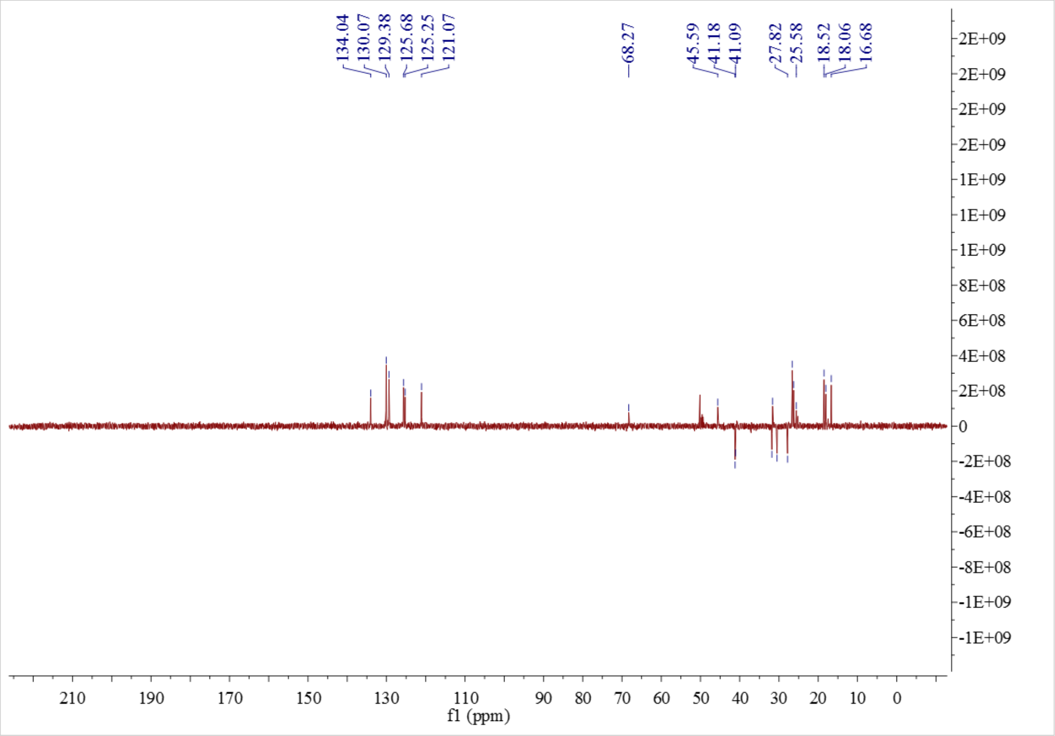

Figure S68. DEPT NMR spectrum (CD_3OD/0.1% TFA, 100 MHz) of **8**

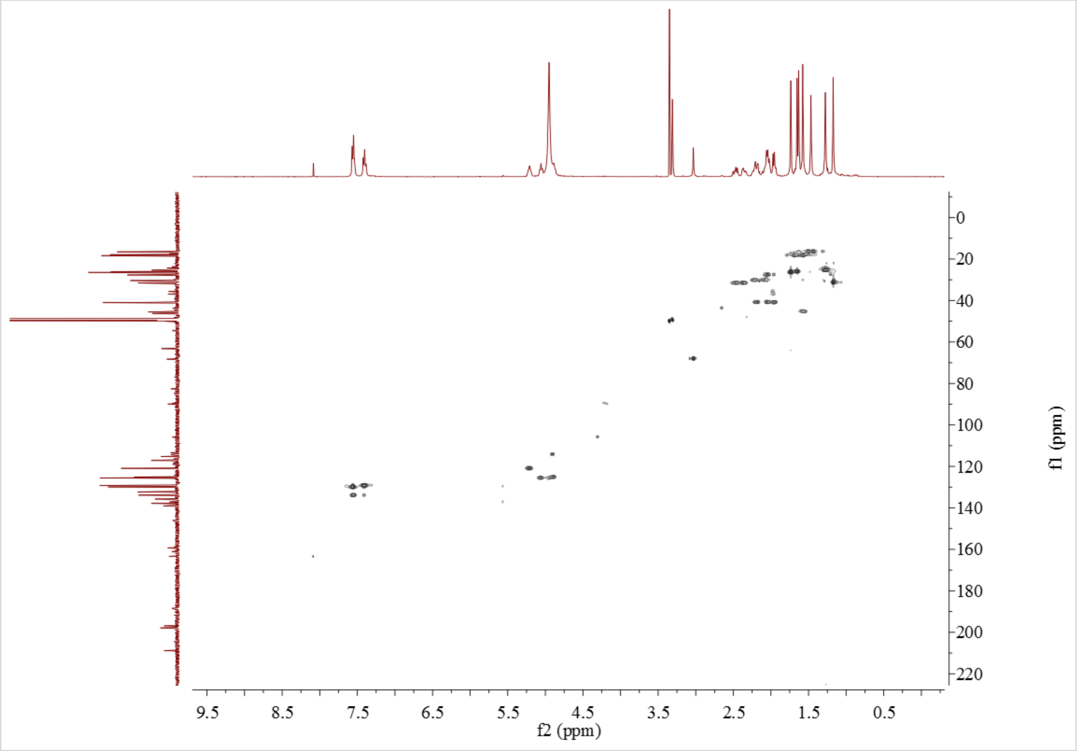

Figure S69. HSQC NMR spectrum (CD_3OD/0.1% TFA, 400 MHz, 100 MHz) of **8**

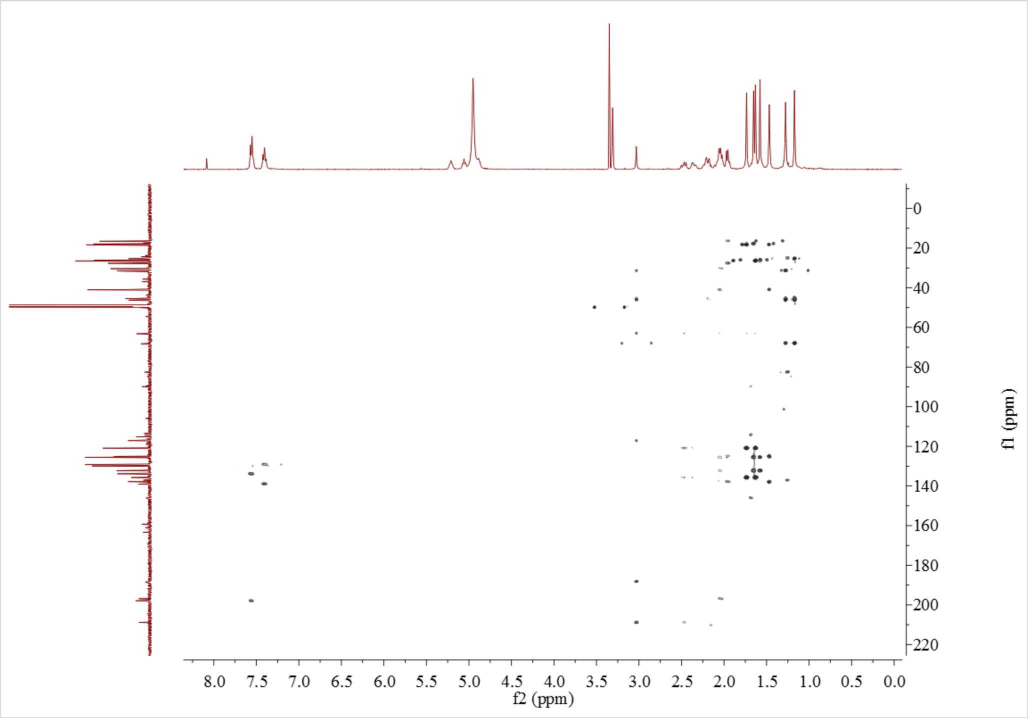

Figure S70. HMBC NMR spectrum (CD_3OD/0.1% TFA, 400 MHz, 100 MHz) of **8**

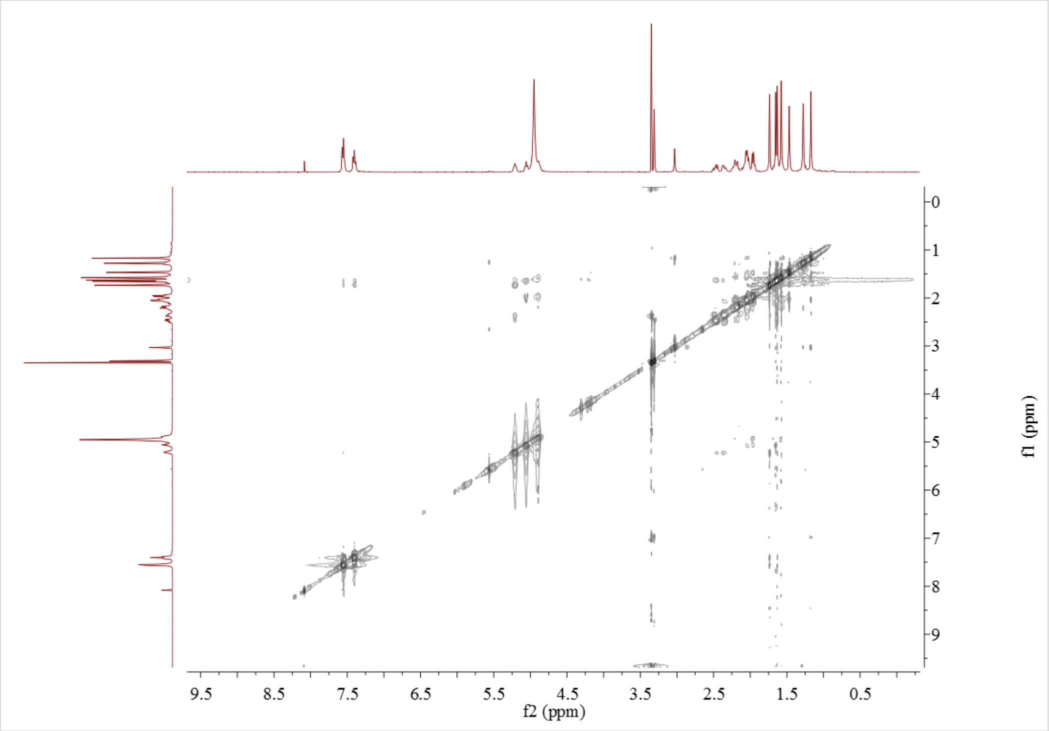

Figure S71. NOSEY NMR spectrum (CD_3OD/0.1% TFA, 400 MHz) of **8**
